# Mesenchymal stromal cells for the prophylaxis and treatment of graft-versus-host disease—a meta-analysis

**DOI:** 10.1186/s13287-020-01592-z

**Published:** 2020-02-18

**Authors:** Cynthia Morata-Tarifa, María del Mar Macías-Sánchez, Antonio Gutiérrez-Pizarraya, Rosario Sanchez-Pernaute

**Affiliations:** Andalusian Network for the Design and Translation of Advanced Therapies, Américo Vespucio 15 S2, 41092 Seville, Spain

**Keywords:** Meta-analysis, Acute graft-versus-host disease, Chronic graft-versus-host disease, Mesenchymal stromal cells, Prophylaxis, Treatment, Cell therapy, Hematopoietic stem cell transplantation

## Abstract

**Background:**

Graft-versus-host disease (GvHD) is the main life-threatening complication of allogeneic hematopoietic stem cell transplantation (HSCT). Thirty to 80% of GvHD patients do not respond to first-line treatment and a second-line treatment is not universally established. Based on their immunomodulatory properties, mesenchymal stromal cells (MSC) have been proposed for the prevention and the treatment of GvHD in patients undergoing HSCT. Unfortunately, previous studies reported conflicting results regarding the prophylactic and therapeutic effects of MSC for GvHD. Consequently, we carried out a meta-analysis to clarify whether MSC administration can improve the dismal outcome of these patients.

**Methods:**

We carried out a systematic review and selected studies (2004–2019) reporting data about the administration of allogeneic MSC for the prevention (*n* = 654 patients) or treatment of acute (*n* = 943 patients) or chronic (*n* = 76 patients) GvHD after HSCT. Our primary outcome was overall survival at the last follow-up. The secondary outcomes were the response and development of GvHD. Subgroup analyses included age, MSC dose, first infusion day after HSCT, number of organs and organ-specific involvement, acute GvHD grade (I–IV), and chronic GvHD grade (limited or extensive).

**Results:**

Patients infused with MSC for GvHD prophylaxis showed a 17% increased overall survival (95% CI, 1.02–1.33) and a reduced incidence of acute GvHD grade IV (RR = 0.22; 95% CI, 0.06–0.81) and chronic GvHD (RR = 0.64; 95% CI, 0.47–0.88) compared with controls. Overall survival of acute GvHD patients (0.50; 95% CI, 0.41–0.59) was positively correlated with MSC dose (*P* = 0.0214). The overall response was achieved in 67% (95% CI, 0.61–0.74) and was complete in 39% (95% CI, 0.31–0.48) of acute patients. Organ-specific response was higher for the skin. Twenty-two percent (95% CI, 0.16–0.29) of acute patients infused with MSC developed chronic GvHD. Sixty-four percent (95% CI, 0.47–0.80) of chronic patients infused with MSC survived; the overall response was 66% (95% CI, 0.55–0.76) and was complete in 23% (95% CI 0.12–0.34) of patients.

**Conclusions:**

Our meta-analysis indicates that allogeneic MSC could be instrumental for the prophylaxis and treatment of GvHD. Future trials should investigate the effect of the administration of MSC as an adjuvant therapy for the treatment of patients with GvHD from the onset of the disease.

## Introduction

Allogeneic hematopoietic stem-cell transplantation (HSCT) is used primarily for the treatment of hematological malignant and nonmalignant disorders [1]. The main life-threatening complication of allogeneic HSCT is graft-versus-host disease (GvHD), an immunological condition produced by donor T cells which respond to genetically defined host proteins, being the most important the human leucocyte antigens (HLAs) [2]. The disparity at loci that encode minor histocompatibility antigens, created by sequence and structural variations within the genome, can also elicit GvHD even in HLA-identical sibling donor/recipient pairs [3]. The total number of minor histocompatibility loci is large, ensuring that all donor/recipient pairs will be mismatched for many minor H antigens [3, 4]. Therefore, despite the use of GvHD prophylaxis [5] and the improvement in HLA matching techniques, acute (a) GvHD incidence is approximately 40% in allogeneic HSCT from HLA-identical siblings [6], and it can reach 80% for HLA-mismatched unrelated-donor [2]. In addition, 35–50% of patients undergoing HSCT develop chronic (c) GvHD, a long-term complication of HSCT, which can occur de novo or as a progression of aGvHD [7].

Corticosteroids remain the most widely employed first-line treatment for aGvHD, with non-response rates ranging from 30 to 50% [8–10]. For cGvHD, the first-line treatment includes corticosteroids and a calcineurin inhibitor, with non-response rates ranging from 60% to 80% [11, 12]. Although an extensive variety of second-line treatments are available [8, 12–15], none is universally agreed upon. Additionally, the long period of exposure to non-specific immunosuppressive therapy produces long-term side effects with overall dismal prognosis of these patients [1, 16–19].

Several studies support the infusion of mesenchymal stromal cells (MSC) for either the prevention or the treatment of GvHD for patients who undergo HSCT. Their capacity to induce a shift from a pro- to an anti-inflammatory environment [20] makes them suitable to treat disorders related with alloreactivity such as GvHD. In fact, the use of allogeneic bone marrow-derived MSC is approved for the treatment of aGvHD in pediatric patients in Canada (Prochymal, Mesoblast International Sarl) and for pediatric and adult patients in Japan (Temcell, JCR Phamaceuticals Co. Ltd). The first report that demonstrated the clinical efficacy of MSC for the treatment of GvHD was published in 2004 [21]. Since then, various studies have reported conflicting results. Due to the high incidence of GvHD in patients treated with HSCT, we have carried out a meta-analysis of available data to clarify whether the administration of allogeneic MSC for the prophylaxis or treatment of GvHD can improve the outcome of patients receiving allogeneic HSCT.

## Methods

### Search strategy and selection criteria

This systematic review and meta-analysis is reported according to the PRISMA statement. We comprehensively searched for full-text published studies with no publication date restriction. Studies reporting data about the administration of allogeneic MSC for the prophylaxis or treatment of GvHD after HSCT were identified using MEDLINE and Cochrane Central Register of Controlled Trials (CENTRAL). The last search was carried out on October 15, 2019. The search strategy is detailed in Table S1 (see Additional file, pp. 29–30). Only full-text clinical studies published in English were included. We excluded meta-analysis and reviews. Studies reporting data for less than three patients were excluded. Only reports containing data for survival or response were included.

### Data extraction and quality assessment

Study selection was performed by two authors (C.M-T, M.M-S), examining the full text of potentially relevant studies and applying eligibility criteria to select the included studies. Data were extracted in an Excel template. Information obtained from included studies enclosed a description of the patients, characteristics of the treatment administrated and outcome. C.M-T extracted the data from the included studies and M.M-S revised the extracted data. Disagreements were solved by discussion and consensus between the two reviewer’s authors. To evaluate the methodological quality of the studies included in the meta-analysis, we employed the risk of bias assessment tool for non-randomized studies (RoBANS) [22], since most of the included studies were non-randomized.

### Outcomes

Our primary outcome was overall survival (OS) at the last follow-up. Median and ranges for the follow-up times are included in the corresponding supplementary Tables S2, S3, and S4. We established the development of aGvHD and cGvHD at last follow-up as secondary outcomes for prophylaxis. For studies related to the treatment of aGvHD or cGvHD, we considered as secondary outcomes the overall, complete, and partial clinical responses (OR, CR, and PR). For studies using MSC as treatment of aGvHD, we established as secondary outcomes the development of cGVHD (limited and extensive) and organ- and grade-specific responses. A secondary outcome for all the studies was dose-specific responses. We based the definition of CR and PR on consensus criteria at the time of the study.

### Statistical analysis

We carried out the statistical analysis using Review Manager 5.3 (RevMan 5.3). Meta-regression is a regression method in which each datapoint is a study (not a patient). We did subgroup analyses for survival and response based on age (adults and pediatric population); dose of MSC administrated; and first infusion day after HSCT, number of organs and organ-specific involvement, aGvHD grade (I–IV), and cGvHD grade (limited or extensive).

We used Mantel-Haenszel test for dichotomous analysis or inverse-variance to pool outcomes across studies. The risk ratio (RR) and risk differences (RD) and 95% confidence interval (CI) were used to dichotomous data and pool outcomes data, respectively. Random-effects model was used to consider the differences between individual study effects to estimate the effect. *P* value ≤ 0.05 was considered statistically significant. For studies that reported median of MSC doses, we calculated the mean as previously described [23]. The heterogeneity between studies was assessed using *I*^2^ test. Additionally, Egger test was performed to assess the asymmetry/bias of each funnel plot [24]. Publication bias analysis was determined with SPSS v15.0.

## Results

**Fig. 1:**
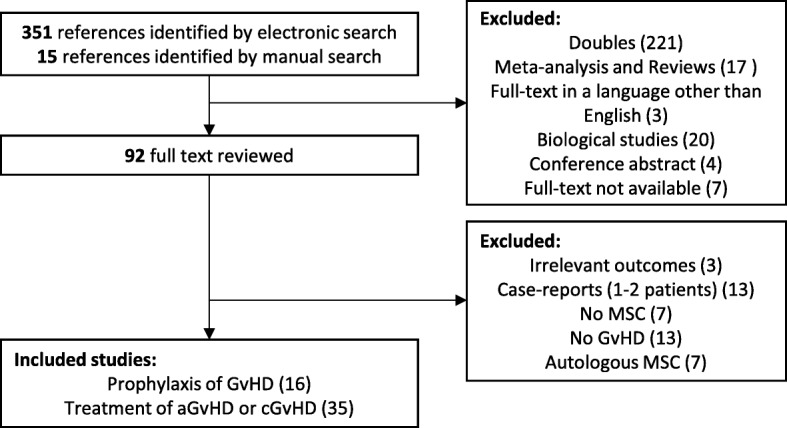
Selection of studies

We identified 364 references by electronic and manual search. Of these, 92 full-text studies were evaluated according to the eligibility criteria. Finally, 51 studies were included, 16 related to the administration of allogeneic MSC for the prophylaxis of GvHD and 35 for the treatment of aGvHD and/or cGVHD (Fig. 1).

### Mesenchymal stromal cells for the prevention of GvHD in patients undergoing HSCT

Characteristics of the included prophylaxis-related studies are reported in Table S2 (see Additional file, pp. 31–32). These studies comprised 654 patients, 298 patients who received only HSCT (control group) and 356 patients infused with MSC (MSC group) for GvHD prevention. Included studies comprised data from children (< 18 years old, *n* = 8 studies), adults (≥ 18 years old, *n* = 4 studies) and from both adults and children (*n* = 4 studies). Five of the included reports did not include the control group. Studies varied highly in terms of MSC doses (0.03–10.12 × 10^6^ MSC/kg). The MSC sources were bone marrow (BM, *n* = 12 studies) and umbilical cord (UC, *n* = 4 studies), the majority of them from a different donor than HSCT. For most studies, a single dose of MSC was administered on the same day than HSCT while in 4 studies, a second dose of MSC was also administered on day + 2 [25], + 14 [26, 27] or + 21 [28] after HSCT. In two studies, the single administration of MSC was carried out after HSCT, on day + 28 (19–54) [29] or after more than 4 months after transplantation [30]. Any of the studies included to evaluate the effect of MSC infusion for the prophylaxis of GvHD showed low risk for the six domains of RoBANS (Figure S1, see Additional file, pp. 4). All the studies had not blinded the outcome, except in the double-blinded study reported by Gao et al. [30], which presented an unclear risk for detection bias since the blinding of outcome assessors was not stated. Nine studies showed low risk in all other RoBANS criteria.

**Fig. 2:**
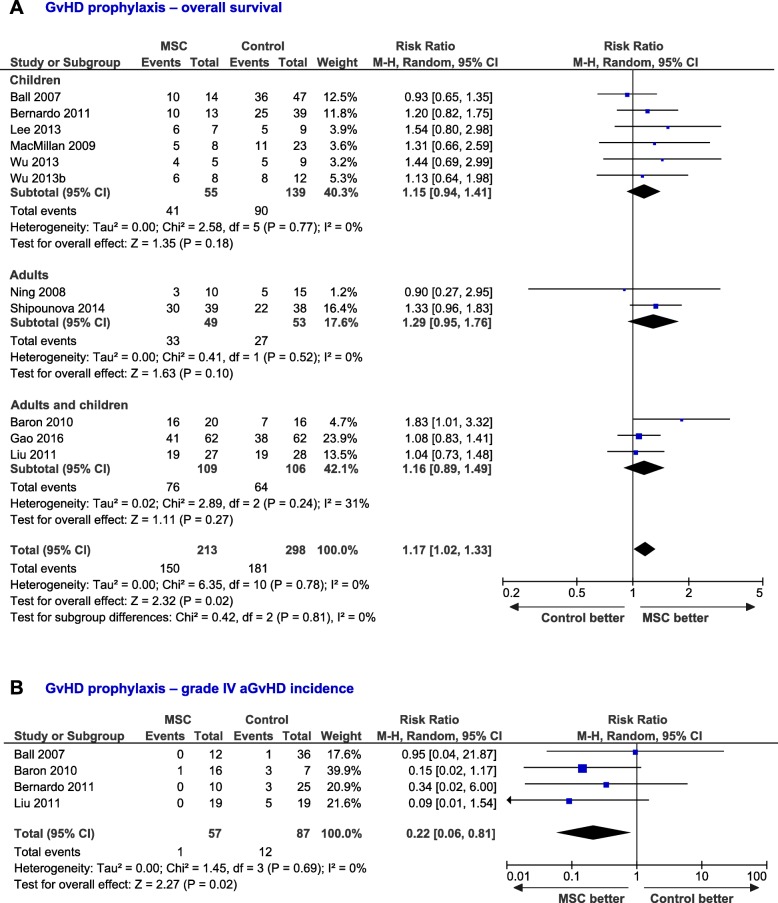
Forest-plots of the outcome of MSC vs control patients. **a** Dichotomous analysis for the overall survival of patients infused with MSC vs control patients at last follow-up. **b** Risk to suffer grade IV aGvHD for patients from the MSC group compared with control patients. Dots and black lines represent the effect and 95% CI of individual studies. Black diamonds represent the overall effect size. Weights are from random-effects analysis

At last follow-up, 73% (95% CI, 0.67–0.79, *I*^2^ = 42%) and 59% (95% CI, 0.52–0.66, *I*^2^ = 35%) of the patients from the MSC (Figure S2A) and control (Figure S2B) groups were alive, respectively (see Additional file, pp. 5). OS did not differ between children and adults for the control (*P* = 0.28) and MSC (*P* = 0.33) groups. Funnel plots show OS distribution for individual studies from the control (Figure S3A) and MSC (Figure S3B) groups (see Additional file, pp. 6). Egger test showed no evidence of publication bias for the MSC (*P* = 0.259) and control (*P* = 0.100) groups. However, Egger test showed some degree of publication bias on subgroup analyses. It was not possible to test the publication bias for the subgroup *adults* in the MSC group due to the low number of studies. The dichotomous analysis of 11 studies containing data related to OS from both control (*n* = 298 patients) and MSC (*n* = 213 patients) groups showed that the administration of MSC and HSCT was associated with a 17% increased OS (95% CI, 1.02–1.33, *I*^2^ = 0%, Fig. 2a).

**Fig. 3:**
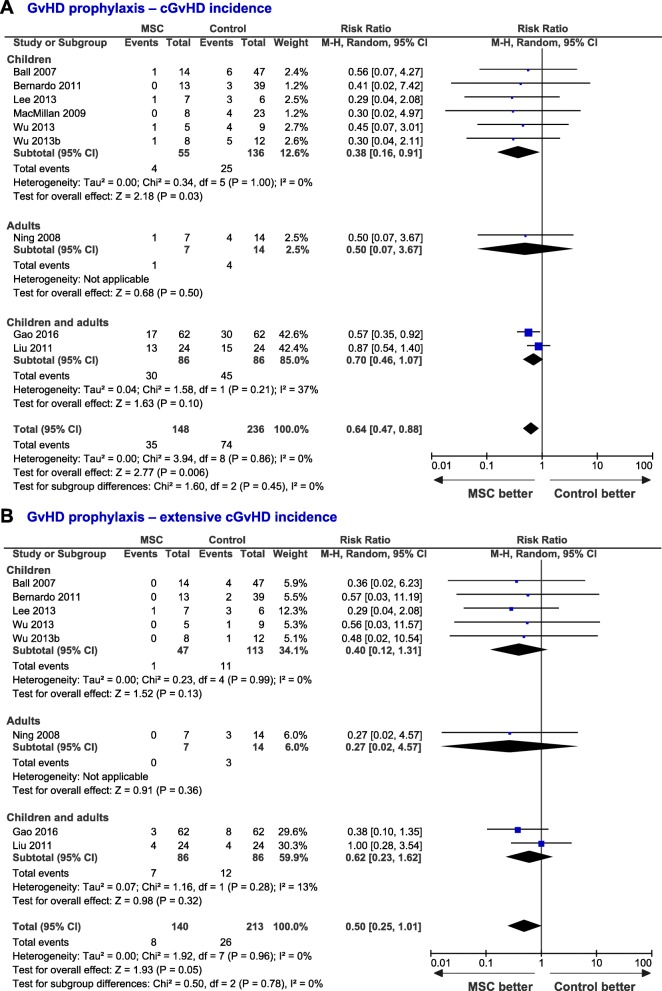
Forest-plots showing the risk to suffer overall (**a**) and extensive (**b**) cGvHD in patients from the MSC group compared with control patients. Dots and black lines represent the effect and 95% CI of individual studies. Black diamonds represent the overall effect size. Weights are from random-effects analysis

Data related to the incidence of aGvHD in the control (*n* = 235 patients) and MSC (*n* = 150 patients) groups were collected from 10 studies. Patients from the MSC group tended to suffer less aGvHD compared with the control group (RR = 0.84; 95% CI, 0.66–1.07, *I*^2^ = 0%, Figure S4, pp. 7). The incidence of severe aGvHD (grades III–IV) was not significantly different between treatments (RR = 0.53; 95% CI, 0.20–1.42, *I*^2^ = 0%; Figure S5, pp. 8). Although most studies report together grades III and IV, the analysis of four studies comprising 144 patients showed that the administration of MSC was associated with a lower incidence of grade IV aGvHD, compared with the control group (RR = 0.22; 95% CI, 0.06–0.81, Fig. 2b). In addition, the analysis of nine studies (MSC, *n* = 148 patients; control, *n* = 236 patients) showed that the infusion of MSC was associated with a reduced cGvHD incidence (RR = 0.64; 95% CI, 0.47–0.88, *I*^2^ = 0%, Fig. 3a) and a trend to a lower incidence of extensive cGvHD (RR = 0.50; 95% CI, 0.25–1.01, *P* = 0.05, Fig. 3b). The risk to suffer acute and chronic GvHD did not differ between adults and children. For the MSC group, OS and GvHD incidence were not correlated with MSC dose (Figure S6, pp. 9).

### Mesenchymal stromal cells for the treatment of steroid-refractory GvHD

Next, we analyzed the outcome of patients who were infused with MSC for the treatment of aGvHD (*n* = 943 patients) or cGvHD (*n* = 76 patients). The characteristics of the 35 studies reporting data for the treatment of aGvHD and/or cGvHD are summarized in Table S3 (see Additional file, pp. 33–36). Only six of these studies reported data of interest for control patients (aGvHD, *n* = 182 patients; cGvHD, *n* = 14 patients; Table S4, pp. 37). Included studies reported data from children (*n* = 10 studies), adults (*n* = 22 studies), and both children and adults (*n* = 8 studies). Studies varied greatly in terms of MSC dose (aGvHD, 0.22–6.81; cGvHD, 0.6–2.28 × 10^6^ MSC/kg), dose number (aGvHD, 1–10; cGvHD, 1–11), and time for first infusion after HSCT (aGvHD, + 2.5 to + 124.7 days; cGvHD, + 64.1 days to + 45.1 months). The sources of the infused MSC were BM (94.90%), adipose tissue (AD, 2.94%), or BM and AD (2.16%), most of them from a different donor than the HSCT. Table S5 shows the number of patients with aGvHD for individual studies which report the grade and organs implicated (see Additional file, pp. 38–40). Most of the included patients suffered grade III–IV (82.99%). Since Dalowski et al. [31] included data from von Bonin et al. [32], data from this last study were included only when it considered subgroups or outcomes that were not reported by Dalowski et al. Only one of the included studies to analyze the effect of the administration of MSC for the treatment of GvHD showed low risk for the six domains of RoBANS. Eighteen studies showed low risk for five RoBANS criteria (Figure S7, pp. 10).

**Fig. 4:**
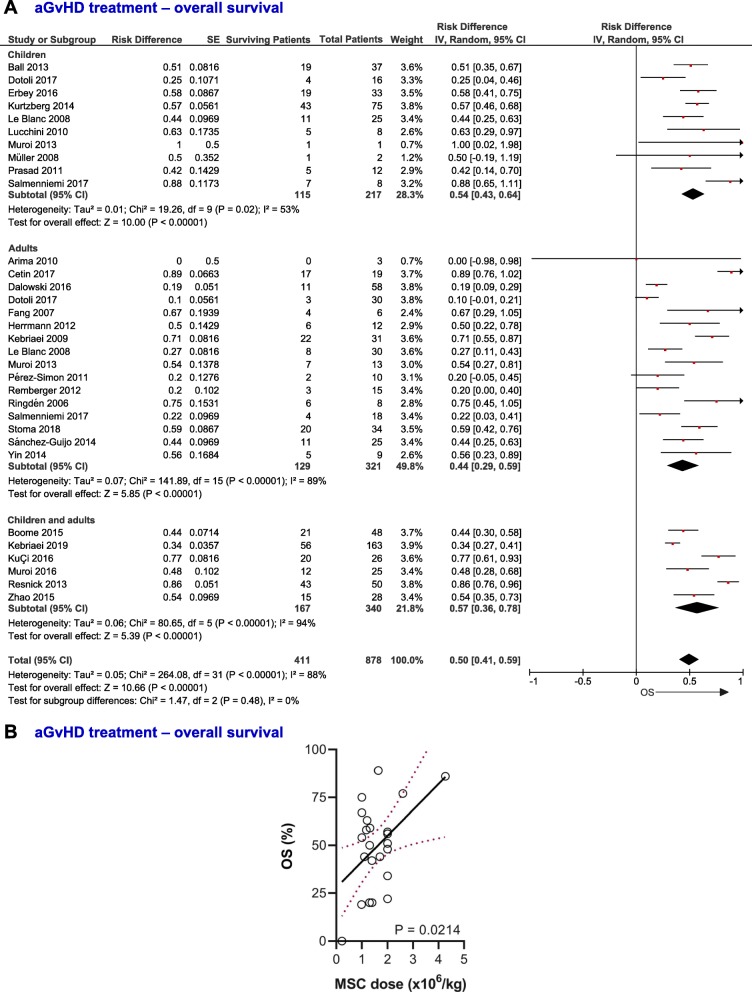
Overall survival of aGvHD patients. **a** Forest-plot showing the overall survival of patients with aGvHD infused with MSC. Dots and black lines represent the effect and 95% CI of individual studies. Black diamonds represent the overall effect size. Weights are from random-effects analysis. **b** Correlation between overall survival of patients with aGvHD at last follow-up and MSC dose

**Fig. 5:**
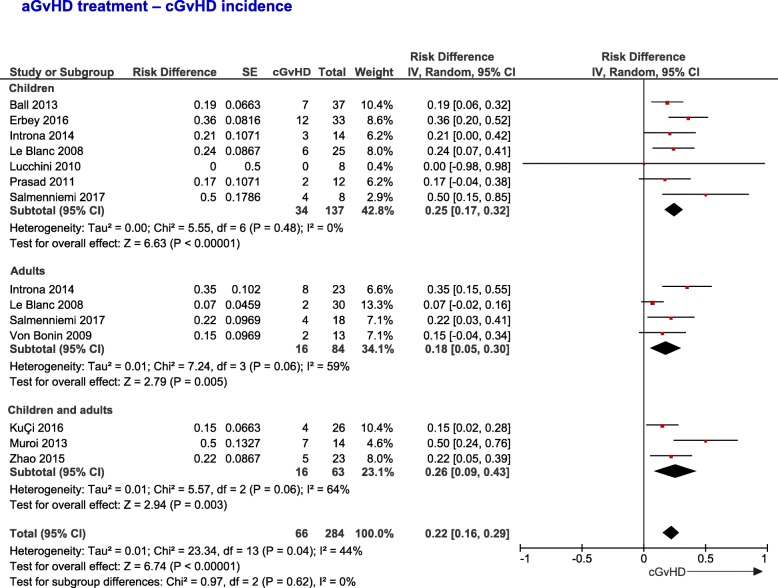
Forest-plot showing the cGvHD incidence of patients with aGvHD infused with MSC. Dots and black lines represent the effect and 95% CI of individual studies. Black diamonds represent the overall effect size. Weights are from random-effects analysis

The analysis of 26 studies showed that 50% (95% CI, 0.41–0.59, *I*^2^ = 88%) of aGvHD patients treated with MSC (*n* = 878 patients) were alive at last follow-up (Fig. 4a). On the other hand, the analysis of five studies (*n* = 182 patients) showed that only 25% (95% CI, 0.11–0.39, *I*^2^ = 81%) of the patients from the control group were alive at the last follow-up (Figure S8A, pp. 11). The OS for the different treatment groups was significantly different (*P* = 0.0005). The analysis of five studies containing data for the OS of both control and MSC groups showed a trend to a higher OS for patients infused with MSC (RR = 1.33; 95% CI, 0.84–2.10, *I*^2^ = 51%, Figure S8B, pp. 11). Regarding the MSC group, the OS did not differ between children and adults (*P* = 0.29), with respect to aGvHD grade (*P* = 0.73, Figure S9), the number of organs involved (*P* = 0.50, Figure S10), or organ-specific involvement (*P* = 0.37, Figure S11), although patients with skin implication tended to survive more (0.64; 95% CI, 0.52–0.77) compared to those with gut (0.51; 95% CI, 0.35–0.66) and liver (0.57; 95% CI, 0.41–0.72) involvement (see Additional file, pp. 12–14). In the case of multiorgan implication, OS did not differ between subgroups (Figure S12, pp. 15). The analysis of data related to OS according to the grade and organs involved are summarized in Table S6 (see Additional file, pp. 41). Interestingly, the OS of patients with aGvHD infused with MSC was positively correlated with the MSC dose (*P* = 0.0214, Fig. 4b) and did not differ in relation to the first day of infusion of MSC from HSCT (*P* = 0.1034, Figure S13, pp. 16). Twenty-two percent (95% CI, 0.16–0.29, *I*^2^ = 44%) of the patients infused with MSC for the treatment of aGvHD developed cGvHD, without differences between children and adults (*P* = 0.62, Fig. 5).

For patients with aGvHD, 67% of 887 patients (95% CI, 0.61–0.74, *I*^2^ = 74%) responded to the MSC infusion (Figure S14A) and 39% (95% CI, 0.31–0.48, *I*^2^ = 81%) achieved CR (Figure S14B, pp. 17). The proportion of responders did not differ between children and adults nor with respect to the aGvHD grade (*P* = 0.17, Figure S15, pp. 18–19) or number of organs affected (OR, *P* = 0.14, Figure S16; CR, *P* = 0.89, Figure S17, pp. 20–21). However, the proportion of complete responders tended to be lower for patients with grade III–IV compared to grade II (RR, 0.76; 95%CI, 0.54–1.05; *I*^2^ = 0%, Figure S18, pp. 22). Regarding to organ-specific response, the proportion of patients that achieved OR (Figure S19A) and CR (Figure S19B) was higher in skin compared with liver and gut implication (see Additional file, pp. 23). In addition, the OR was significantly reduced in children (0.61; 95% CI, 0.51–0.71) compared with adults (0.76; 95% CI, 0.68–0.85) when the gut was affected (*P* = 0.03, Figure S20, pp. 24). In the case of multiorgan involvement, the proportion of patients that achieved OR and CR did not differ between subgroups. Subgroup analysis for OR and CR are summarized in Table S7 and S8, respectively (see Additional file, pp. 42–43). No significant correlation was found between the proportion of responder patients and day of MSC infusion after HSCT (Figure S21A) or MSC dose (Figure S21B, pp. 25).

**Fig. 6:**
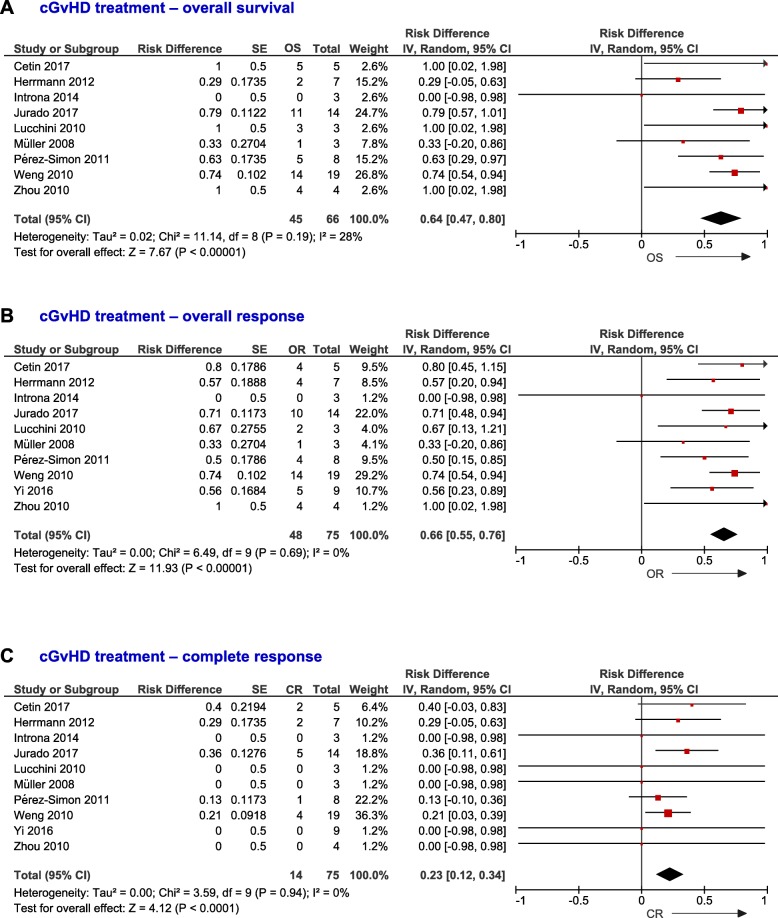
Forest plots of overall survival (**a**), overall (**b**) and complete (**c**) responses of patients with cGvHD infused with MSC. Dots and black lines represent the effect and 95% CI of individual studies. Black diamonds represent the overall effect size. Weights are from random-effects analysis

Data related to the effect of the MSC infusion in the outcome of patients with cGvHD were collected from 10 studies (*n* = 75 patients). At the longest follow-up, 64% (95% CI, 0.47–0.80, *I*^2^ = 28%) of chronic patients were alive (Fig. 6a). Sixty-six percent (95% CI, 0.55–0.76, *I*^2^ = 0%) and 23% (95% CI, 0.12–0.34, *I*^2^ = 0%) achieved OR (Fig. 6b) and CR (Fig. 6c), respectively. A low number of studies reported data about both aGvHD and cGvHD. Data from five and seven studies showed no differences in OS (*P* = 0.74, Figure S22A) and response rates between patients with aGvHD and cGvHD infused with MSC, although patients that suffered cGvHD were less likely to achieve OR (0.77; 95% CI, 0.56–1.06, Figure S22B) and CR (0.66; 95% CI, 0.32–1.36, Figure S22C) than aGvHD patients (see Additional file, pp. 26–27).

## Discussion

Our meta-analysis indicates that the prophylactic administration of allogeneic MSC to patients undergoing HSCT increases the probability of survival, partly because MSC prevent the occurrence of GvHD. A recent meta-analysis [33] including five randomized controlled trials (RCTs) showed no significant differences for all-cause mortality between MSC and no MSC groups, in contrast to our results. We believed that these different results could be due to the lower number of cases and shorter end point of this study. Similar to our meta-analysis, they also showed no significant differences for the incidence of aGvHD (six RCTs) and a reduced cGvHD incidence for the MSC group (six RCTs). Additionally, our meta-analysis indicated that the MSC infusion may be associated with a lower aGvHD grade IV incidence. In addition to GvHD prevention, MSC may promote HSC engraftment and prevent engraftment failure [34–36]. The significant heterogeneity showed for OS in the MSC group across individual studies (*I*^2^ = 42%, *P* = 0.04) indicates that there are other variables that determine the survival rate. Kharbanda et al. [25] reported a low OS and the study was prematurely terminated due to unacceptably high, transplant-related mortality. It is not possible to determine whether a second administration of MSC provides an advantage in the outcome of patients, since only four studies carried out two infusions. Data reported by Ning et al. [37] showed a reduced OS compared with other studies, coincident with the lowest dose of MSC administrated [0.34 (0.03–1.53) × 10^6^ MSC/kg]. Therefore, although in our meta-analysis MSC dose for the prophylaxis of GvHD was not significantly correlated with patient outcome, a minimum MSC dose could be required to obtain an effect. Nonetheless, in order to define such threshold, it will be necessary to carry out a blinded clinical study comparing stratified MSC doses and frequencies of administration.

Conditioning regimen before HSCT is required to provide sufficient immunoablation to prevent graft rejection and reduce the tumor burden. Although full consensus about standard regimen has not been reached within the HCT community, conditioning regimens are classified as high-dose (myeloablative), reduced-intensity, and nonmyeloablative [38]. The conditioning regimen contributes to the remission of the hematological disease but also to toxicity, and therefore, individual differences in the regimen are crucial for the survival of patients. Another factor that adds variability across studies is the degree of matching of the HSCT donor. In fact, aGvHD incidence varies from 40% for HLA-identical siblings [6] to 80% for HLA-mismatched unrelated donors [2, 13]. Only approximately 25% of siblings are HLA-matched, and usually, the only alternatives are unrelated or haploidentical donors [39]. A recent study has reported that post-transplant cyclophosphamide in HLA-haploidentical HSCT is associated with low rates of severe graft-versus-host disease and its efficacy is comparable with HLA-matched HSCT [40].

GvHD drug prophylaxis could also determine the variability in the GvHD incidence. A meta-analysis of 1439 patients showed that the administration of methotrexate (MTX) + tacrolimus decreased the risk of aGvHD compared with MTX + cyclosporin A (CsA). The incidence of aGvHD was lower in both conditions compared with patients treated with CsA alone [41].

Regarding the administration of MSC for the treatment of GvHD, our findings support that MSC increase the OS. According to our results, only 5–30% of patients with steroid-refractory aGvHD survive [42]. Our analysis indicates a higher OS (50%) for those patients infused with MSC for the treatment of aGvHD in a dose-dependent manner. These studies showed a high heterogeneity for OS (*I*^2^ = 88%) and response rates (*I*^2^ = 74%) indicating, as above, that there are other factors involved in the outcome of these patients. Interestingly, patients with aGvHD grade II tended to achieve more frequently CR compared with those with grade III–IV, probably because it is easier to reach complete response when the disease is in a less advanced stage at the onset of the MSC treatment. Since 83% of the included patients presented grade III–IV, it is not possible to draw a firm conclusion regarding the correlation with aGvHD severity. In order to prove that the efficacy of MSC therapy is higher in patients with mild or moderate aGvHD, it would be necessary to carry out a clinical trial including patients with steroid-refractory aGvHD grade I–II.

We included in our meta-analysis a phase 3 randomized trial [43] of 260 patients (MSC = 163 patients, control = 81 patients) who did not receive other treatments than steroids before the randomization. This trial showed no differences for survival at day 180, and the OR was significantly higher for the MSC group in pediatric patients, patients with high-risk aGvHD and liver involvements. However, MSC and control groups were imbalanced for organ involvement, GvHD grade, patient category, and proportion of patients that did not receive any additional second-line therapy. In contrast, in our meta-analysis, MSC infusion was associated with a higher improvement in OR for the skin compared with liver or gut involvement.

Between 30% and 50% of patients undergoing HSCT develop cGVHD [7, 44], of which approximately 30% is de novo [7]. Our meta-analysis showed that 64% patients infused with MSC for the treatment of cGvHD survived at last follow-up. The lack of control patients in most studies makes it difficult to interpret the potential effects of MSC therapy. In addition to those previously discussed, other factors could participate in the outcome of patients with acute or chronic manifestations, such as prior and concomitant GvHD therapy, which varied greatly across studies.

## Conclusion

Our meta-analysis indicates that allogeneic MSC could be useful to prevent and treat GvHD in patients undergoing allogeneic HSCT. However, the great variability across studies and between patients in individual studies makes it necessary to carry out a large multi-center clinical trial with uniform criteria regarding GvHD prophylaxis, GvHD treatment lines, and conditioning regimen. The inclusion of equivalent populations of patients in the control and MSC groups is critical to determine the real effect of the MSC infusion in the outcome of the patients. Based on this meta-analysis, our recommendation would be to administer MSC at day 0 in patients undergoing HSCT and to carry out a clinical trial using MSC as an adjuvant therapy from disease onset. It will be required to establish well-defined end points, MSC dose stratification, and defined frequencies of administration to help clinicians to elaborate an optimal protocol for the treatment of GvHD with MSC that improves the dismal prognosis of these patients.

## Supplementary information

**Additional file 1: Figure S1.** Risk of Bias. **Figure S2.** Overall survival of MSC and control patients. **Figure S3.** Overall survival at last follow-up. **Figure S4.** aGvHD incidence for patients from the MSC group compared to control patients. **Figure S5.** aGvHD (grade III-IV) incidence for patients from the MSC group compared to control patients. **Figure S6.** Outcome and MSC doses of patients infused for prophylaxis. **Figure S7.** Risk of Bias. **Figure S8.** Overall survival of patients with aGvHD at last follow-up. **Figure S9.** Grade-specific overall survival of patients with aGvHD from the MSC group at last follow-up. **Figure S10.** Overall survival of patients with aGvHD from the MSC group regarding to the number of organ affected. **Figure S11.** Organ-specific overall survival of patients with aGvHD from the MSC group at last follow-up. **Figure S12.** Overall survival of aGvHD patients with multiorgan affection from the MSC group at last follow-up. **Figure S13.** Overall survival and first day of infusion of MSC from HSCT of patients with aGvHD. **Figure S14.** Overall and complete responses of patients with aGvHD from the MSC group. **Figure S15.** Grade-specific overall response of patients with aGvHD from the MSC group at last follow-up. **Figure S16.** Overall response of patients with aGvHD from the MSC group regarding to the number of organs affected. **Figure S17.** Complete response of patients with aGvHD from the MSC group regarding to the number of organ affected. **Figure S18.** Complete response of patients with aGvHD grade II vs grade III-IV. **Figure S19.** Organ-specific overall and complete responses of patients with aGvHD from the MSC group. **Figure S20.** Gut-specific overall response of patients with aGvHD from the MSC group. **Figure S21.** Correlation between responder rates and MSC dose or time from HSCT. **Figure S22.** Outome of aGvHD vs cGvHD patients. **Table S1.** Detailed search strategy. **Table S2.** Characteristics of the included studies that evaluated the use of MSC for GvHD prophylaxis. **Table S3.** Characteristics of the included studies using MSC for the treatment of aGvHD and/or cGvHD. **Table S4.** Characteristics of the control group for included studies using MSC for the treatment of GvHD. **Table S5.** Number of patients with aGvHD infused with MSC divided by grade and organs involved. **Table S6.** Subgroup analysis for the overall survival of patients with aGvHD infused with MSC at last follow-up. **Table S7.** Subgroup analysis for the overall response of patients with aGvHD infused with MSC. **Table S8.** Subgroup analysis for the complete response of patients with aGvHD infused with MSC.

## Data Availability

All supporting data are included in the article and Additional file.

## Abbreviations

AD: Adipose tissue
aGvHD: Acute graft-versus-host disease
BM: Bone marrow BM
cGvHD: Chronic graft-versus-host disease
CI: Confidence interval
CR: Complete response
CsA: Cyclosporin A
GvHD: Graft-versus-host disease
HLAs: Human leucocyte antigens
HSCT: Hematopoietic stem cell transplantation
MSC: Mesenchymal stromal cells
MTX: Methotrexate
OR: Overall response
OS: Overall survival
PR: Partial response
RCTs: Randomized controlled trials
RD: Risk differences
RoBANS: Risk of bias assessment tool for non-randomized studies
RR: Risk ratio
UC: Umbilical cord
