# Supplementary material for: Mesenchymal stromal cells for the prophylaxis and treatment of graft-versus-host disease—a meta-analysis
Source: Stem Cell Res Ther. 2020 Feb 18;11:64. doi: 10.1186/s13287-020-01592-z (PMC7027118; doi:10.1186/s13287-020-01592-z)
Supplement: Supplementary file 1 — Additional file 1: Figure S1. Risk of Bias. Figure S2. Overall survival of MSC and control patients. Figure S3. Overall survival at last follow-up. Figure S4. aGvHD incidence for patients from the MSC group compared to control patients. Figure S5. aGvHD (grade III-IV) incidence for patients from the MSC group compared to control patients. Figure S6. Outcome and MSC doses of patients infused for prophylaxis. Figure S7. Risk of Bias. Figure S8. Overall survival of patients with aGvHD at last follow-up. Figure S9. Grade-specific overall survival of patients with aGvHD from the MSC group at last follow-up. Figure S10. Overall survival of patients with aGvHD from the MSC group regarding to the number of organ affected. Figure S11. Organ-specific overall survival of patients with aGvHD from the MSC group at last follow-up. Figure S12. Overall survival of aGvHD patients with multiorgan affection from the MSC group at last follow-up. Figure S13. Overall survival and first day of infusion of MSC from HSCT of patients with aGvHD. Figure S14. Overall and complete responses of patients with aGvHD from the MSC group. Figure S15. Grade-specific overall response of patients with aGvHD from the MSC group at last follow-up. Figure S16. Overall response of patients with aGvHD from the MSC group regarding to the number of organs affected. Figure S17. Complete response of patients with aGvHD from the MSC group regarding to the number of organ affected. Figure S18. Complete response of patients with aGvHD grade II vs grade III-IV. Figure S19. Organ-specific overall and complete responses of patients with aGvHD from the MSC group. Figure S20. Gut-specific overall response of patients with aGvHD from the MSC group. Figure S21. Correlation between responder rates and MSC dose or time from HSCT. Figure S22. Outome of aGvHD vs cGvHD patients. Table S1. Detailed search strategy. Table S2. Characteristics of the included studies that evaluated the use of MSC for GvHD prophylaxis. Table S3. Cha [file 13287_2020_1592_MOESM1_ESM.pdf]

Supplementary Appendix

Mesenchymal stromal cells for the prophylaxis and treatment of graft-versus-host disease – A meta-analysis.

Table of Contents:

Supplementary Figures. 3
Supplementary Tables. 28
References. 44

Supplementary Figures.

Figure S1. Risk of Bias.

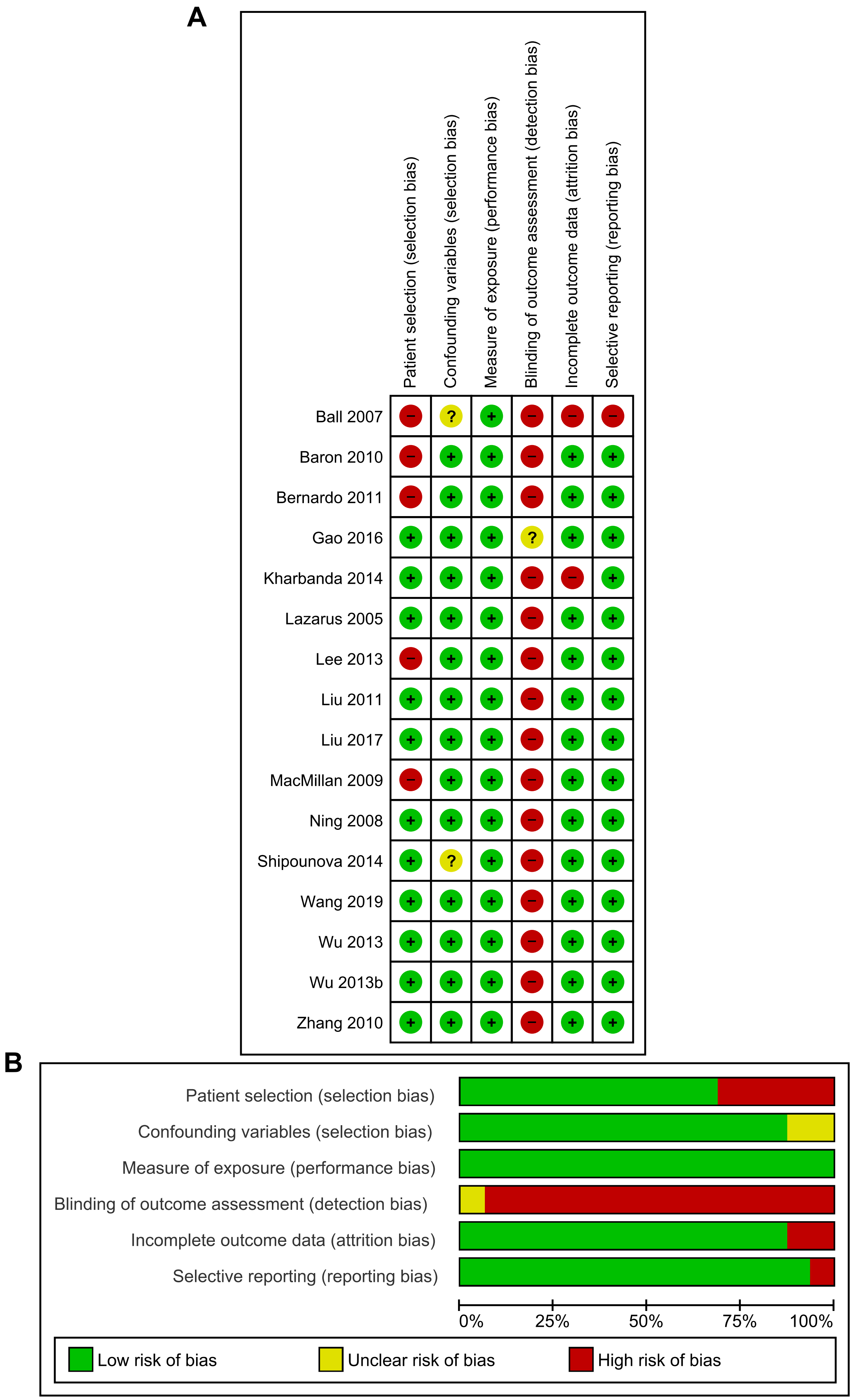

(A) Risk of bias summary: review authors' judgements about each risk of bias item for each included prophylaxis-related studies. (B) Risk of bias graph: review authors' judgements about each risk of bias item presente as percentages across all included prophylaxis-related studies.

Figure S2. Overall survival of MSC and control patients.

A GvHD prophylaxis – overall survival: MSC group

Study or Subgroup	Risk Difference	SE	Surviving patients	Total patients	Weight	Risk Difference IV, Random, 95% CI
**Children**						
Ball 2007	0.71	0.1173	10	14	5.0%	0.71 [0.48, 0.94]
Bernardo 2011	0.77	0.1173	10	13	5.0%	0.77 [0.54, 1.00]
Kharbanda 2014	0.33	0.1888	2	6	2.4%	0.33 [-0.04, 0.70]
Lee 2013	0.9	0.0969	6	7	6.5%	0.90 [0.71, 1.09]
MacMillan 2009	0.63	0.1735	5	8	2.7%	0.63 [0.29, 0.97]
Wang 2019	0.86	0.0612	30	35	10.3%	0.86 [0.74, 0.98]
Wu 2013	0.8	0.1786	4	5	2.6%	0.80 [0.45, 1.15]
Wu 2013b	0.75	0.1531	6	8	3.4%	0.75 [0.45, 1.05]
**Subtotal (95% CI)**			**73**	**96**	**37.9%**	**0.77 [0.67, 0.87]**
Heterogeneity: Tau² = 0.01; Chi² = 9.93, df = 7 (P = 0.19); I² = 30%						
Test for overall effect: Z = 15.14 (P < 0.00001)						
**Adults**						
Baron 2010	0.8	0.0918	16	20	6.9%	0.80 [0.62, 0.98]
Lazarus 2005	0.78	0.0612	36	46	10.3%	0.78 [0.66, 0.90]
Ning 2008	0.3	0.1429	3	10	3.8%	0.30 [0.02, 0.58]
Shipounova 2014	0.77	0.0663	30	39	9.6%	0.77 [0.64, 0.90]
Zhang 2010	0.58	0.1429	7	12	3.8%	0.58 [0.30, 0.86]
**Subtotal (95% CI)**			**92**	**127**	**34.3%**	**0.69 [0.55, 0.83]**
Heterogeneity: Tau² = 0.02; Chi² = 11.76, df = 4 (P = 0.02); I² = 66%						
Test for overall effect: Z = 9.78 (P < 0.00001)						
**Children and adults**						
Gao 2016	0.66	0.0612	41	62	10.3%	0.66 [0.54, 0.78]
Liu 2011	0.7	0.0867	19	27	7.4%	0.70 [0.53, 0.87]
Liu 2017	0.77	0.0612	34	44	10.3%	0.77 [0.65, 0.89]
**Subtotal (95% CI)**			**94**	**133**	**27.9%**	**0.71 [0.64, 0.79]**
Heterogeneity: Tau² = 0.00; Chi² = 1.64, df = 2 (P = 0.44); I² = 0%						
Test for overall effect: Z = 18.39 (P < 0.00001)						
**Total (95% CI)**			**259**	**356**	**100.0%**	**0.73 [0.67, 0.79]**
Heterogeneity: Tau² = 0.01; Chi² = 25.81, df = 15 (P = 0.04); I² = 42%						
Test for overall effect: Z = 23.30 (P < 0.00001)						
Test for subgroup differences: Chi² = 1.22, df = 2 (P = 0.54), I² = 0%						

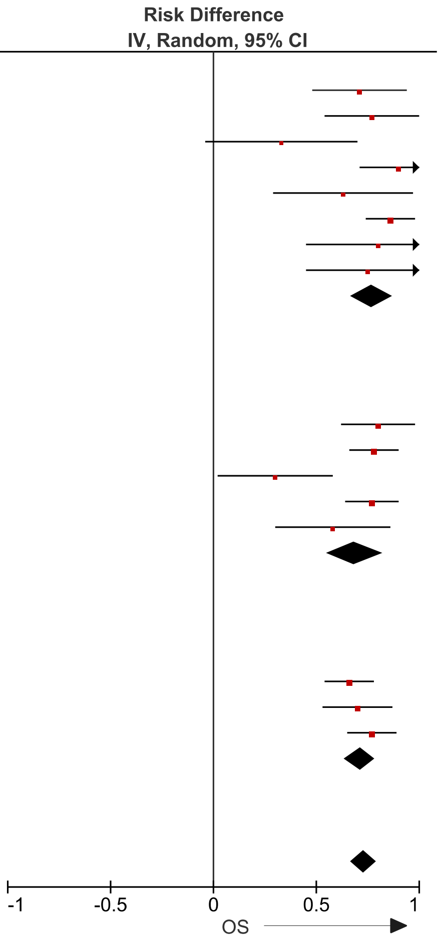

B GvHD prophylaxis – overall survival: control group

Study or Subgroup	Risk Difference	SE	Surviving patients	Total patients	Weight	Risk Difference IV, Random, 95% CI
**Children**						
Ball 2007	0.77	0.0663	36	47	14.7%	0.77 [0.64, 0.90]
Bernardo 2011	0.64	0.0765	25	39	12.7%	0.64 [0.49, 0.79]
Lee 2013	0.56	0.1684	5	9	4.1%	0.56 [0.23, 0.89]
MacMillan 2009	0.46	0.102	11	23	8.9%	0.46 [0.26, 0.66]
Wu 2013	0.56	0.1684	5	9	4.1%	0.56 [0.23, 0.89]
Wu 2013b	0.67	0.1378	8	12	5.7%	0.67 [0.40, 0.94]
**Subtotal (95% CI)**			**90**	**139**	**50.0%**	**0.64 [0.53, 0.74]**
Heterogeneity: Tau² = 0.01; Chi² = 7.35, df = 5 (P = 0.20); I² = 32%						
Test for overall effect: Z = 12.12 (P < 0.00001)						
**Adults**						
Ning 2008	0.33	0.1638	5	15	4.3%	0.33 [0.01, 0.65]
Shipounova 2014	0.58	0.0816	22	38	11.8%	0.58 [0.42, 0.74]
**Subtotal (95% CI)**			**27**	**53**	**16.0%**	**0.50 [0.26, 0.73]**
Heterogeneity: Tau² = 0.01; Chi² = 1.87, df = 1 (P = 0.17); I² = 46%						
Test for overall effect: Z = 4.19 (P < 0.0001)						
**Children and adults**						
Baron 2010	0.44	0.1276	7	16	6.4%	0.44 [0.19, 0.69]
Gao 2016	0.64	0.0612	38	62	15.8%	0.64 [0.52, 0.76]
Liu 2011	0.5	0.0816	19	28	11.8%	0.50 [0.34, 0.66]
**Subtotal (95% CI)**			**64**	**106**	**34.0%**	**0.56 [0.44, 0.67]**
Heterogeneity: Tau² = 0.00; Chi² = 3.08, df = 2 (P = 0.21); I² = 35%						
Test for overall effect: Z = 9.20 (P < 0.00001)						
**Total (95% CI)**			**181**	**298**	**100.0%**	**0.59 [0.52, 0.66]**
Heterogeneity: Tau² = 0.00; Chi² = 15.36, df = 10 (P = 0.12); I² = 35%						
Test for overall effect: Z = 16.03 (P < 0.00001)						
Test for subgroup differences: Chi² = 1.73, df = 2 (P = 0.42), I² = 0%						

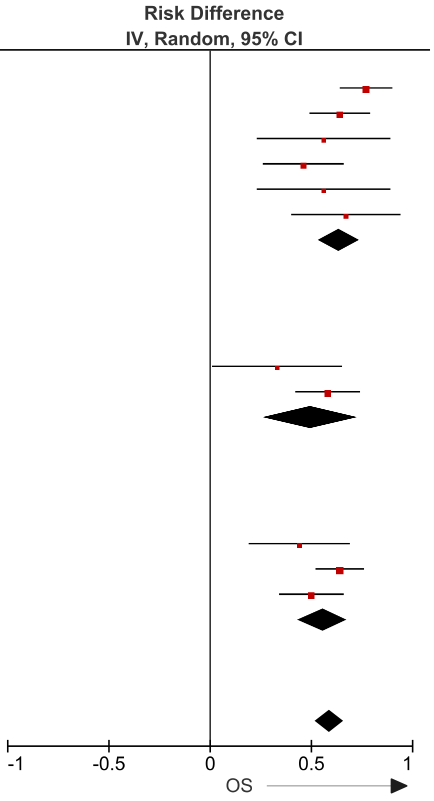

Forest-plots of overall survival at last follow-up of patients undergoing HSCT and infused with MSC (A) and control patients (B). Dots and black lines represent the effect and 95% CI of individual studies. Black diamonds represent the overall effect size. Weight are random-effects analysis.

Figure S3. Overall survival at last follow-up.

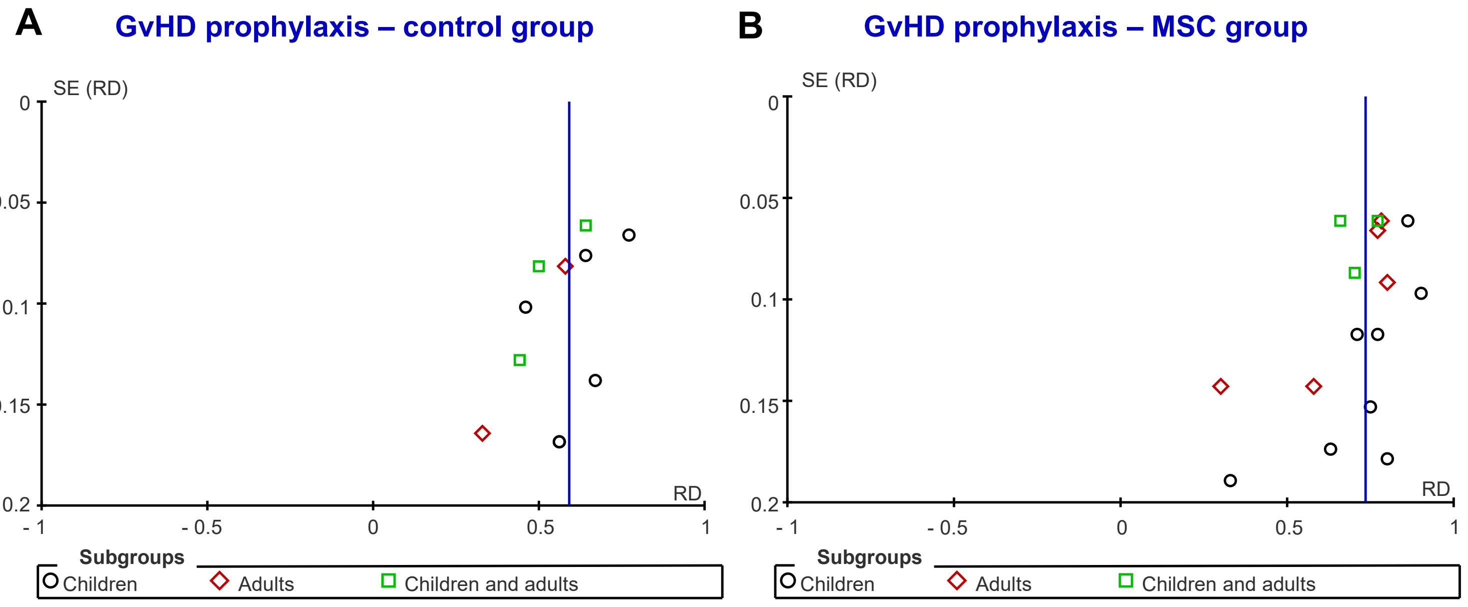

Funnel-plots of overall survival from the control (A) and MSC (B) groups. The blue line represents the overall effect size. The horizontal and vertical axes represent the risk difference and the standard error, respectively. Circles, diamonds and squares represent data from individual studies for children, adults and both children and adults, respectively.

Figure S4. aGvHD incidence for patients from the MSC group compared to control patients.

Study or Subgroup	MSC Events	MSC Total	Control Events	Control Total	Weight	Risk Ratio M-H, Random, 95% CI
**Children**						
Ball 2007	2	14	14	47	3.2%	0.48 [0.12, 1.86]
Bernardo 2011	5	13	21	39	10.5%	0.71 [0.34, 1.51]
Lee 2013	5	7	5	8	11.5%	1.14 [0.56, 2.33]
MacMillan 2009	3	8	5	23	4.2%	1.73 [0.53, 5.64]
Wu 2013	2	5	6	9	4.3%	0.60 [0.19, 1.93]
Wu 2013b	4	8	8	12	9.1%	0.75 [0.34, 1.67]
**Subtotal (95% CI)**		**55**		**138**	**42.9%**	**0.85 [0.59, 1.23]**
Total events	21		59			
Heterogeneity: Tau² = 0.00; Chi² = 3.49, df = 5 (P = 0.62); I² = 0%						
Test for overall effect: Z = 0.85 (P = 0.39)						
**Adults**						
Ning 2008	4	9	11	15	9.3%	0.61 [0.27, 1.34]
Shipounova 2014	4	39	8	38	4.7%	0.49 [0.16, 1.48]
**Subtotal (95% CI)**		**48**		**53**	**14.1%**	**0.56 [0.30, 1.07]**
Total events	8		19			
Heterogeneity: Tau² = 0.00; Chi² = 0.11, df = 1 (P = 0.74); I² = 0%						
Test for overall effect: Z = 1.74 (P = 0.08)						
**Children and adults**						
Baron 2010	9	20	9	16	13.9%	0.80 [0.42, 1.53]
Liu 2011	16	27	16	28	29.2%	1.04 [0.66, 1.62]
**Subtotal (95% CI)**		**47**		**44**	**43.1%**	**0.95 [0.66, 1.38]**
Total events	25		25			
Heterogeneity: Tau² = 0.00; Chi² = 0.42, df = 1 (P = 0.52); I² = 0%						
Test for overall effect: Z = 0.25 (P = 0.80)						
**Total (95% CI)**		**150**		**235**	**100.0%**	**0.84 [0.66, 1.07]**
Total events	54		103			
Heterogeneity: Tau² = 0.00; Chi² = 6.07, df = 9 (P = 0.73); I² = 0%						
Test for overall effect: Z = 1.38 (P = 0.17)						
Test for subgroup differences: Chi² = 1.93, df = 2 (P = 0.38), I² = 0%						

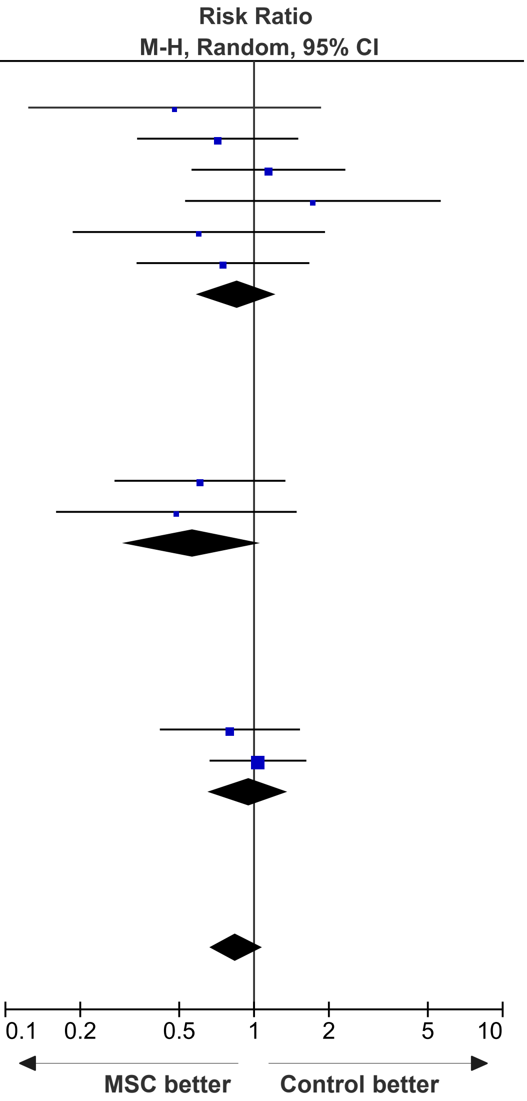

Forest-plot showing the risk to suffer GvHD for patients from the MSC group compared with control patients. Dots and black lines represent the effect and 95% CI of individual studies. Black diamonds represent the overall effect size. Weight are from random-effects analysis.

Figure S5. aGvHD (grade III-IV) incidence for patients from the MSC group compared to control patients.

Study or Subgroup	MSC Events	MSC Total	Control Events	Control Total	Weight	Risk Ratio M-H, Random, 95% CI
**Children**						
Ball 2007	0	14	2	47	11.0%	0.64 [0.03, 12.61]
Bernardo 2011	0	13	10	39	12.7%	0.14 [0.01, 2.17]
Lee 2013	1	7	0	8	10.5%	3.38 [0.16, 71.67]
Wu 2013	0	5	1	9	10.6%	0.56 [0.03, 11.57]
Wu 2013b	0	8	1	12	10.2%	0.48 [0.02, 10.54]
**Subtotal (95% CI)**		**47**		**115**	**55.0%**	**0.57 [0.15, 2.15]**
Total events	1		14			
Heterogeneity: Tau² = 0.00; Chi² = 2.45, df = 4 (P = 0.65); I² = 0%						
Test for overall effect: Z = 0.84 (P = 0.40)						
**Adults**						
Baron 2010	2	20	3	16	35.2%	0.53 [0.10, 2.82]
**Subtotal (95% CI)**		**20**		**16**	**35.2%**	**0.53 [0.10, 2.82]**
Total events	2		3			
Heterogeneity: Not applicable						
Test for overall effect: Z = 0.74 (P = 0.46)						
**Children and adults**						
Liu 2011	0	27	1	28	9.8%	0.35 [0.01, 8.12]
**Subtotal (95% CI)**		**27**		**28**	**9.8%**	**0.35 [0.01, 8.12]**
Total events	0		1			
Heterogeneity: Not applicable						
Test for overall effect: Z = 0.66 (P = 0.51)						
**Total (95% CI)**		**94**		**159**	**100.0%**	**0.53 [0.20, 1.42]**
Total events	3		18			
Heterogeneity: Tau² = 0.00; Chi² = 2.50, df = 6 (P = 0.87); I² = 0%						
Test for overall effect: Z = 1.27 (P = 0.21)						
Test for subgroup differences: Chi² = 0.08, df = 2 (P = 0.96), I² = 0%						

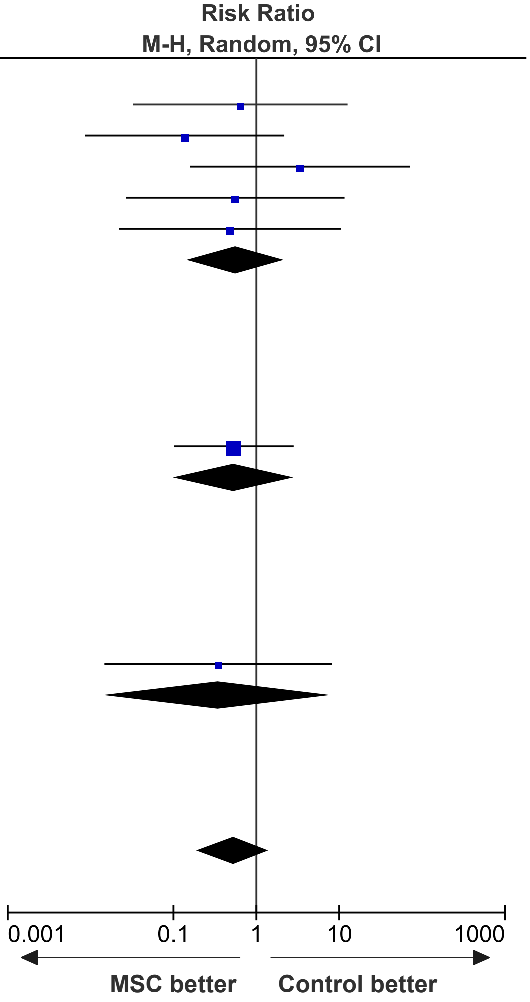

Forest-plot showing the risk to suffer aGvHD grade III-IV in patients from the MSC group compared with the control group. Dots and black lines represent the effect and 95% CI of individual studies. Black diamonds represent the overall effect size. Weight are from random-effects analysis.

Figure S6. Outcome and MSC doses of patients infused **for prophylaxis**.

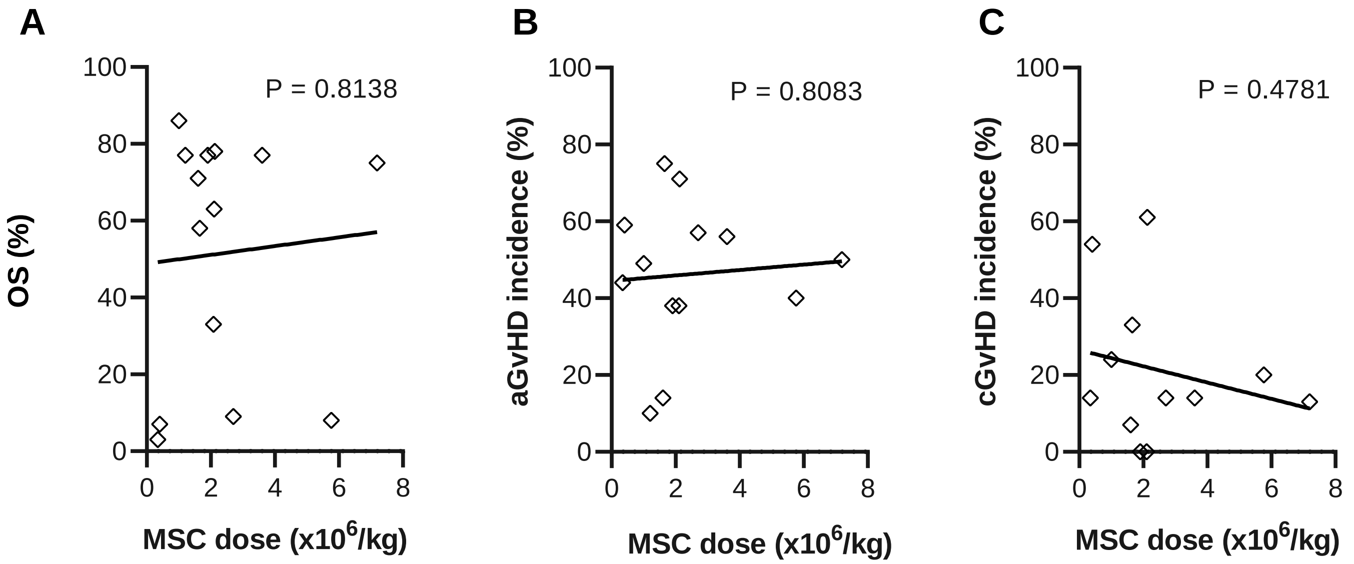

Correlation between MSC doses and overall survival (A), aGvHD (B) and cGvHD (C) incidence at last follow-up of patients undergoing administration of MSC and HSC. Diamonds represent individual studies.

Figure S7. Risk of Bias.

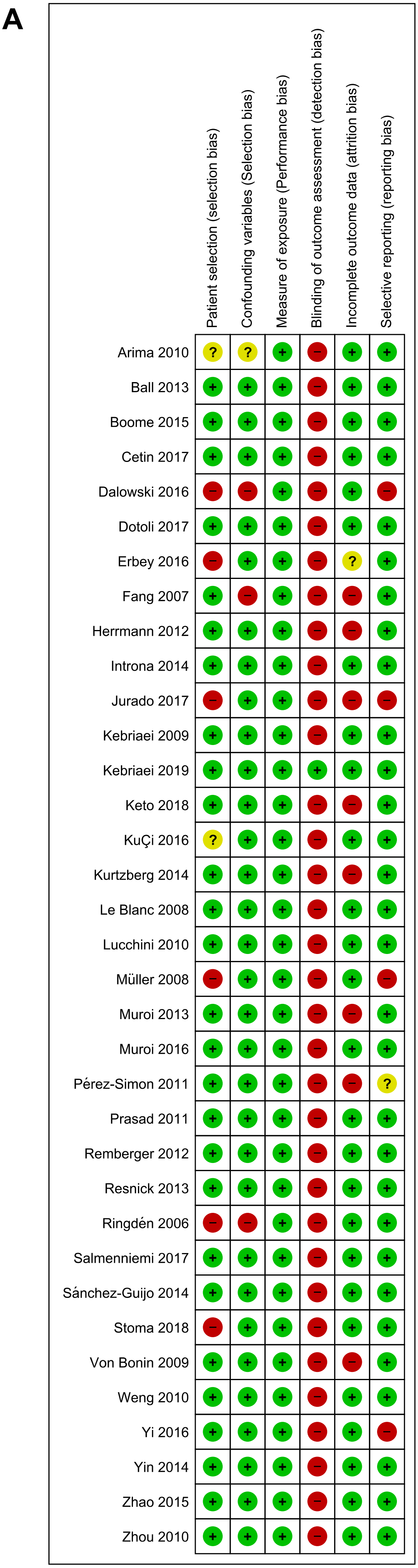

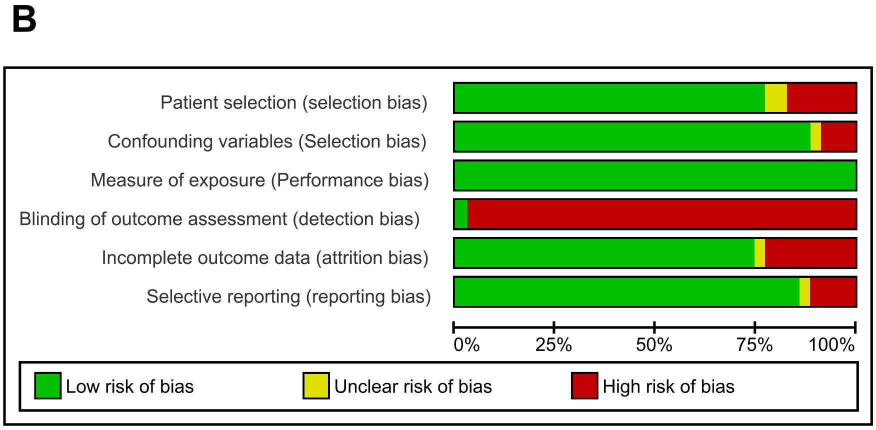

(A) Risk of bias summary: review authors' judgements about each risk of bias item for each included study related to the use of MSC for the treatment of GvHD. (B) Risk of bias graph: review authors' judgements about each risk of bias item presented as percentage across all included studies related to the use of MSC for the treatment of GvHD.

Figure S8. Overall survival of patients with aGvHD at last follow-up.

A aGvHD treatment – overall survival: control group

Study or Subgroup	Risk Difference	SE	Surviving Patients	Total Patients	Weight	Risk Difference IV, Random, 95% CI
Dalowski 2016	0.09	0.051	3	35	22.6%	0.09 [-0.01, 0.19]
Kebriaei 2019	0.42	0.0561	34	81	22.0%	0.42 [0.31, 0.53]
Remberger 2012	0.15	0.0969	2	13	17.5%	0.15 [-0.04, 0.34]
Stoma 2018	0.33	0.0714	13	34	20.4%	0.33 [0.19, 0.47]
Zhao 2015	0.26	0.0969	5	19	17.5%	0.26 [0.07, 0.45]
**Total (95% CI)**			**57**	**182**	**100.0%**	**0.25 [0.11, 0.39]**

Heterogeneity: Tau² = 0.02; Chi² = 21.35, df = 4 (P = 0.0003); I² = 81%
Test for overall effect: Z = 3.46 (P = 0.0005)

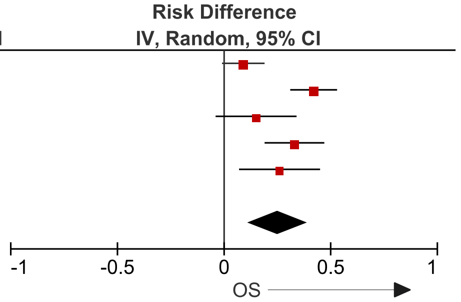

B aGvHD treatment – overall survival: MSC vs control groups

Study or Subgroup	MSC Events	MSC Total	Control Events	Control Total	Weight	Risk Ratio M-H, Random, 95% CI
Dalowski 2016	11	58	3	35	10.9%	2.21 [0.66, 7.39]
Kebriaei 2019	56	163	34	81	35.9%	0.82 [0.59, 1.14]
Remberger 2012	3	15	2	13	6.7%	1.30 [0.26, 6.62]
Stoma 2018	20	34	13	34	28.5%	1.54 [0.92, 2.57]
Zhao 2015	15	28	5	19	18.1%	2.04 [0.89, 4.66]
**Total (95% CI)**		**298**		**182**	**100.0%**	**1.33 [0.84, 2.10]**
Total events	105		57			

Heterogeneity: Tau² = 0.12; Chi² = 8.22, df = 4 (P = 0.08); I² = 51%
Test for overall effect: Z = 1.21 (P = 0.22)

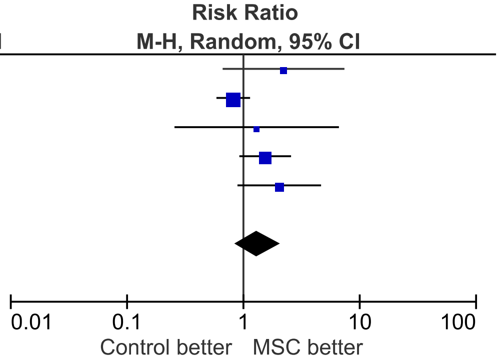

Forest-plots of the overall survival of patients with aGvHD at last follow-up. (A) Overall survival of patients from the control group. (B) Overall survival of patients infused with MSC compared with the control group. Dots and black lines represent the effect and 95% CI of individual studies. Black diamonds represent the overall effect size. Weight are from random-effects analysis.

Figure S9. Grade-specific overall survival of patients with aGvHD from the MSC group at last follow-up.

Study or Subgroup	Risk Difference	SE	Surviving patients	Total patients	Weight	Risk Difference IV, Random, 95% CI
**Grade II**						
Herrmann 2012	1	0.5	1	1	0.9%	1.00 [0.02, 1.98]
KuÇi 2016	0	0.5	0	1	0.9%	0.00 [-0.98, 0.98]
Muroi 2013	0.56	0.1684	5	9	6.0%	0.56 [0.23, 0.89]
Müller 2007	0	0.5	0	1	0.9%	0.00 [-0.98, 0.98]
Pérez-Simon 2011	0	0.5	0	2	0.9%	0.00 [-0.98, 0.98]
Ringdén 2006	0	0.5	0	1	0.9%	0.00 [-0.98, 0.98]
Sánchez-Guijo 2014	0.71	0.1684	5	7	6.0%	0.71 [0.38, 1.04]
**Subtotal (95% CI)**			**11**	**22**	**16.7%**	**0.52 [0.29, 0.75]**
Heterogeneity: Tau² = 0.01; Chi² = 6.55, df = 6 (P = 0.36); I² = 8%						
Test for overall effect: Z = 4.45 (P < 0.00001)						
**Grade III**						
Fang 2007	0.5	0.352	1	2	1.8%	0.50 [-0.19, 1.19]
Herrmann 2012	0.5	0.2041	3	6	4.5%	0.50 [0.10, 0.90]
KuÇi 2016	0.91	0.0867	10	11	12.3%	0.91 [0.74, 1.08]
Muroi 2013	0.6	0.2194	3	5	4.1%	0.60 [0.17, 1.03]
Muroi 2016	0.52	0.1071	11	21	10.3%	0.52 [0.31, 0.73]
Müller 2008	1	0.5	1	1	0.9%	1.00 [0.02, 1.98]
Prasad 2011	0.63	0.1735	2	5	5.8%	0.63 [0.29, 0.97]
Pérez-Simon 2011	0.33	0.2704	1	3	2.9%	0.33 [-0.20, 0.86]
Ringdén 2006	0.8	0.1786	4	5	5.5%	0.80 [0.45, 1.15]
Sánchez-Guijo 2014	0.43	0.1327	6	14	8.2%	0.43 [0.17, 0.69]
**Subtotal (95% CI)**			**42**	**73**	**56.2%**	**0.62 [0.48, 0.77]**
Heterogeneity: Tau² = 0.02; Chi² = 16.51, df = 9 (P = 0.06); I² = 45%						
Test for overall effect: Z = 8.29 (P < 0.00001)						
**Grade IV**						
Fang 2007	0.75	0.2143	3	4	4.2%	0.75 [0.33, 1.17]
Herrmann 2012	0.5	0.25	2	4	3.3%	0.50 [0.01, 0.99]
KuÇi 2016	0.69	0.1173	11	16	9.4%	0.69 [0.46, 0.92]
Muroi 2016	0.33	0.2704	1	3	2.9%	0.33 [-0.20, 0.86]
Prasad 2011	0.5	0.2041	3	6	4.5%	0.50 [0.10, 0.90]
Pérez-Simon 2011	0	0.5	0	4	0.9%	0.00 [-0.98, 0.98]
Ringdén 2006	0	0.5	0	1	0.9%	0.00 [-0.98, 0.98]
Sánchez-Guijo 2014	0	0.5	0	4	0.9%	0.00 [-0.98, 0.98]
**Subtotal (95% CI)**			**20**	**42**	**27.1%**	**0.57 [0.41, 0.72]**
Heterogeneity: Tau² = 0.00; Chi² = 6.63, df = 7 (P = 0.47); I² = 0%						
Test for overall effect: Z = 7.19 (P < 0.00001)						
**Total (95% CI)**			**73**	**137**	**100.0%**	**0.59 [0.49, 0.68]**
Heterogeneity: Tau² = 0.01; Chi² = 31.52, df = 24 (P = 0.14); I² = 24%						
Test for overall effect: Z = 11.82 (P < 0.00001)						
Test for subgroup differences: Chi² = 0.63, df = 2 (P = 0.73), I² = 0%						

Risk Difference
IV, Random, 95% CI

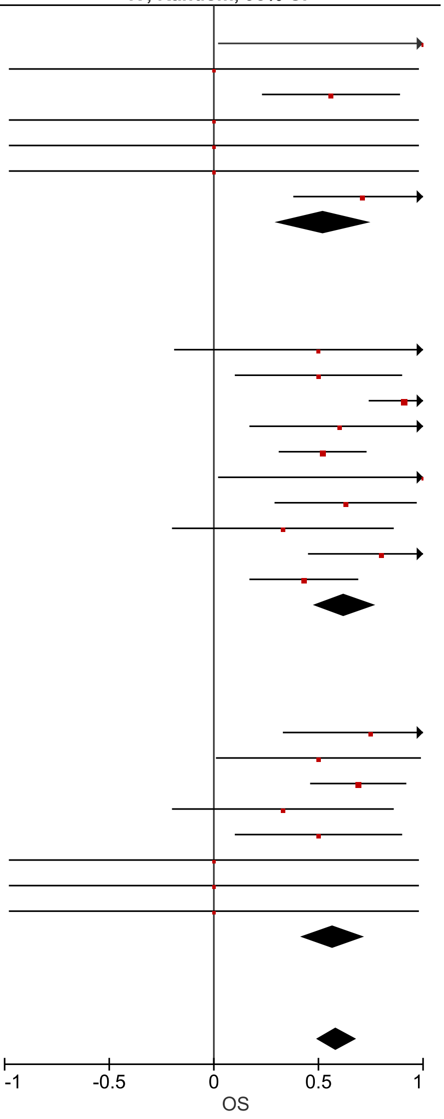

Forest-plot of grade-specific overall survival of patients with aGvHD from the MSC group at last follow-up. Dots and black lines represent the effect and 95% CI of individual studies. Black diamonds represent the overall effect size. Weight are from random-effects analysis.

Figure S10. Overall survival of patients with aGvHD from the MSC group regarding to the number of organ affected.

Study or Subgroup	Risk Difference	SE	Surviving patients	Total patients	Weight	Risk Difference IV, Random, 95% CI
**1 organ**						
Arima 2010	0	0.5	0	1	0.4%	0.00 [-0.98, 0.98]
Erbey 2016	1	0.5	5	5	0.4%	1.00 [0.02, 1.98]
Fang 2007	1	0.5	1	1	0.4%	1.00 [0.02, 1.98]
Herrmann 2012	1	0.5	1	1	0.4%	1.00 [0.02, 1.98]
KuÇi 2016	0.78	0.51	7	9	0.4%	0.78 [-0.22, 1.78]
Lucchini 2010	0.83	0.148	5	6	4.8%	0.83 [0.54, 1.12]
Muroi 2013	0.67	0.1939	4	6	2.8%	0.67 [0.29, 1.05]
Muroi 2016	0.5	0.1429	6	12	5.2%	0.50 [0.22, 0.78]
Prasad 2011	0.4	0.1888	2	5	3.0%	0.40 [0.03, 0.77]
Pérez-Simon 2011	0.17	0.1531	1	6	4.5%	0.17 [-0.13, 0.47]
Ringdén 2006	0.67	0.2755	2	3	1.4%	0.67 [0.13, 1.21]
Sánchez-Guijo 2014	0.6	0.1531	6	10	4.5%	0.60 [0.30, 0.90]
**Subtotal (95% CI)**			**40**	**65**	**28.4%**	**0.56 [0.41, 0.71]**
Heterogeneity: Tau² = 0.02; Chi² = 15.01, df = 11 (P = 0.18); I² = 27%						
Test for overall effect: Z = 7.28 (P < 0.00001)						
**2 organs**						
Arima 2010	0	0.5	0	1	0.4%	0.00 [-0.98, 0.98]
Erbey 2016	0.64	0.1276	9	14	6.5%	0.64 [0.39, 0.89]
Fang 2007	0.5	0.25	2	4	1.7%	0.50 [0.01, 0.99]
Herrmann 2012	0.5	0.2041	3	6	2.5%	0.50 [0.10, 0.90]
KuÇi 2016	0.56	0.1684	5	9	3.7%	0.56 [0.23, 0.89]
Lucchini 2010	0	0.5	0	2	0.4%	0.00 [-0.98, 0.98]
Muroi 2013	0.43	0.06	3	7	29.4%	0.43 [0.31, 0.55]
Muroi 2016	0.45	0.148	5	11	4.8%	0.45 [0.16, 0.74]
Müller 2008	0	0.5	0	1	0.4%	0.00 [-0.98, 0.98]
Prasad 2011	0.6	0.2194	3	5	2.2%	0.60 [0.17, 1.03]
Pérez-Simon 2011	0	0.5	0	2	0.4%	0.00 [-0.98, 0.98]
Ringdén 2006	0.6	0.2194	3	5	2.2%	0.60 [0.17, 1.03]
Sánchez-Guijo 2014	0.36	0.1429	4	11	5.2%	0.36 [0.08, 0.64]
**Subtotal (95% CI)**			**37**	**78**	**60.0%**	**0.46 [0.38, 0.54]**
Heterogeneity: Tau² = 0.00; Chi² = 7.35, df = 12 (P = 0.83); I² = 0%						
Test for overall effect: Z = 10.99 (P < 0.00001)						
**3 organs**						
Arima 2010	0	0.5	0	1	0.4%	0.00 [-0.98, 0.98]
Erbey 2016	0.36	0.1276	5	14	6.5%	0.36 [0.11, 0.61]
Fang 2007	1	0.5	1	1	0.4%	1.00 [0.02, 1.98]
Herrmann 2012	1	0.5	2	2	0.4%	1.00 [0.02, 1.98]
KuÇi 2016	1	0.5	8	8	0.4%	1.00 [0.02, 1.98]
Muroi 2016	1	0.5	1	1	0.4%	1.00 [0.02, 1.98]
Müller 2008	1	0.5	1	1	0.4%	1.00 [0.02, 1.98]
Prasad 2011	0	0.5	0	2	0.4%	0.00 [-0.98, 0.98]
Pérez-Simon 2011	0	0.5	0	2	0.4%	0.00 [-0.98, 0.98]
Sánchez-Guijo 2014	0.5	0.25	2	4	1.7%	0.50 [0.01, 0.99]
**Subtotal (95% CI)**			**20**	**36**	**11.6%**	**0.46 [0.27, 0.65]**
Heterogeneity: Tau² = 0.00; Chi² = 9.01, df = 9 (P = 0.44); I² = 0%						
Test for overall effect: Z = 4.78 (P < 0.00001)						
**Total (95% CI)**			**97**	**179**	**100.0%**	**0.49 [0.42, 0.55]**
Heterogeneity: Tau² = 0.00; Chi² = 33.01, df = 34 (P = 0.52); I² = 0%						
Test for overall effect: Z = 14.97 (P < 0.00001)						
Test for subgroup differences: Chi² = 1.39, df = 2 (P = 0.50), I² = 0%						

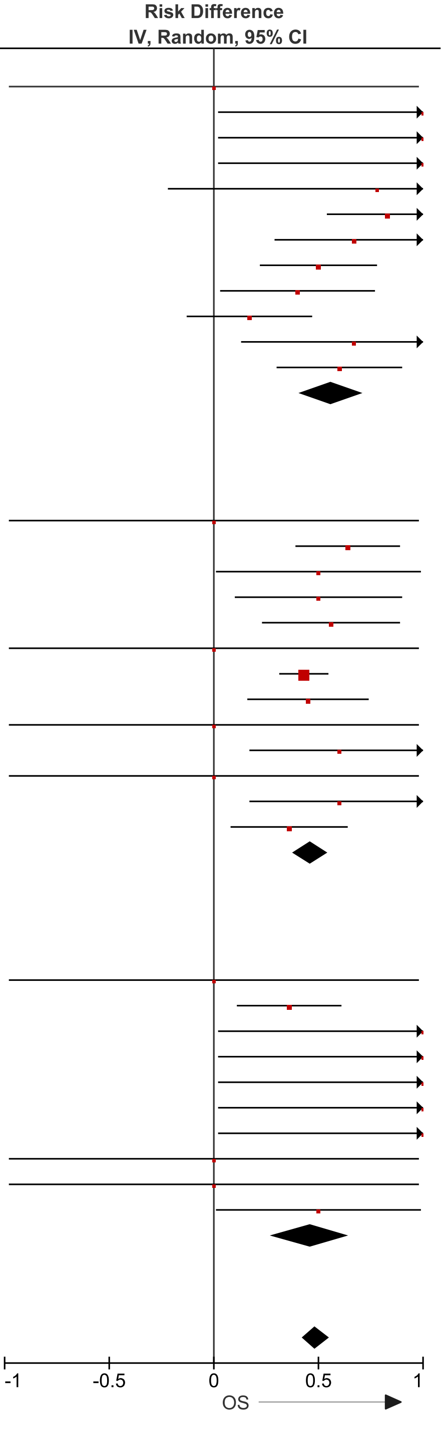

Forest-plot showing the overall survival of patients with aGvHD from the MSC group at last follow-up regarding to the number of organs affected. Dots and black lines represent the effect and 95% CI of individual studies. Black diamonds represent the overall effect size. Weight are from random-effects analysis.

Figure S11. Organ-specific overall survival of patients with aGvHD from the MSC group at last follow-up.

Study or Subgroup	Risk Difference	SE	Surviving patients	Total patients	Weight	Risk Difference IV, Random, 95% CI
**Skin**						
Arima 2010	0	0.5	0	2	1.4%	0.00 [-0.98, 0.98]
Cetin 2017	1	0.5	16	16	1.4%	1.00 [0.02, 1.98]
Erbey 2016	0.56	0.0867	18	32	13.2%	0.56 [0.39, 0.73]
Fang 2007	0.6	0.2194	3	5	5.5%	0.60 [0.17, 1.03]
Kurtzberg 2014	0.88	0.051	36	41	15.9%	0.88 [0.78, 0.98]
KuÇi 2016	0.87	0.0918	13	15	12.8%	0.87 [0.69, 1.05]
Lucchini 2010	0.67	0.1939	4	6	6.5%	0.67 [0.29, 1.05]
Muroi 2013	0.56	0.1684	5	9	7.7%	0.56 [0.23, 0.89]
Muroi 2016	0.58	0.1429	7	12	9.2%	0.58 [0.30, 0.86]
Müller 2008	0.5	0.1582	1	2	8.3%	0.50 [0.19, 0.81]
Prasad 2011	0.4	0.2194	2	5	5.5%	0.40 [-0.03, 0.83]
Pérez-Simon 2011	0	0.5	0	4	1.4%	0.00 [-0.98, 0.98]
Ringdén 2006	0.75	0.2143	3	4	5.7%	0.75 [0.33, 1.17]
Sánchez-Guijo 2014	0.47	0.23	8	17	5.2%	0.47 [0.02, 0.92]
**Subtotal (95% CI)**			**116**	**170**	**100.0%**	**0.64 [0.52, 0.77]**
Heterogeneity: Tau² = 0.02; Chi² = 27.52, df = 13 (P = 0.01); I² = 53%						
Test for overall effect: Z = 10.36 (P < 0.00001)						
**Liver**						
Arima 2010	0	0.5	0	1	2.3%	0.00 [-0.98, 0.98]
Cetin 2017	0.86	0.0969	12	14	14.6%	0.86 [0.67, 1.05]
Erbey 2016	0.36	0.1276	5	14	12.6%	0.36 [0.11, 0.61]
Fang 2007	1	0.5	4	4	2.3%	1.00 [0.02, 1.98]
Herrmann 2012	0	0.5	0	1	2.3%	0.00 [-0.98, 0.98]
Kurtzberg 2014	0.78	0.0816	21	27	15.5%	0.78 [0.62, 0.94]
KuÇi 2016	0.78	0.1378	7	9	12.0%	0.78 [0.51, 1.05]
Muroi 2013	0.5	0.352	1	2	4.1%	0.50 [-0.19, 1.19]
Muroi 2013	1	0.5	1	1	2.3%	1.00 [0.02, 1.98]
Muroi 2016	0.4	0.2194	2	5	7.8%	0.40 [-0.03, 0.83]
Müller 2008	1	0.5	1	1	2.3%	1.00 [0.02, 1.98]
Prasad 2011	0.25	0.2143	1	4	8.0%	0.25 [-0.17, 0.67]
Pérez-Simon 2011	0	0.5	0	2	2.3%	0.00 [-0.98, 0.98]
Ringdén 2006	0	0.5	0	1	2.3%	0.00 [-0.98, 0.98]
Sánchez-Guijo 2014	0.43	0.1888	3	7	9.2%	0.43 [0.06, 0.80]
**Subtotal (95% CI)**			**58**	**93**	**100.0%**	**0.57 [0.41, 0.72]**
Heterogeneity: Tau² = 0.04; Chi² = 27.82, df = 14 (P = 0.02); I² = 50%						
Test for overall effect: Z = 6.99 (P < 0.00001)						
**Gut**						
Arima 2010	0	0.5	0	3	2.0%	0.00 [-0.98, 0.98]
Cetin 2017	0.88	0.1173	7	8	8.1%	0.88 [0.65, 1.11]
Erbey 2016	0.52	0.0918	15	29	8.7%	0.52 [0.34, 0.70]
Fang 2007	0.33	0.2704	1	3	4.6%	0.33 [-0.20, 0.86]
Herrmann 2012	0.5	0.1786	4	8	6.6%	0.50 [0.15, 0.85]
Kurtzberg 2014	0.86	0.0561	56	65	9.4%	0.86 [0.75, 0.97]
KuÇi 2016	0.79	0.0816	19	24	8.9%	0.79 [0.63, 0.95]
Lucchini 2010	0.2	0.1786	1	4	6.6%	0.20 [-0.15, 0.55]
Muroi 2013	0	0.5	1	1	2.0%	0.00 [-0.98, 0.98]
Muroi 2016	0.6	0.1071	12	20	8.3%	0.60 [0.39, 0.81]
Müller 2008	0.5	0.352	1	2	3.4%	0.50 [-0.19, 1.19]
Prasad 2011	0.33	0.1327	4	12	7.7%	0.33 [0.07, 0.59]
Pérez-Simon 2011	0.1	0.0969	1	10	8.6%	0.10 [-0.09, 0.29]
Ringdén 2006	0.63	0.1735	5	8	6.7%	0.63 [0.29, 0.97]
Sánchez-Guijo 2014	0.4	0.1071	8	20	8.3%	0.40 [0.19, 0.61]
**Subtotal (95% CI)**			**135**	**217**	**100.0%**	**0.51 [0.35, 0.66]**
Heterogeneity: Tau² = 0.07; Chi² = 76.80, df = 14 (P < 0.00001); I² = 82%						
Test for overall effect: Z = 6.33 (P < 0.00001)						

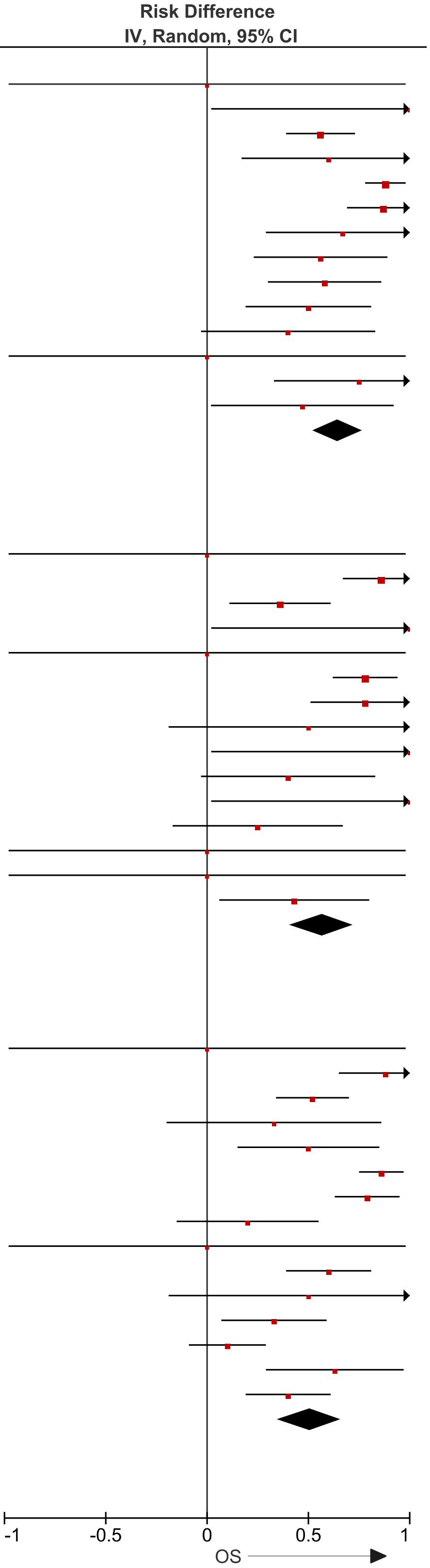

Test for subgroup differences: Chi² = 1.97, df = 2 (P = 0.37), I² = 0%

Forest-plot of organ-specific overall survival of patients with aGvHD from the MSC group at last follow-up. Dots and black lines represent the effect and 95% CI of individual studies. Black diamonds represent the overall effect size. Weight are from random-effects analysis.

Figure S12. Overall survival of aGvHD patients with multiorgan affection from the MSC group at last follow-up.

Study or Subgroup	Risk Difference	SE	Surviving patients	Total patients	Weight	Risk Difference IV, Random, 95% CI
**Skin+Liver**						
Herrmann 2012	1	0.5	2	2	33.3%	1.00 [0.02, 1.98]
Muroi 2016	0	0.5	0	2	33.3%	0.00 [-0.98, 0.98]
Sánchez-Guijo 2014	0	0.5	0	1	33.3%	0.00 [-0.98, 0.98]
**Subtotal (95% CI)**			**2**	**5**	**100.0%**	**0.33 [-0.32, 0.99]**
Heterogeneity: Tau² = 0.08; Chi² = 2.67, df = 2 (P = 0.26); I² = 25%						
Test for overall effect: Z = 1.00 (P = 0.32)						
**Skin+Gut**						
Erbey 2016	0.64	0.1276	9	14	23.7%	0.64 [0.39, 0.89]
Fang 2007	0	0.5	0	2	1.5%	0.00 [-0.98, 0.98]
Herrmann 2012	0.5	0.2041	3	6	9.3%	0.50 [0.10, 0.90]
KuÇi 2016	0.75	0.1531	6	8	16.5%	0.75 [0.45, 1.05]
Lucchini 2010	0	0.5	0	2	1.5%	0.00 [-0.98, 0.98]
Muroi 2013	0.5	0.2041	3	6	9.3%	0.50 [0.10, 0.90]
Muroi 2016	0.57	0.1888	4	7	10.8%	0.57 [0.20, 0.94]
Müller 2008	0	0.5	0	1	1.5%	0.00 [-0.98, 0.98]
Prasad 2011	0.5	0.352	1	2	3.1%	0.50 [-0.19, 1.19]
Pérez-Simon 2011	0	0.5	0	2	1.5%	0.00 [-0.98, 0.98]
Ringdén 2006	0.75	0.2143	3	4	8.4%	0.75 [0.33, 1.17]
Sánchez-Guijo 2014	0.38	0.1735	3	8	12.8%	0.38 [0.04, 0.72]
**Subtotal (95% CI)**			**32**	**62**	**100.0%**	**0.56 [0.43, 0.68]**
Heterogeneity: Tau² = 0.00; Chi² = 9.02, df = 11 (P = 0.62); I² = 0%						
Test for overall effect: Z = 8.96 (P < 0.00001)						
**Liver+Gut**						
Arima 2010	0	0.5	0	1	7.4%	0.00 [-0.98, 0.98]
Herrmann 2012	0	0.5	0	1	7.4%	0.00 [-0.98, 0.98]
KuÇi 2016	0.33	0.2704	1	3	25.4%	0.33 [-0.20, 0.86]
Muroi 2013	0	0.5	0	1	7.4%	0.00 [-0.98, 0.98]
Muroi 2013	1	0.5	1	1	7.4%	1.00 [0.02, 1.98]
Muroi 2016	0	0.5	0	2	7.4%	0.00 [-0.98, 0.98]
Prasad 2011	0.5	0.352	1	2	15.0%	0.50 [-0.19, 1.19]
Ringdén 2006	0	0.5	0	1	7.4%	0.00 [-0.98, 0.98]
Sánchez-Guijo 2014	0.5	0.352	1	2	15.0%	0.50 [-0.19, 1.19]
**Subtotal (95% CI)**			**4**	**14**	**100.0%**	**0.31 [0.04, 0.58]**
Heterogeneity: Tau² = 0.00; Chi² = 4.41, df = 8 (P = 0.82); I² = 0%						
Test for overall effect: Z = 2.26 (P = 0.02)						
**Skin+Liver+Gut**						
Arima 2010	0	0.5	0	1	3.8%	0.00 [-0.98, 0.98]
Erbey 2016	0.36	0.1276	5	14	58.3%	0.36 [0.11, 0.61]
Fang 2007	1	0.5	1	1	3.8%	1.00 [0.02, 1.98]
KuÇi 2016	1	0.5	6	6	3.8%	1.00 [0.02, 1.98]
Muroi 2016	1	0.5	1	1	3.8%	1.00 [0.02, 1.98]
Müller 2008	1	0.5	1	1	3.8%	1.00 [0.02, 1.98]
Prasad 2011	0	0.5	0	2	3.8%	0.00 [-0.98, 0.98]
Pérez-Simon 2011	0	0.5	0	2	3.8%	0.00 [-0.98, 0.98]
Sánchez-Guijo 2014	0.5	0.25	2	4	15.2%	0.50 [0.01, 0.99]
**Subtotal (95% CI)**			**16**	**32**	**100.0%**	**0.44 [0.25, 0.63]**
Heterogeneity: Tau² = 0.00; Chi² = 7.79, df = 8 (P = 0.45); I² = 0%						
Test for overall effect: Z = 4.49 (P < 0.00001)						

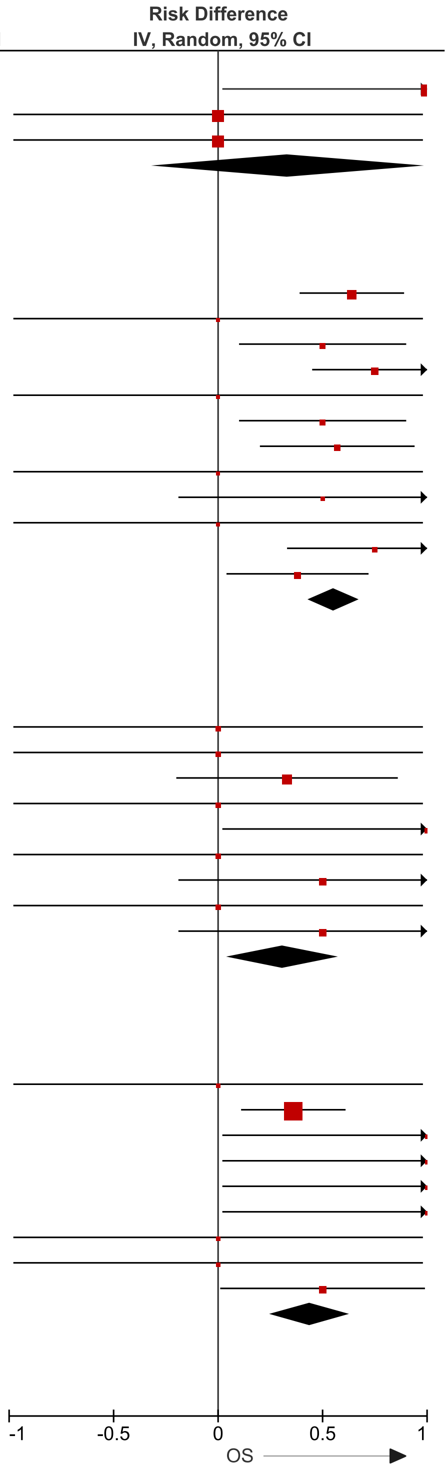

Test for subgroup differences: Chi² = 3.44, df = 3 (P = 0.33), I² = 12.9%

Forest-plot of overall survival of aGvHD patients with multiorgan affection from the MSC group at last follow-up. Dots and black lines represent the effect and 95% CI of individual studies. Black diamonds represent the overall effect size. Weight are from random-effects analysis.

Figure S13. Overall survival and first day of infusion of MSC from HSCT of patients with aGvHD.

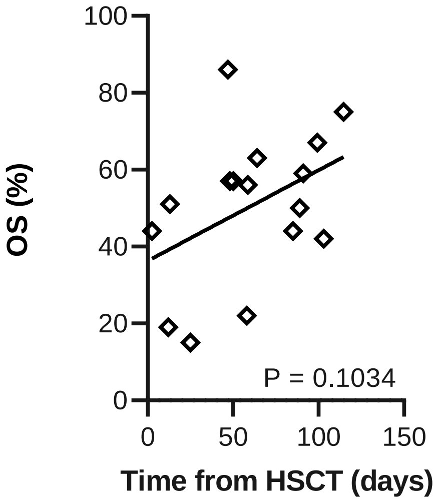

Correlation between overall survival of patients with aGvHD at last follow-up and time for first day of infusion of MSC from HSCT. Diamonds represent individual studies.

Figure S14. Overall and complete responses of patients with aGvHD from the MSC group.

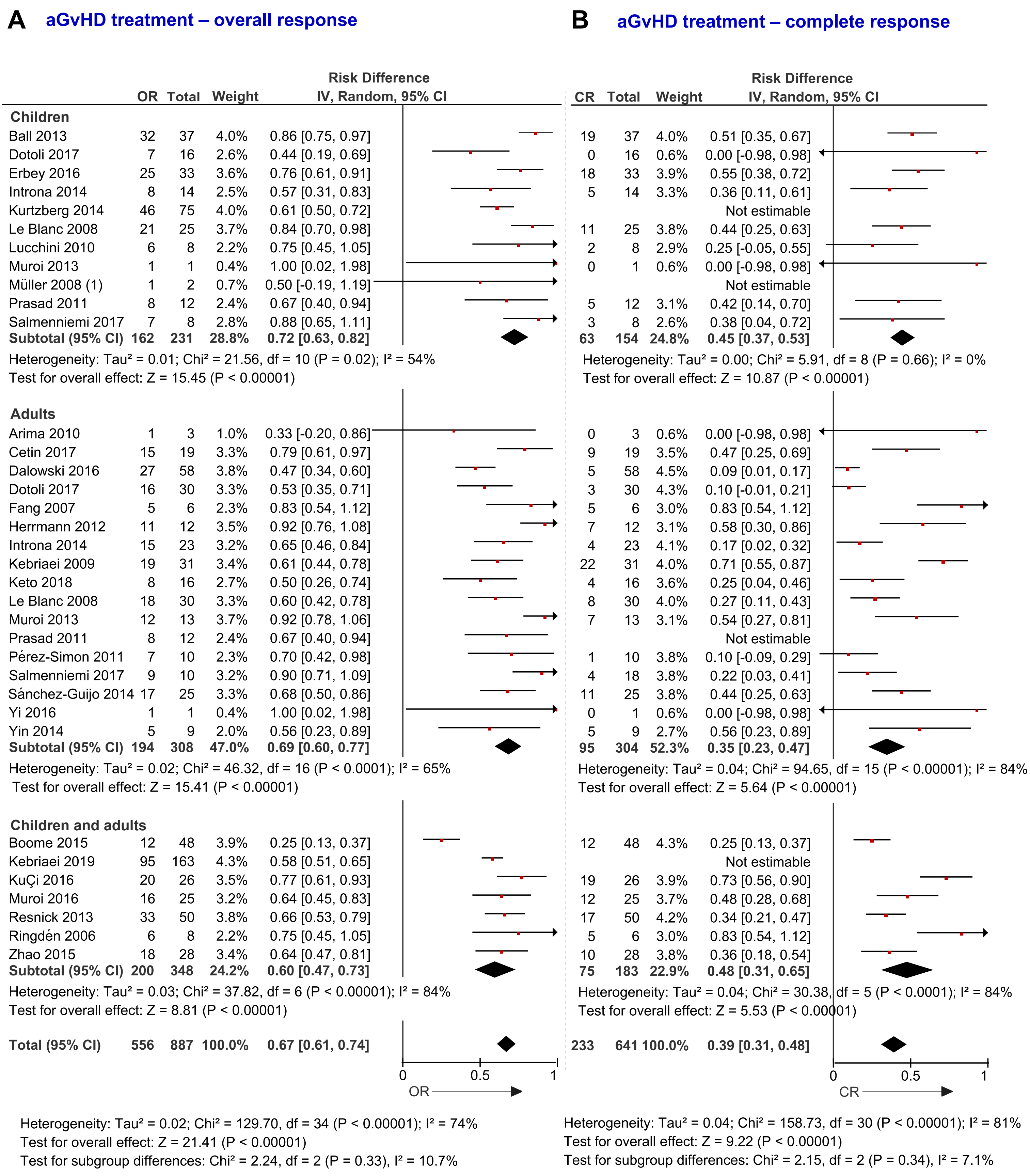

Forest-plots showing the overall response (A) and complete response (B) of patients with aGvHD infused with MSC. Dots and black lines represent the effect and 95% CI of individual studies. Black diamonds represent the overall effect size. Weight are from random-effects analysis.

Figure S15. Grade-specific overall response of patients with aGvHD from the MSC group at last follow-up.

Study or Subgroup	Risk Difference	SE	Responders	Total	Weight	Risk Difference IV, Random, 95% CI
**Grade II**						
Herrmann 2012	1	0.5	1	1	3.4%	1.00 [0.02, 1.98]
Introna 2014	1	0.5	2	2	3.4%	1.00 [0.02, 1.98]
Introna 2014	0.78	0.1378	7	9	45.2%	0.78 [0.51, 1.05]
KuÇi 2016	1	0.5	1	1	3.4%	1.00 [0.02, 1.98]
Muroi 2013	1	0.5	9	9	3.4%	1.00 [0.02, 1.98]
Müller 2008	0	0.5	0	1	3.4%	0.00 [-0.98, 0.98]
Pérez-Simon 2011	0.67	0.2755	2	3	11.3%	0.67 [0.13, 1.21]
Ringdén 2006	0	0.5	0	1	3.4%	0.00 [-0.98, 0.98]
Sánchez-Guijo 2014	0.67	0.1939	4	6	22.8%	0.67 [0.29, 1.05]
**Subtotal (95% CI)**			**26**	**33**	**100.0%**	**0.72 [0.54, 0.90]**
Heterogeneity: Tau² = 0.00; Chi² = 5.69, df = 8 (P = 0.68); I² = 0%						
Test for overall effect: Z = 7.76 (P < 0.00001)						
**Grade III**						
Fang 2007	0.5	0.0867	1	2	30.1%	0.50 [0.33, 0.67]
Herrmann 2012	1	0.5	6	6	0.9%	1.00 [0.02, 1.98]
KuÇi 2016	0.64	0.148	7	11	10.3%	0.64 [0.35, 0.93]
Muroi 2013	1	0.5	1	1	0.9%	1.00 [0.02, 1.98]
Muroi 2013	0.75	0.2143	3	4	4.9%	0.75 [0.33, 1.17]
Muroi 2016	0.64	0.102	14	22	21.7%	0.64 [0.44, 0.84]
Müller 2008	1	0.5	1	1	0.9%	1.00 [0.02, 1.98]
Prasad 2011	0.75	0.2143	3	4	4.9%	0.75 [0.33, 1.17]
Pérez-Simon 2011	1	0.5	3	3	0.9%	1.00 [0.02, 1.98]
Ringdén 2006	0.8	0.1786	4	5	7.1%	0.80 [0.45, 1.15]
Sánchez-Guijo 2014	0.71	0.1173	10	14	16.4%	0.71 [0.48, 0.94]
Von Bonin 2009	0	0.5	0	1	0.9%	0.00 [-0.98, 0.98]
**Subtotal (95% CI)**			**53**	**74**	**100.0%**	**0.64 [0.55, 0.73]**
Heterogeneity: Tau² = 0.00; Chi² = 8.00, df = 11 (P = 0.71); I² = 0%						
Test for overall effect: Z = 13.44 (P < 0.00001)						
**Grade IV**						
Fang 2007	1	0.5	4	4	1.3%	1.00 [0.02, 1.98]
Herrmann 2012	0.75	0.2143	3	4	7.2%	0.75 [0.33, 1.17]
KuÇi 2016	0.86	0.0969	12	14	35.3%	0.86 [0.67, 1.05]
Muroi 2016	0.67	0.2755	2	3	4.4%	0.67 [0.13, 1.21]
Prasad 2011	0.71	0.1684	5	7	11.7%	0.71 [0.38, 1.04]
Pérez-Simon 2011	0.5	0.25	2	4	5.3%	0.50 [0.01, 0.99]
Ringdén 2006	1	0.5	1	1	1.3%	1.00 [0.02, 1.98]
Sánchez-Guijo 2014	0.43	0.1888	3	4	9.3%	0.43 [0.06, 0.80]
Von Bonin 2009	0.88	0.1173	7	8	24.1%	0.88 [0.65, 1.11]
**Subtotal (95% CI)**			**39**	**49**	**100.0%**	**0.78 [0.66, 0.89]**
Heterogeneity: Tau² = 0.00; Chi² = 6.83, df = 8 (P = 0.55); I² = 0%						
Test for overall effect: Z = 13.47 (P < 0.00001)						
**Grade III-IV**						
Fang 2007	0.83	0.148	5	6	5.8%	0.83 [0.54, 1.12]
Herrmann 2012	0.9	0.0969	9	10	13.6%	0.90 [0.71, 1.09]
Introna 2014	0.76	0.102	13	17	12.2%	0.76 [0.56, 0.96]
Introna 2014	0.33	0.2704	1	3	1.7%	0.33 [-0.20, 0.86]
KuÇi 2016	0.76	0.0867	19	25	16.9%	0.76 [0.59, 0.93]
Muroi 2013	1	0.5	1	1	0.5%	1.00 [0.02, 1.98]
Muroi 2013	0.75	0.2143	3	4	2.8%	0.75 [0.33, 1.17]
Muroi 2016	0.64	0.0969	16	25	13.6%	0.64 [0.45, 0.83]
Müller 2008	1	0.5	1	1	0.5%	1.00 [0.02, 1.98]
Prasad 2011	0.73	0.1378	8	11	6.7%	0.73 [0.46, 1.00]
Pérez-Simon 2011	0.71	0.38	5	7	0.9%	0.71 [-0.03, 1.45]
Ringdén 2006	0.83	0.148	5	6	5.8%	0.83 [0.54, 1.12]
Sánchez-Guijo 2014	0.72	0.102	13	18	12.2%	0.72 [0.52, 0.92]
Von Bonin 2009	0.78	0.1378	7	9	6.7%	0.78 [0.51, 1.05]
**Subtotal (95% CI)**			**106**	**143**	**100.0%**	**0.76 [0.69, 0.83]**
Heterogeneity: Tau² = 0.00; Chi² = 7.30, df = 13 (P = 0.89); I² = 0%						
Test for overall effect: Z = 21.28 (P < 0.00001)						

Test for subgroup differences: Chi² = 4.99, df = 3 (P = 0.17), I² = 39.9%

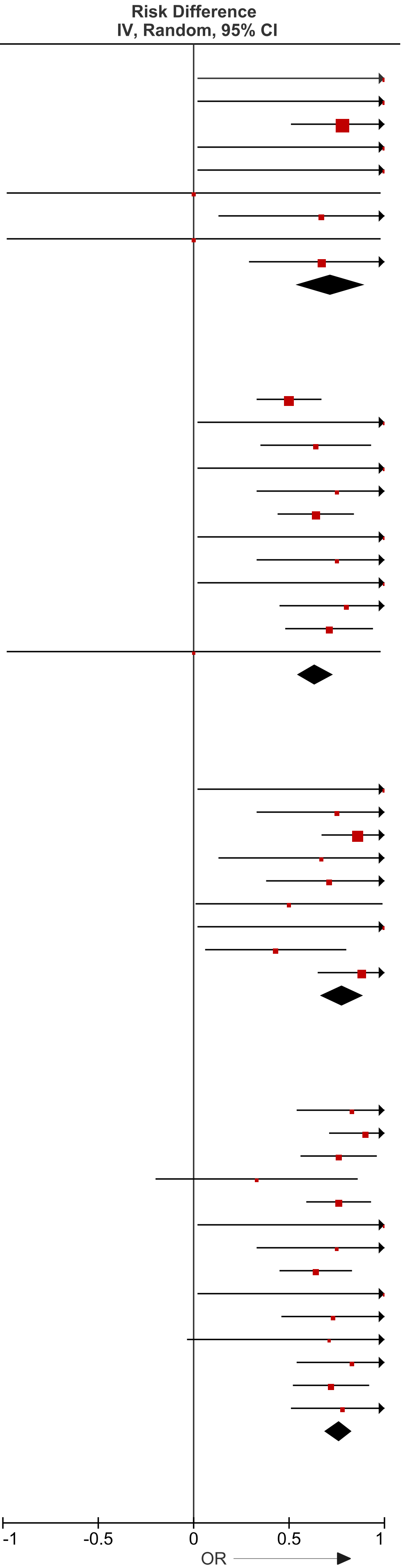

Forest-plot of grade-specific overall response of patients with aGvHD from the MSC group at last follow-up. Dots and black lines represent the effect and 95% CI of individual studies. Black diamonds represent the overall effect size. Weight are from random-effects analysis.

Figure S16. Overall response of patients with aGvHD from the MSC group regarding to the number of organs affected.

Study or Subgroup	Risk Difference	SE	OR	Total	Weight	Risk Difference IV, Random, 95% CI
**1 organ**						
Arima 2010	1	0.5	1	1	1.3%	1.00 [0.02, 1.98]
Erbey 2016	1	0.5	5	5	1.3%	1.00 [0.02, 1.98]
Fang 2007	1	0.5	1	1	1.3%	1.00 [0.02, 1.98]
Herrmann 2012	1	0.5	1	1	1.3%	1.00 [0.02, 1.98]
KuÇi 2016	0.44	0.1633	4	9	12.1%	0.44 [0.12, 0.76]
Lucchini 2010	0.67	0.1939	4	6	8.6%	0.67 [0.29, 1.05]
Muroi 2013	1	0.5	6	6	1.3%	1.00 [0.02, 1.98]
Muroi 2016	0.69	0.1276	9	13	19.8%	0.69 [0.44, 0.94]
Prasad 2011	0.75	0.2143	3	4	7.0%	0.75 [0.33, 1.17]
Pérez-Simon 2011	0.67	0.1939	4	6	8.6%	0.67 [0.29, 1.05]
Ringdén 2006	0.67	0.13	2	3	19.1%	0.67 [0.42, 0.92]
Sánchez-Guijo 2014	0.7	0.1429	7	10	15.8%	0.70 [0.42, 0.98]
Von Bonin 2009	0	0.5	0	1	1.3%	0.00 [-0.98, 0.98]
Yi 2016	1	0.5	1	1	1.3%	1.00 [0.02, 1.98]
**Subtotal (95% CI)**			**48**	**67**	**100.0%**	**0.67 [0.56, 0.78]**
Heterogeneity: Tau² = 0.00; Chi² = 6.60, df = 13 (P = 0.92); I² = 0%						
Test for overall effect: Z = 11.86 (P < 0.00001)						
**2 organs**						
Arima 2010	0	0.5	0	1	1.1%	0.00 [-0.98, 0.98]
Erbey 2016	0.86	0.0969	12	14	28.0%	0.86 [0.67, 1.05]
Fang 2007	0.75	0.2143	3	4	5.7%	0.75 [0.33, 1.17]
Herrmann 2012	0	0.5	6	6	1.1%	0.00 [-0.98, 0.98]
KuÇi 2016	0.89	0.1071	8	9	22.9%	0.89 [0.68, 1.10]
Lucchini 2010	0.5	0.352	1	2	2.1%	0.50 [-0.19, 1.19]
Muroi 2013	0.86	0.1327	6	7	14.9%	0.86 [0.60, 1.12]
Muroi 2016	0.67	0.36	6	9	2.0%	0.67 [-0.04, 1.38]
Müller 2008	0	0.5	0	1	1.1%	0.00 [-0.98, 0.98]
Prasad 2011	0.8	0.1786	4	5	8.2%	0.80 [0.45, 1.15]
Pérez-Simon 2011	0.5	0.352	1	2	2.1%	0.50 [-0.19, 1.19]
Ringdén 2006	0.8	0.1786	4	5	8.2%	0.80 [0.45, 1.15]
Sánchez-Guijo 2014	0.7	0.42	7	10	1.5%	0.70 [-0.12, 1.52]
Von Bonin 2009	1	0.5	7	7	1.1%	1.00 [0.02, 1.98]
**Subtotal (95% CI)**			**65**	**82**	**100.0%**	**0.80 [0.70, 0.90]**
Heterogeneity: Tau² = 0.00; Chi² = 10.82, df = 13 (P = 0.63); I² = 0%						
Test for overall effect: Z = 15.67 (P < 0.00001)						
**3 organs**						
Arima 2010	0	0.5	0	1	3.4%	0.00 [-0.98, 0.98]
Erbey 2016	0.57	0.1327	8	14	47.9%	0.57 [0.31, 0.83]
Fang 2007	1	0.5	1	1	3.4%	1.00 [0.02, 1.98]
Herrmann 2012	1	0.5	2	2	3.4%	1.00 [0.02, 1.98]
KuÇi 2016	1	0.5	8	8	3.4%	1.00 [0.02, 1.98]
Muroi 2016	1	0.5	1	1	3.4%	1.00 [0.02, 1.98]
Müller 2008	1	0.5	1	1	3.4%	1.00 [0.02, 1.98]
Prasad 2011	0.5	0.352	1	2	6.8%	0.50 [-0.19, 1.19]
Pérez-Simon 2011	1	0.5	2	2	3.4%	1.00 [0.02, 1.98]
Sánchez-Guijo 2014	0.75	0.2143	3	4	18.4%	0.75 [0.33, 1.17]
Von Bonin 2009	0	0.5	0	1	3.4%	0.00 [-0.98, 0.98]
**Subtotal (95% CI)**			**27**	**37**	**100.0%**	**0.65 [0.47, 0.83]**
Heterogeneity: Tau² = 0.00; Chi² = 7.08, df = 10 (P = 0.72); I² = 0%						
Test for overall effect: Z = 7.05 (P < 0.00001)						

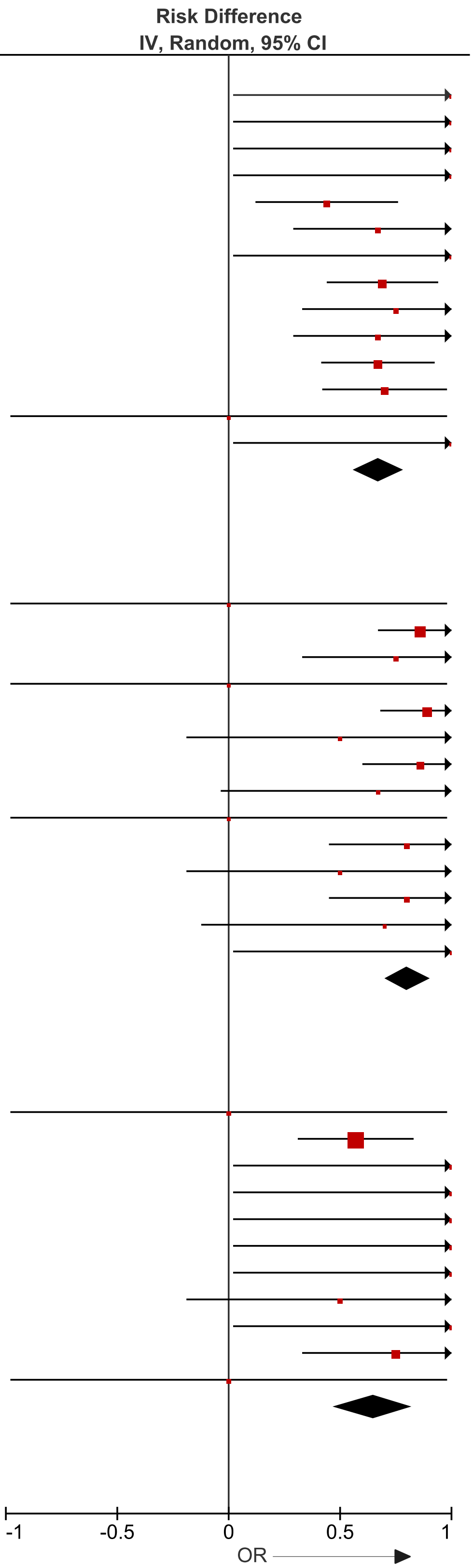

Test for subgroup differences: Chi² = 3.87, df = 2 (P = 0.14), I² = 48.3%

Forest-plot of overall response of patients with aGvHD from the MSC group regarding to the number of organs affected. Dots and black lines represent the effect and 95% CI of individual studies. Black diamonds represent the overall effect size. Weight are from random-effects analysis.

Figure S17. Complete response of patients with aGvHD from the MSC group regarding to the number of organ affected.

Study or Subgroup	Risk Difference	SE	CR	Total	Weight	Risk Difference IV, Random, 95% CI
**1 organ**						
Arima 2010	0	0.5	0	1	1.7%	0.00 [-0.98, 0.98]
Ball 2013	1	0.5	5	5	1.7%	1.00 [0.02, 1.98]
Erbey 2016	0.8	0.1786	4	5	9.3%	0.80 [0.45, 1.15]
Fang 2007	1	0.5	1	1	1.7%	1.00 [0.02, 1.98]
Herrmann 2012	1	0.5	1	1	1.7%	1.00 [0.02, 1.98]
KuÇi 2016	0.22	0.1378	2	9	12.5%	0.22 [-0.05, 0.49]
Lucchini 2010	0.5	0.2041	3	6	7.8%	0.50 [0.10, 0.90]
Muroi 2013	0.67	0.1939	4	6	8.4%	0.67 [0.29, 1.05]
Muroi 2016	0.31	0.1276	4	13	13.5%	0.31 [0.06, 0.56]
Prasad 2011	0.52	0.17	1	4	9.9%	0.52 [0.19, 0.85]
Pérez-Simon 2011	0.17	0.1531	1	6	11.2%	0.17 [-0.13, 0.47]
Ringdén 2006	0.67	0.2755	2	3	5.0%	0.67 [0.13, 1.21]
Sánchez-Guijo 2014	0.3	0.1429	3	10	12.1%	0.30 [0.02, 0.58]
Von Bonin 2009	0	0.5	0	1	1.7%	0.00 [-0.98, 0.98]
Yi 2016	0	0.5	0	1	1.7%	0.00 [-0.98, 0.98]
**Subtotal (95% CI)**			**31**	**72**	**100.0%**	**0.43 [0.30, 0.56]**
Heterogeneity: Tau² = 0.02; Chi² = 19.83, df = 14 (P = 0.14); I² = 29%						
Test for overall effect: Z = 6.31 (P < 0.00001)						
**2 organs**						
Arima 2010	0	0.5	0	1	1.7%	0.00 [-0.98, 0.98]
Ball 2013	0.73	0.1378	8	11	10.2%	0.73 [0.46, 1.00]
Erbey 2016	0.71	0.1173	10	14	11.5%	0.71 [0.48, 0.94]
Fang 2007	0.75	0.2143	3	4	6.4%	0.75 [0.33, 1.17]
Herrmann 2012	0.67	0.1939	4	6	7.2%	0.67 [0.29, 1.05]
KuÇi 2016	0.33	0.1531	3	9	9.3%	0.33 [0.03, 0.63]
Lucchini 2010	0	0.5	0	2	1.7%	0.00 [-0.98, 0.98]
Muroi 2013	0.57	0.1888	4	7	7.5%	0.57 [0.20, 0.94]
Muroi 2016	0.33	0.1531	3	9	9.3%	0.33 [0.03, 0.63]
Prasad 2011	0.2	0.1786	1	5	7.9%	0.20 [-0.15, 0.55]
Pérez-Simon 2011	0	0.5	0	2	1.7%	0.00 [-0.98, 0.98]
Ringdén 2006	0.6	0.2194	3	5	6.2%	0.60 [0.17, 1.03]
Sánchez-Guijo 2014	0.5	0.1582	5	10	9.0%	0.50 [0.19, 0.81]
Von Bonin 2009	0.14	0.1327	1	7	10.5%	0.14 [-0.12, 0.40]
**Subtotal (95% CI)**			**45**	**92**	**100.0%**	**0.47 [0.33, 0.60]**
Heterogeneity: Tau² = 0.03; Chi² = 23.93, df = 13 (P = 0.03); I² = 46%						
Test for overall effect: Z = 6.85 (P < 0.00001)						
**3 organs**						
Arima 2010	0	0.5	0	1	2.7%	0.00 [-0.98, 0.98]
Ball 2013	0.52	0.1071	11	21	35.2%	0.52 [0.31, 0.73]
Erbey 2016	0.29	0.1225	4	14	29.9%	0.29 [0.05, 0.53]
Fang 2007	1	0.5	1	1	2.7%	1.00 [0.02, 1.98]
Herrmann 2012	1	0.5	2	2	2.7%	1.00 [0.02, 1.98]
KuÇi 2016	0	0.5	0	8	2.7%	0.00 [-0.98, 0.98]
Muroi 2016	0	0.5	0	1	2.7%	0.00 [-0.98, 0.98]
Prasad 2011	0	0.5	0	2	2.7%	0.00 [-0.98, 0.98]
Pérez-Simon 2011	0	0.5	0	2	2.7%	0.00 [-0.98, 0.98]
Sánchez-Guijo 2014	0.75	0.2143	3	4	13.0%	0.75 [0.33, 1.17]
Von Bonin 2009	0	0.5	0	1	2.7%	0.00 [-0.98, 0.98]
**Subtotal (95% CI)**			**21**	**57**	**100.0%**	**0.42 [0.26, 0.59]**
Heterogeneity: Tau² = 0.01; Chi² = 11.28, df = 10 (P = 0.34); I² = 11%						
Test for overall effect: Z = 5.01 (P < 0.00001)						

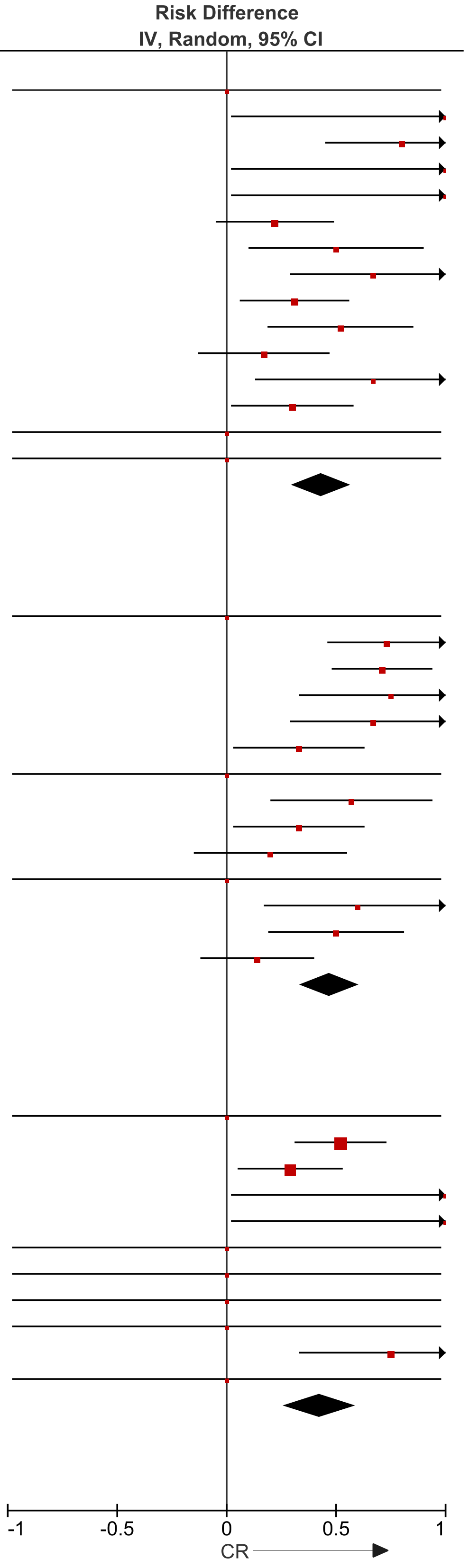

Forest-plot of complete response of patients with aGvHD from the MSC group regarding to the number of organs affected. Dots and black lines represent the effect and 95% CI of individual studies. Black diamonds represent the overall effect size. Weight are from random-effects analysis.

Figure S18. Complete response of patients with aGvHD grade II vs grade III-IV.

	Grade III-IV		Grade II			Risk Ratio
Study or Subgroup	Events	Total	Events	Total	Weight	M-H, Random, 95% CI
Herrmann 2012	6	10	1	1	12.7%	0.79 [0.31, 2.02]
Introna 2014	3	20	6	11	8.1%	0.28 [0.08, 0.89]
Kebriaei 2009	5	8	19	22	35.4%	0.72 [0.41, 1.27]
KuÇi 2016	5	25	0	1	1.8%	0.85 [0.07, 10.44]
Muroi 2013	8	14	6	9	26.6%	0.86 [0.45, 1.64]
Pérez-Simon 2011	1	7	0	3	1.3%	1.50 [0.08, 29.15]
Ringdén 2006	5	6	0	1	1.9%	3.14 [0.28, 35.75]
Sánchez-Guijo 2014	8	18	3	6	12.3%	0.89 [0.34, 2.30]
**Total (95% CI)**		**108**		**54**	**100.0%**	**0.76 [0.54, 1.05]**
Total events	41		35			

Heterogeneity: Tau² = 0.00; Chi² = 4.67, df = 7 (P = 0.70); I² = 0%

Test for overall effect: Z = 1.65 (P = 0.10)

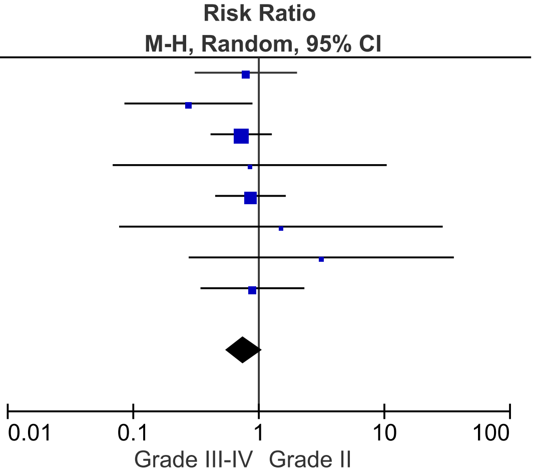

Forest-plot of complete response of patients from the MSC group with aGvHD II compared with those with grade III-IV. Dots and black lines represent the effect and 95% CI of individual studies. Black diamonds represent the overall effect size. Weight are from random-effects analysis.

Figure S19. Organ-specific overall and complete responses of patients with aGvHD from the MSC group.

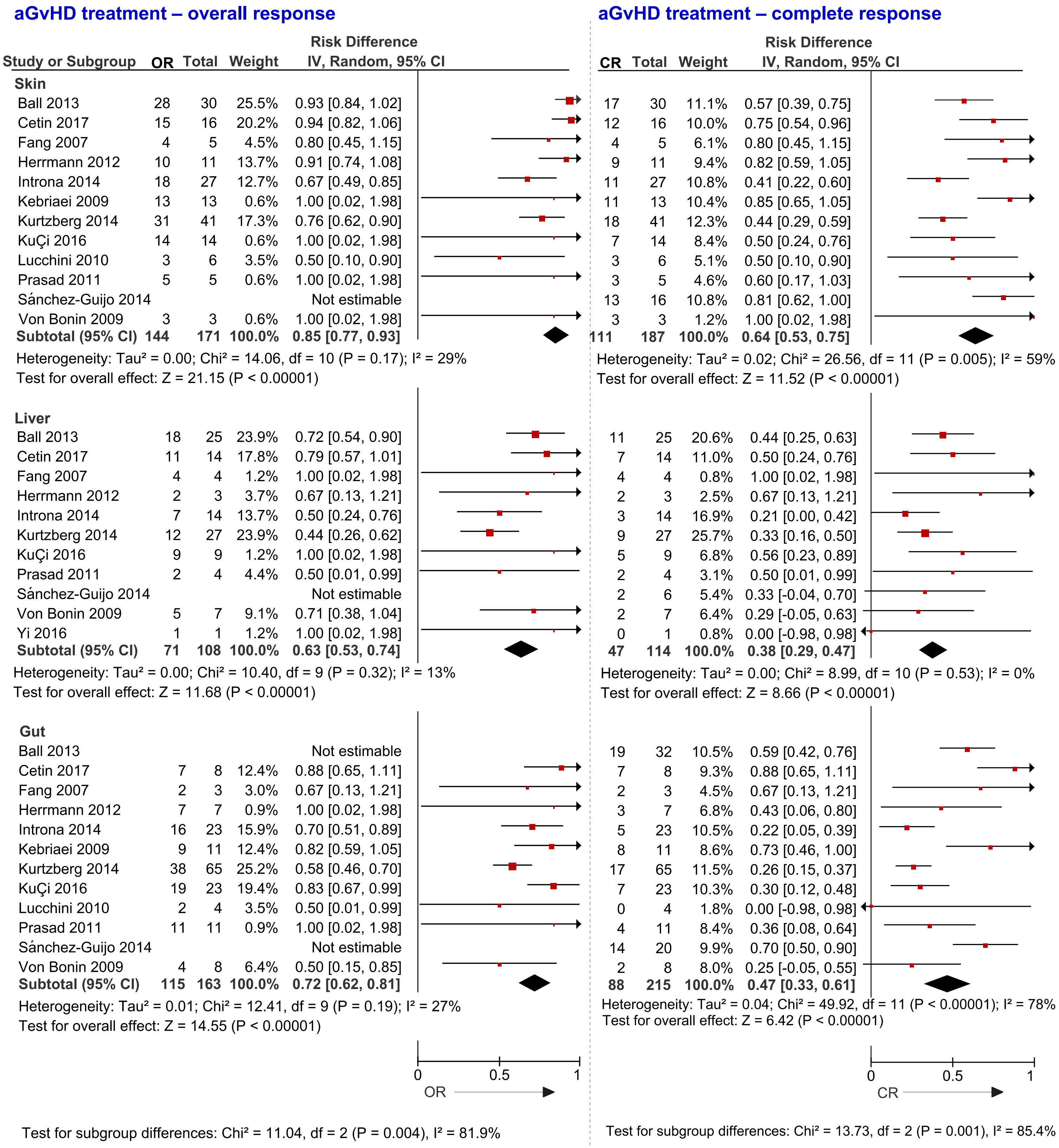

Forest-plots showing the organ-specific overall (A) and complete (B) responses of patients with aGvHD infused with MSC. Dots and black lines represent the effect and 95% CI of individual studies. Black diamonds represent the overall effect size. Weight are from random-effects analysis.

Figure S20. Gut-specific overall response of patients with aGvHD from the MSC group.

Study or Subgroup	Risk Difference	SE	Responders	Total	Weight	Risk Difference IV, Random, 95% CI
**Children**						
Introna 2014	0.8	0.1786	4	5	3.5%	0.80 [0.45, 1.15]
Kurtzberg 2014	0.58	0.0612	38	65	28.1%	0.58 [0.46, 0.70]
Lucchini 2010	0.5	0.25	2	4	1.8%	0.50 [0.01, 0.99]
Muroi 2013	1	0.5	1	1	0.4%	1.00 [0.02, 1.98]
Müller 2008	0.5	0.19	1	2	3.1%	0.50 [0.13, 0.87]
Prasad 2011	0.73	0.1378	8	11	5.8%	0.73 [0.46, 1.00]
**Subtotal (95% CI)**			**54**	**88**	**42.6%**	**0.61 [0.51, 0.71]**
Heterogeneity: Tau² = 0.00; Chi² = 3.26, df = 5 (P = 0.66); I² = 0%						
Test for overall effect: Z = 12.23 (P < 0.00001)						
**Adults**						
Arima 2010	0.33	0.2704	1	3	1.5%	0.33 [-0.20, 0.86]
Cetin 2017	0.88	0.1173	7	8	8.0%	0.88 [0.65, 1.11]
Fang 2007	0.67	0.2755	2	3	1.5%	0.67 [0.13, 1.21]
Herrmann 2012	1	0.5	8	8	0.4%	1.00 [0.02, 1.98]
Introna 2014	0.67	0.1122	12	18	8.7%	0.67 [0.45, 0.89]
Kebriaei 2009	0.82	0.1173	9	11	8.0%	0.82 [0.59, 1.05]
Muroi 2013	0.89	0.1071	8	9	9.5%	0.89 [0.68, 1.10]
Pérez-Simon 2011	0.7	0.1429	7	10	5.4%	0.70 [0.42, 0.98]
Sánchez-Guijo 2014	0.7	0.102	14	20	10.5%	0.70 [0.50, 0.90]
Von Bonin 2009	0.71	0.1684	5	7	3.9%	0.71 [0.38, 1.04]
**Subtotal (95% CI)**			**73**	**97**	**57.4%**	**0.76 [0.68, 0.85]**
Heterogeneity: Tau² = 0.00; Chi² = 6.90, df = 9 (P = 0.65); I² = 0%						
Test for overall effect: Z = 17.41 (P < 0.00001)						
**Total (95% CI)**			**127**	**185**	**100.0%**	**0.70 [0.63, 0.76]**
Heterogeneity: Tau² = 0.00; Chi² = 15.17, df = 15 (P = 0.44); I² = 1%						
Test for overall effect: Z = 20.93 (P < 0.00001)						
Test for subgroup differences: Chi² = 5.01, df = 1 (P = 0.03), I² = 80.0%						

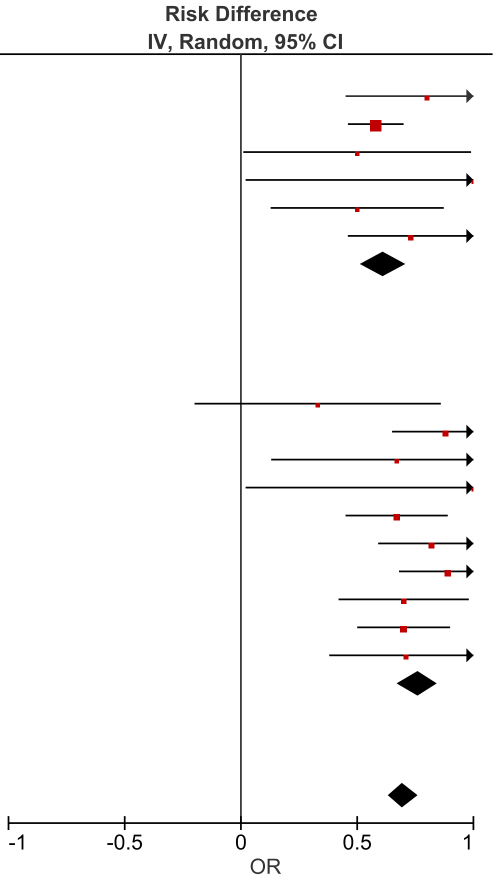

Forest-plot of gut-specific overall response of patients with aGvHD from the MSC group. Dots and black lines represent the effect and 95% CI of individual studies. Black diamonds represent the overall effect size. Weight are from random-effects analysis.

Figure S21. Correlation between responder rates and MSC dose or time from HSCT

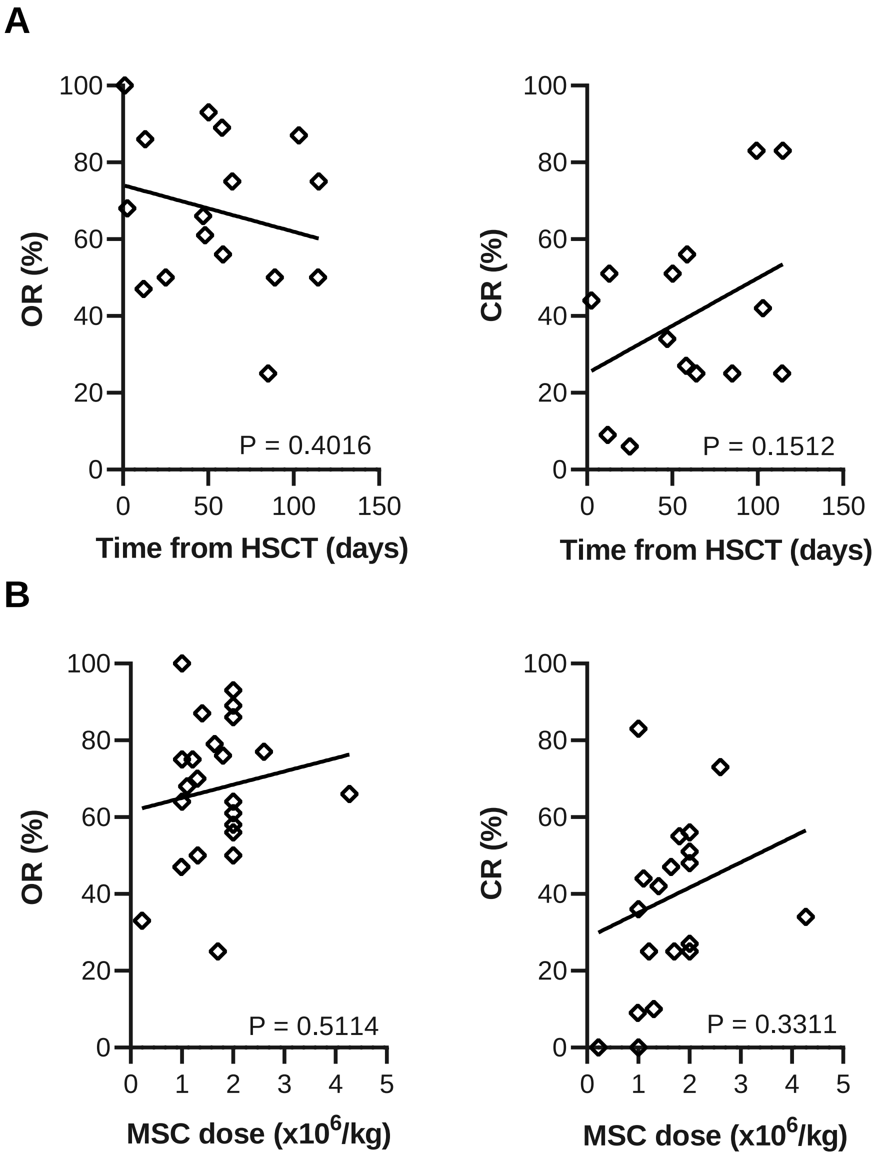

Correlation between the rate of responder patients with aGvHD and time from first day of infusion of MSC from HSCT (A) and MSC dose (B). Diamond represents individual studies.

Figure S22. Outome of aGvHD vs cGvHD patients.

A

GvHD treatment – overall survival: cGvHD vs aGvHD

Study or Subgroup	cGvHD Events	cGvHD Total	aGvHD Events	aGvHD Total	Weight	Risk Ratio M-H, Random, 95% CI
**Children**						
Lucchini 2010	3	3	5	8	21.2%	1.43 [0.76, 2.71]
Müller 2008	1	3	1	2	2.2%	0.67 [0.08, 5.54]
**Subtotal (95% CI)**		**6**		**10**	**23.5%**	**1.34 [0.73, 2.48]**
Total events	4		6			
Heterogeneity: Tau² = 0.00; Chi² = 0.54, df = 1 (P = 0.46); I² = 0%						
Test for overall effect: Z = 0.94 (P = 0.34)						
**Adults**						
Cetin 2017	5	5	17	19	65.4%	1.05 [0.78, 1.40]
Herrmann 2012	2	7	6	12	5.8%	0.57 [0.16, 2.10]
Pérez-Simon 2011	5	8	2	10	5.4%	3.13 [0.81, 12.06]
**Subtotal (95% CI)**		**20**		**41**	**76.5%**	**1.15 [0.56, 2.35]**
Total events	12		25			
Heterogeneity: Tau² = 0.20; Chi² = 3.66, df = 2 (P = 0.16); I² = 45%						
Test for overall effect: Z = 0.37 (P = 0.71)						
**Total (95% CI)**		**26**		**51**	**100.0%**	**1.13 [0.83, 1.56]**
Total events	16		31			
Heterogeneity: Tau² = 0.02; Chi² = 4.44, df = 4 (P = 0.35); I² = 10%						
Test for overall effect: Z = 0.78 (P = 0.44)						
Test for subgroup differences: Chi² = 0.11, df = 1 (P = 0.74), I² = 0%						

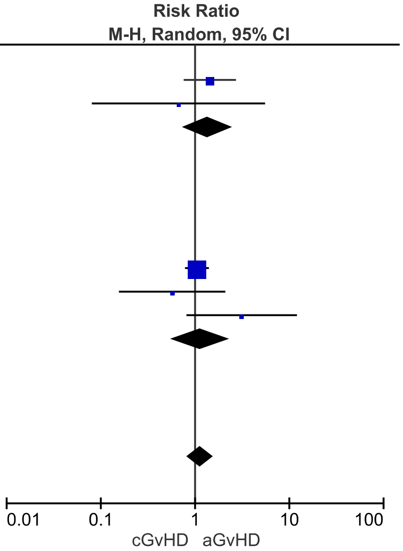

B

GvHD treatment – overall response: cGvHD vs aGvHD

Study or Subgroup	cGvHD Events	cGvHD Total	aGvHD Events	aGvHD Total	Weight	Risk Ratio M-H, Random, 95% CI
**Children**						
Introna 2014	0	1	8	14	1.7%	0.44 [0.04, 5.07]
Lucchini 2010	2	3	6	8	12.4%	0.89 [0.36, 2.17]
Müller 2008	1	3	1	2	2.2%	0.67 [0.08, 5.54]
**Subtotal (95% CI)**		**7**		**24**	**16.2%**	**0.80 [0.36, 1.74]**
Total events	3		15			
Heterogeneity: Tau² = 0.00; Chi² = 0.39, df = 2 (P = 0.82); I² = 0%						
Test for overall effect: Z = 0.57 (P = 0.57)						
**Adults**						
Cetin 2017	4	5	15	17	44.5%	0.91 [0.57, 1.45]
Herrmann 2012	4	7	11	12	22.4%	0.62 [0.32, 1.21]
Introna 2014	0	2	15	23	1.5%	0.26 [0.02, 3.30]
Pérez-Simon 2011	4	8	7	10	15.3%	0.71 [0.32, 1.59]
**Subtotal (95% CI)**		**22**		**62**	**83.8%**	**0.77 [0.54, 1.08]**
Total events	12		48			
Heterogeneity: Tau² = 0.00; Chi² = 2.14, df = 3 (P = 0.54); I² = 0%						
Test for overall effect: Z = 1.51 (P = 0.13)						
**Total (95% CI)**		**29**		**86**	**100.0%**	**0.77 [0.56, 1.06]**
Total events	15		63			
Heterogeneity: Tau² = 0.00; Chi² = 2.52, df = 6 (P = 0.87); I² = 0%						
Test for overall effect: Z = 1.61 (P = 0.11)						
Test for subgroup differences: Chi² = 0.01, df = 1 (P = 0.93), I² = 0%						

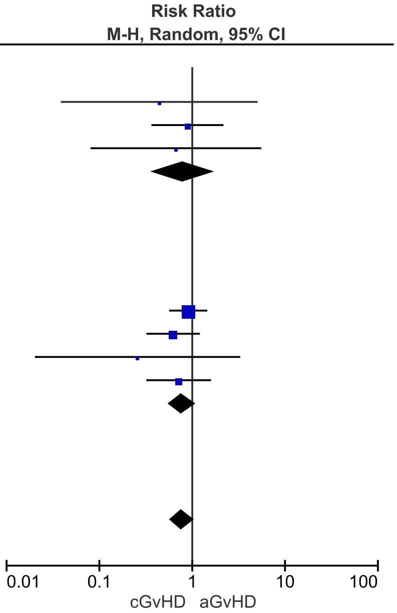

C

GvHD treatment – complete response: cGvHD vs aGvHD

Study or Subgroup	cGvHD Events	cGvHD Total	aGvHD Events	aGvHD Total	Weight	Risk Ratio M-H, Random, 95% CI
**Children**						
Introna 2014	0	1	5	14	8.3%	0.68 [0.06, 8.23]
Lucchini 2010	0	3	2	8	6.6%	0.45 [0.03, 7.39]
**Subtotal (95% CI)**		**4**		**22**	**14.9%**	**0.57 [0.09, 3.65]**
Total events	0		7			
Heterogeneity: Tau² = 0.00; Chi² = 0.05, df = 1 (P = 0.83); I² = 0%						
Test for overall effect: Z = 0.60 (P = 0.55)						
**Adults**						
Cetin 2017	2	5	9	17	38.1%	0.76 [0.24, 2.42]
Herrmann 2012	2	7	7	12	32.2%	0.49 [0.14, 1.74]
Introna 2014	0	2	4	23	7.3%	0.89 [0.06, 12.76]
Pérez-Simon 2011	1	8	1	10	7.6%	1.25 [0.09, 17.02]
**Subtotal (95% CI)**		**22**		**62**	**85.1%**	**0.68 [0.31, 1.48]**
Total events	5		21			
Heterogeneity: Tau² = 0.00; Chi² = 0.54, df = 3 (P = 0.91); I² = 0%						
Test for overall effect: Z = 0.97 (P = 0.33)						
**Total (95% CI)**		**26**		**84**	**100.0%**	**0.66 [0.32, 1.36]**
Total events	5		28			
Heterogeneity: Tau² = 0.00; Chi² = 0.62, df = 5 (P = 0.99); I² = 0%						
Test for overall effect: Z = 1.13 (P = 0.26)						
Test for subgroup differences: Chi² = 0.03, df = 1 (P = 0.86), I² = 0%						

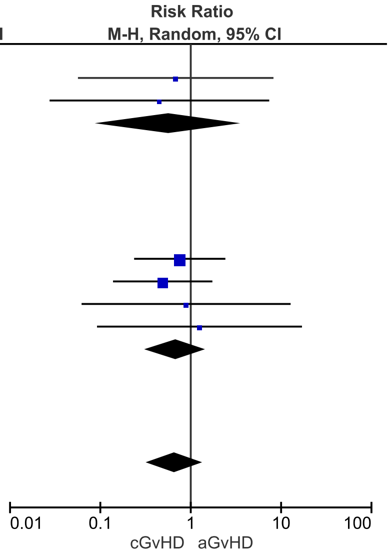

Forest-plots of overall survival at last follow-up (A), overall (B) and complete (C) responses of patients from the MSC group with aGvHD compared with those with cGvHD. Dots and black lines represent the effect and 95% CI of individual studies. Black diamonds represent the overall effect size. Weight are from random-effects analysis.

Supplementary Tables.

Table S1. Detailed search strategy.

Medline		
Database(s): Medline (No publication date restriction).<br><br>Search Strategy:		
**#**	**Searches**	**Results**
1	"MSC"[All Fields] AND "GvHD"[All Fields] AND (Clinical Trial[ptyp] OR Case Reports[ptyp] OR Clinical Study[ptyp] OR Comparative Study[ptyp] OR Controlled Clinical Trial[ptyp] OR Dataset[ptyp] OR Meta-Analysis[ptyp] OR Randomized Controlled Trial[ptyp])	38
2	(("mesenchymal stem cells"[MeSH Terms] OR ("mesenchymal"[All Fields] AND "stem"[All Fields] AND "cells"[All Fields]) OR "mesenchymal stem cells"[All Fields] OR ("mesenchymal"[All Fields] AND "stem"[All Fields] AND "cell"[All Fields]) OR "mesenchymal stem cell"[All Fields]) AND GVHD[All Fields]) AND (Clinical Trial[ptyp] OR Case Reports[ptyp] OR Clinical Study[ptyp] OR Comparative Study[ptyp] OR Controlled Clinical Trial[ptyp] OR Dataset[ptyp] OR Meta-Analysis[ptyp] OR Randomized Controlled Trial[ptyp])	62
3	(Mesenchymal[All Fields] AND stroma[All Fields] AND ("cells"[MeSH Terms] OR "cells"[All Fields] OR "cell"[All Fields]) AND GvHD[All Fields]) AND (Clinical Trial[ptyp] OR Case Reports[ptyp] OR Clinical Study[ptyp] OR Comparative Study[ptyp] OR Controlled Clinical Trial[ptyp] OR Dataset[ptyp] OR Meta-Analysis[ptyp] OR Randomized Controlled Trial[ptyp])	2
4	(("mesenchymal stem cells"[MeSH Terms] OR ("mesenchymal"[All Fields] AND "stem"[All Fields] AND "cells"[All Fields]) OR "mesenchymal stem cells"[All Fields] OR ("mesenchymal"[All Fields] AND "stromal"[All Fields] AND "cell"[All Fields]) OR "mesenchymal stromal cell"[All Fields]) AND GvHD[All Fields]) AND (Clinical Trial[ptyp] OR Case Reports[ptyp] OR Clinical Study[ptyp] OR	61

	Comparative Study[ptyp] OR Controlled Clinical Trial[ptyp] OR Dataset[ptyp] OR Meta-Analysis[ptyp] OR Randomized Controlled Trial[ptyp])	
5	("mesenchymal stem cells"[MeSH Terms] OR ("mesenchymal"[All Fields] AND "stem"[All Fields] AND "cells"[All Fields]) OR "mesenchymal stem cells"[All Fields] OR ("mesenchymal"[All Fields] AND "stem"[All Fields] AND "cell"[All Fields]) OR "mesenchymal stem cell"[All Fields]) AND ("graft vs host disease"[MeSH Terms] OR ("graft"[All Fields] AND "vs"[All Fields] AND "host"[All Fields] AND "disease"[All Fields]) OR "graft vs host disease"[All Fields] OR ("graft"[All Fields] AND "versus"[All Fields] AND "host"[All Fields] AND "disease"[All Fields]) OR "graft versus host disease"[All Fields]) AND (Clinical Trial[ptyp] OR Case Reports[ptyp] OR Clinical Study[ptyp] OR Comparative Study[ptyp] OR Controlled Clinical Trial[ptyp] OR Dataset[ptyp] OR Meta-Analysis[ptyp] OR Randomized Controlled Trial[ptyp])	105
6	MSC[All Fields] AND ("graft vs host disease"[MeSH Terms] OR ("graft"[All Fields] AND "vs"[All Fields] AND "host"[All Fields] AND "disease"[All Fields]) OR "graft vs host disease"[All Fields] OR ("graft"[All Fields] AND "versus"[All Fields] AND "host"[All Fields] AND "disease"[All Fields]) OR "graft versus host disease"[All Fields]) AND (Clinical Trial[ptyp] OR Case Reports[ptyp] OR Clinical Study[ptyp] OR Comparative Study[ptyp] OR Controlled Clinical Trial[ptyp] OR Dataset[ptyp] OR Meta-Analysis[ptyp] OR Randomized Controlled Trial[ptyp])	60

Table S2. Characteristics of the included studies that evaluated the use of MSC for GvHD prophylaxis.

Study	Patient category	Patient #		Age (years)		Sex (M/F)		GvHD prophylaxis (n)		MSC source, infusion route	MSC dose/kg (10^6)	MSC donor type	Primary disease (n)		Follow-up time
		MSC	Ctrl	MSC	Ctrl	MSC	Ctrl	MSC	Control				MSC	Control	
Lazarus 2005[1]	Adults	46		44.5 (19-61)		24/22		CsA+MTX		BM, IV	2.12 (1-5)	HLA-identical sibling	AML (5) ALL (7) CLL (2) CML (14) MM (2) NHL (10) MDS (5) Other (1)		2 yrs
Ball 2007[2]	Children	14	47	8 (1-16)	7.1 (1-17)	9/5	28/19	ns		BM, IV	1.6 (1-3.3)	HLA-identical sibling	ALL (4) AML (7) Immune deficiencies (2) Other nonmalignant disorders (1)	ALL (21) AML (12) CML (2) Immune deficiencies (2) Other nonmalignant disorders (10)	MSC: 3-28 mos; Ctrl: 32-110 mos
Ning 2008[3]	Adults	10	15	38 (17-52)	37 (16-61)	9/1	13/2	CsA+MTX		BM, IV	0.34 (0.03-1.53)	HLA-identical sibling	AML (6) CML (2) ALL (1) MDS (1)	AML (7) CML (4) MDS (1) NHL (1) ALL (2)	36.6 mos (0.6-44)
MacMillan 2009[4]	Children	8	23	7.5 (0.2-16)	ns	ns		CsA+mPSL		BM, IV	Day 0: 2.1 (0.9-5) Day 21: 2.02 (0.06-5.3)	HLA-identical	ALL (6) AML (2)	ALL, AML	6.8 yrs
Baron 2010[5]	Children and adults	20	16	58 (21-69)	55 (10-69)	14/6	13/3	Tacro+MMF	CsA+MMF Tacro+MMF	BM, ns	ns	Unrelated	AML (7) CLL (1) NHL (5) HL (1) MM (5) Plasmablastic leukaemia (1)	AML (2) NHL (5) MDS (2) MM (6) Metastatic renal cell carcinoma (1)	1 yr
Zhang 2010[6]	Adults	12		37 (21-53)		8/4		CsA+MTX		BM, IV	1.65 (1.34-2.24)	HLA-identical sibling	SAA (2) CML (5) ANLL (3) ALL (2)		29-57 mos
Bernardo 2011[7]	Children	13	39	2 (0.8-1.4)	4 (0.8-17)	7/6	24/15	CsA (2) CsA+steroids (11)	CsA (9) CsA+steroids (27) CsA+MTX (3)	BM, IV	1.9 (1-3.9)	Parental	ALL (6) AML (1) MDS/JMML (2) HLH (4)	ALL (21) AML (5) MDS/JMML (9) HLH (4)	MSC: 28 mos (19-38) Ctrl: 42 mos (16-134)
Liu 2011[8]	Children and adults	27	28	30 (14-46)	31.5 (12-48)	20/7	19/9	CsA+MTX		BM, ns	0.3-0.5	Related (4) Unrelated (23)	ALL (16) AML (7) CML (5)	ALL (8) AML (13) CML (6)	23.7 mos (0.7-33.5)
Lee 2013[9]	Children	7	9	6.9 (8.3-12.5)	9.5 (8.4-18.7)	ns		CsA+MMF		UC, ns	2.7 (1, 5)	Unrelated	ns		2 yrs
Wu 2013[10]	Children	5	9	8.8 (4.1-11.6)	7.8 (4.8-10.1)	3/2	4/5	CsA+mPSL+ATG		UC, IV	5.76 (3.12-8.21)	ns	Nonmalignant hematological disease (3) leukemia (2)	Nonmalignant hematological disease (4) leukemia (5)	27 mos (24-31)
Wu 2013b[11]	Children	8	12	9.8 (3.2-12.1)	8.5 (3.6-13.1)	5/3	6/6	CsA+mPSL+ATG		UC, IV	7.19 (2.44-10.12)	ns	ALL (3) AML (5)	ALL (5) AML (7)	MSC: 16.5 (11-27) Control: 18.5 mos (12-31)
Kharbanda 2014[12]	Children	6		10 (8-18)		5/1		CsA+MMF		BM, ns	Day 0: 2.08 (1.72-2.97) Day 2: 2.06 (1.5-2.97)	Related (1) Unrelated (5)	SCD (4) β-Thalassemia (2)		ns

Shipounova 2014[13]	Adults	39	38	34 (17-63)		40/37		CsA+MTX MMF PSL		BM, IV	Day 0 and 28 (19-54): 1.2 (0.9-1.65)	HLA-identical sibling	Hematological malignancies		> 50 mos
Gao 2016[14]	Children and adults	62	62	ns		29/33	30/32	CsA+MMF+MTX		UC, ns	ns	ns	AML (43) ALL (14) MDS (5)	AML (42) ALL (16) MDS (4)	51 mos (24-70)
Liu 2017[15]	Children and adults	44		24 (8-47)		29/15		CsA+MMF+MTX		BM, IV	Day 0 and 14: 3.6 (3.2-4.1)	Related	SAA		12 mos (0.9 – 30.8)
Wang 2019[16]	Children	35		11.5 (3-18)		17/18		CsA+MMF+MTX		BM, ns	Day 0 and 14: 1	ns	SAA		22 mos (3.5-37)

Ctrl, control; MSC, mesenchymal stromal cells; M, male; F, female; mos, months; yrs, years; IV, intravenously, BM, bone marrow; UC, umbilical cord; ns, not specified; ATG, antithymocyte globulin ; CsA, cyclosporin A; mPSL, methylprednisolone; MMF, mycophenolate mofetil; MTX, methotrexate; Tacro, tacrolimus; ALL, acute lymphoblastic leukemia; AML, acute myeloid leukemia; ANLL, acute nonlymphocytic leukemia; CLL, chronic lymphocytic leukemia; CML, chronic myeloid leukemia; HL, Hodgkin's lymphoma; HLH, hemophagocytic lymphohistiocytosis; JMML, juvenile myelomonocytic leukemia; MDS, myelodysplastic syndrome; MM, multiple myeloma; NHL, non- Hodgkin's lymphoma; SCD, sickle cell disease; SAA, severe aplastic anemia.

Table S3. Characteristics of the included studies using MSC for the treatment of aGvHD and/or cGvHD.

Study	GvHD	Patient category	Patient #	Age (years)	Sex (M/F)	GvHD prophylaxis (n)	GvHD treatment	MSC source, infusion route	MSC dose/kg ($x10^6$)	Dose number	First infusion after HSCT (days)	MSC donor type (n)	Primary disease (n)	Follow-up time
Ringdèn 2006[17]	Acute	Children and adults	8	56 (8-61)	7/1	CsA + MTX (6)/PSL (1)/MMF (1)	PSL Additional: mPSL, ATG, MTX, MMF, PUVA, IFL, DAC	BM, ns	1 (0.7-9)	1-2	+114.6 (32-251)	HLA-id sib (2) Haplo-id (5) Unrelated matched (4)	ALL (3) AML (2) Myeloma (1) Solid tumor (2)	13 mos (2-36)
Fang 2007[18]	Acute	Adults	6	38.5 (22-49)	3/3	CsA+MTX+MMF	CsA+PSL	AD, IV	1	1-2	+99.2 (65-243)	Unrelated mismatched (4) Haplo-id (2)	AML(3) ALL(3)	40 mos (18-90)
Le Blanc 2008[19]	Acute	Adults	30	22 (0.5-64)	34/21	CsA (4) + MTX (38)/MMF (5)/PSL (6) Other (2) ATG (30) ALG (1) alemtuzumab (5)	PSL (55) Additional: CsA/Tacro (55) MMF (10) DAC (1) +IFL (4) ETP+PUVA (1) Cy (3) ATG (2) RIT (1) Extracorporeal photochemotherapy (10)	BM, IV	1.4 (0.4-9)	1-5	+103 (27-533)	HLA-id sib (5) Haplo-id (18) Unrelated mismatched (69)	AML (8) ALL (12) CML (7) CLL (2) JMML (4) MM (2) MPD (1) MDS (6) Lymphoma (1) Nonmalignant disorders (10) Solid tumor (2)	16 mos (1.5-64)
		Children	25											
Kebriaei 2009[20]	Acute	Adults	31	52 (34-67)	21/10	CsA (6) Tacro (10) +MMF/MTX (11)	mPSL/PSL CsA/Tacro and/or MMF	BM, IV	2 or 8	2	+34 (14-121)#	Unrelated unmatched	AML/MDS (14) NHL (5) CLL (4) ALL (4) MF (2) MM (1) HL (1)	90 days
Von Bonin 2009[21]	Acute	Adults	13	58 (21-69)	7/6	CsA (3) + MTX (7)/MMF (2) Tacro+MMF (1)	PSL (12) mPSL (9) IFL (6) MTX (3) Pentostatin (1)	BM, ns	0.9 (0.6-1.1)	2 (1-5)	+41 (20-91)	Unrelated	MM (3) CLL (2) MDS (1) NHL (2) MPD/MDS (1) AML (2) SAA (1) ALL (1)	207 days (116-261)
Arima 2010[22]	Acute	Adults	3	48 (39-64)	1/2	Tacro+MTX+MMF+mPSL (2) CsA+MTX (1)	mPSL+MMF+ATG+ETP (1) mPSL+Tacro (1) PSL+MMF+ETP+CsA (1)	BM, IA	0.22 (0.08-0.5)	1-2	+124.7 (96-158)	Related	AML (2) MDS (1)	60 days (20 – 103)
Prasad 2011[23]	Acute	Children	12	6.6 (0.4-15)	10/2	CsA (1) + mPSL (3)/MTX (2)/MMF (4) Tacro+mPSL (1) Tacro+MTX+ATG (1)	mPSL+ DAC+MMF (1) +Tacro (1) +IFL (1) +BUD (1)/ MMF+DAC+Tacro (1) +IFL+RIT (1)/ IFL+CsA+ETP (1) IFL+ECP+Tacro (1) CsA+BUD (1)/MMF+IFL (1) +OKT3 (1)	BM, IV	2 or 8	8 (2-21)	+98 (45-237)	Unrelated	AML (4) ALL (2) ALD (1) M OP (1) HLH (2) Hurler (1) MPD (1)	611 days (427-1111)
Remberger 2012[24]	Acute	Adults	15	57 (34-65)	11/4	CsA+MTX (14) Others (1)	Steroids Additional: DAC (3) IFL (4) sirolimus (3) RIT (2) MMF (1)	BM, IV	1.4 (0.65-2)	ns	ns	ns	Acute Leukemia (5) Other hematological malignancy (8) Solid Tumor (2)	2 yrs
Ball 2013[25]	Acute	Children	37	7 (0.7-18)	19/18	CsA (10) + MTX (13)/mPSL (11) Other (3)	mPSL or equivalent	BM, ns	2 (0.9-3)	2 (1-13)	+13 (5-85)#	Haplo-id related (3) Unrelated (34)	ALL (10) AML (6) MDS (7) JMML (5) Immune deficiencies (3) Other no-malignant disorders (6)	2.9 yrs (1.7 mos-6.7 yrs)

Muroi 2013[26]	Acute	Adults	13	49.7 (29-62)	4/9	CsA (1) +MTX (4) Tacro (2) + sMTX (5)/MMF (1)	mPSL or PSL	BM, ns	2	8 (3-12)	+50.2 (33-108)	Unrelated	MDS (3) AML (4) ALL (3) FL (1) CLL (1) MM (1)	96 weeks
		Children	1	4	1/0	CsA+MTX				12	+78		JMML	
Resnick 2013[27]	Acute	Children and adults	50	19 (1-69)	28/22	CsA+ MTX (8)/ATG (7)/MMF(10)	mPSL (50) Additional: CsA (50) Tacro (40) rapa (8) MMF (40) ATG (27) anti-CD25 mAb (9) ECP (16)	BM, IA/IV	4.27 (1.70±1.10)	1-4	+47 (11-180)	Related/ Unrelated	ALL (10) JMML (2) XLP (2) AML (15) AML/MDS (7) NHL (4) WAS (1) OP (1) SCID (1) CML (2) CLL (2) SAA (1) MM(1) SCD (1)	6 mos
Kurtzberg 2014[28]	Acute	Children	75	7.8 (0.2-17.5)	44/31	CsA, sirolimus, MTX	mPSL or equivalent Additional: ETP (11) Pentostatin (4) IFL (41) DAC (19) Denileukin difitox (1) Alemtuzumab (2) ATG (4) MMF (18) Tacro (32) RIT (7)	BM, IV	2	10 (1-20)	+48	Unrelated	ALL (18) AML (16) CML (1) MDS (7) NHL (1) Genetic disease (16) Other (16)	100 days
Sánchez-Guijo 2014[29]	Acute	Adults	25	<40 (6) 40-60 (8) >60 (11)	13/12	Tacro+ rapa (10)/MTX (5)/MMF (1) CsA+MMF (3) CsA+MTX (3)+alemtuzumab (2) Velcade+rapa (1)	mPLS	BM, IV	1.1 (0.7-1.31)	2-4	+2.5 (1-7)#	Unrelated	AML (6) MDS (7) HL (4) NHL (3) MM (3) CLL (1) ALL (1)	12 mos
Yin 2014[30]	Acute	Adults	9	38 (20-71)	5/4	n.s	mPSL	BM, IV	2	3	+58.5 (24-1521)	Unrelated	AML (3) ALL (2) MDS (1) DLBCL (1) CGD (1) CTCL (1)	300 days (222-401)
Boome 2015[31]	Acute	Children and adults	48	44.9 (1.3-68.9)	31/17	n.s	CsA+PSL	BM, ns	1.7 (0.9-2.5)	1-4	+85 (24-436)	Unrelated	Myeloid neoplasms (27) Lymphoid neoplasms (16) Non-malignant disorders (5)	1 yr
Zhao 2015[32]	Acute	Children and adults	28	26 (14-54)	19/9	CsA+MTX CsA+MTX+ATG and/or MMF	mPSL+CNI Additional: MTX (8) MMF (10) ATG (12) Cy (13) anti-CD25 mAb (17)	BM, ns	1	4 (2-8)	ns	Unrelated	AML (9) ALL (14) CML (4) NHL (1)	322.5 days (42-1054)
Dalowski 2016[33]	Acute	Adults	58	55 (19-71)	33/25	CNI (53) mTOR inhibitor (2) Others (3)	mPSL or equivalent	BM, IV	0.99 (0.45-2.08)	2	+12 (6-62)#	Unrelated	AML (25) CLL (9) ALL (5) MDS (5) NHL (4) MM (4) Others (6)	55.4 mos (32.8-60)
Erbey 2016[34]	Acute	Children	33	7 (3-18)	17/16	CsA (13) +MTX (11)/MMF (6) MMF (3)	Corticosteroids + second-line treatment	BM	1.18 (0.54-2.80)	1-4	+18 (5-88)#	HLA-id (1) Unrelated (67)	Malign (16) Nonmalign (17) diseases	335 days (41-1319)
Kuçi 2016[35]	Acute	Children and adults	26	6.5 (1-19)	10/16	CsA (6) +MTX (10)/MMF (3) MMF (1) Without (6)	Steroids Additional: MMF+CsA (8) + ETP (2)/Everolimus (1)/ Basiliximab+Sirolimus (1)/ Tacro (1) +Pentostatin+ECP (1)/ ECP (4) +Everolimus (2)/IFL+Basiliximab (1) BUD+Tacro (1)	BM	2.6 (0.9-7.7)	3 (1-9)	ns	Unrelated	ALL (8) AML (5) MDS (6) RMS (2) SCN (2) SAA (1) CGD (19) DBA (1)	2 yrs

							MMF+ECP (1) MMF+MTX+ETP+IFL (1) IFL+Tacro (1) +MMF (1)							
Muroi 2016[36]	Acute	Children and adults	25	33 (5-66)	15/10	MTX+Tacro (17)/CsA (3) Others (5)	PSL or mPSL	BM, IV	2	9 (4-16)	ns	Unrelated	ALL (9) AML (8) MDS (3) Others (5)	52 weeks
Dotoli 2017[37]	Acute	Adults	30	28 (1-72)	28/18	CsA+MTX/MMF (22) CsA+corticosteroids (5) Tacro+MTX/MMF (7) Other (12)	mPSL Additional: Basiliximab (13) ETP (7) MMF (4) P (4)	BM, IV	6.81 (0.98-29.78)	3 (1-7)	+25 (6-153)[#]	Unrelated	AML/ALL (22) MDS (7) SAA (4) NHL (3) Paroxysmal nocturnal hemoglobinuria (3) CML (2) ALD (2) Sickle cell anemia (1) Fanconi anemia (1) Congenital erythropoietic porphyria (1)	48.07 mos (21.4-88.4)
		Children	16											
Salmenniemi 2017[38]	Acute	Adults	18	45 (21-66)	11/7	CNI/everolimus+MTX (8)+MMF (7) Other (3)	Corticosteroids	BM, ns	2 (1.4-2.7)	5 (1-6)	+58 (15-641)	Unrelated	Malignant or non malingnant disease	767 days (74-1270)
		Children	8	8 (2-14)	5/3	CNI/everolimus+MTX (3)+MMF (1) Other (4)								
Keto 2018[39]	Acute	Adults	16	46 (21-66)	9/7	CNI/everolimus+MTX CNI/everolimus+MTX+MMF	mPSL	BM	2 (1.4-2.7)	4 (1-6)	+114.3 (25-438)	Unrelated	ns	ns
Stoma 2018[40]	Acute	Adults	34	37.5 (30-43)	11/23	CsA, MTX, Tacro	mPSL+CNI Additional: ATG	BM (24) AD (10), ns	1.32 (0.87-2.16)	ns	+91 (31-131)	Unrelated (24) Related (10)	AML (12) HL (6) CML (9) MDS (5) ALL (2)	1 yr
Kebriaei 2019[41]	Acute	Adults	149	43.8 (0.5-70)	91/72	CsA (71)+ Cellcept (26)/MTX (25) Tacro (86) Cellcept (54) Other-MTX (60) Other-nonMTX (14)	mPSL or equivalent Additional: ATG (35) MMF (27) IFL (29) ETP (23) DAC (16) Pentostatin (4) Denileukin diftitox (7) ECT (5) alemtuzumab (1) None (8)	BM	2	1-16	ns	Unrelated	ns	180 days
		Children	14											
Müller 2008[42]	Acute	Children	2	9 (4-14)	ns	n.s	PSL+mPSL+MMF+OKT3 (1) +ATG+Tacro (1)	BM, IV	1.31 (0.4-3)	1-2	+89 (80-98)	Unrelated (1) Related (1)	CML (1), AML (1)	<29 mos
	Chronic		3	15.7 (15-17)			CsA+MMF+PSL (1) +ETP (2)		2.28 (1.4-3)		+381.3 (222-672)	Unrelated (2) Related (1)	CML (1) ALL (1) SAA (1)	
Lucchini 2010[43]	Acute	Children	8	10.25 (4-15)	6/2	CsA+ATG+MTX (5) CsA+ATG+MMF (1) CsA+PSL+MMF (1) CsA+PSL+MTX (1)	Sterorids Additional: CsA+ETP (2) +MMF (2) +ECP (1) Tacro+ETP+ECP (1) ATG+ECP+IFL (1) MMF+Tacro+ETP+ ATG (1)	BM, IV	1.21 (0.7-3.7)	2 (1-5)	+64.1 (34-98)	Unrelated	ALL (4) Thalassemia (2) AML (1) Fanconi anemia (1)	8 mos (4-18)
	Chronic		3	6 (5-12)	2/1	CsA+MTX (1) +ATG (2)	Steroids Additional: CsA+MMF+ ECP+Tacro+BUD (1)/ ETP (1) +ECP+azatioprine (1)		1.15 (1-1.4)	1-2	+142 (68-210)		ALL (3)	
Pérez-Simon 2011[44]	Acute	Adults	10	37.3 (21-66)	5/5	CsA+MTX (2)/MMF (1) Tacro+MMF (1)/rapa (2) Tacro+MTX (1) +ATG (2) None (1)	n.s	BM, IV	1.3 (0.6-2.9)	2 (1-4)	ns	Unrelated (9) Related (1)	ns	ns
	Chronic		8	43.4 (21-66)	4/4	CSA+MMF (1)/MTX (4) Tacro+MMF (2)/MTX (1)			0.9 (0.2-1.20)	1 (1-4)		Unrelated (5) Related (3)		
Herrmann 2012[45]	Acute	Adults	12	43.5 (21-61)	8/4	CNI+MTX	PSL/mPSL+CNI Second line treatment: ETP	BM, ns	1.7-2.3	4 (2-19)	+4 mos (1-10)	Unrelated (10) Non-HLA matched sib	ALL (1) MDS (1) CLL(2) AML (3) NHL (2) RAEBT (1)	3 yrs

												(1) Matched sib (1)	CML (1) PRV to MF (1)	
	Chronic		7	43.2 (31-53)	5/2		CNI+MMF+PSL			7 (2-11)	+45.1 mos (14-89)	Unrelated (3) sib (3) Haploid son (1)	AML (4) ALL (2) CML (1)	
Introna 2014[46]	Acute	Adults	23	40.5 (19-65)*	16/9*	CsA+MTX (4) +ATG (12) Other (9)*	Steroids Additional: MMF,ETP, Pentostatin, CsA	BM, IV	1.5 (0.8-3.1)	2-11	+35 (5-1535)*#	Unrelated	Malignant (23) Nonmalignant (2)*	2.8 yrs
		Children	14	4.6 (1-18) **	11/4*	CSA+MTX+ATG (10) Other (5)**				2-7	+13 (4-277)** #		Malignant (13) Nonmalignant (2)**	
	Chronic	Adults	2	*	*	*				2-11	*#		*	
		Children	1	**	**	**				2-7	**#		**	
Yi 2016[47]	Acute	Adults	1	36 (21-52)	5/6	ns	n.s	BM, IV	1	1	ns	Unrelated	AML (6) ALL (2) CML (1) MM (1) MDS (1)	5 weeks
	Chronic		10											
Cetin 2017[48]	Acute	Adults	17	39.35 (21-51)	13/6	CsA (1) +MMF (1) CsA +MTX (13) +ATG (1)	CsA+MP (3) +MMF (5) +P (8) MMF+MP (1)	BM, AD, ns	1.64 (0.84-2.54)	2-7	ns	Unrelated	AML (11) ALL (5) MM (1) MDS (2)	6 mos
	Chronic		5	34.6 (24-47)	4/1	CsA+MTX (5)	CsA+MP+MMF (3) +I (1) CsA+MP+P+I (1)			3			AML (3) MDS (1) CML (1)	
Weng 2010[49]	Chronic	Adults	19	29.4 (18-39)	14/5	CsA+MMF+MTX	PSL (18) Additional: CsA (4) +MTX (3) + Penicillanmine (1) MMF (1) +CsA (2) + Tha1 (1) Tacro (1) +Azathioprine (1) + MTX (1) Tacro+BUD (1) Tacro+MMF (1) CsA+Penicillanmine+Thal (1)	BM, IV	0.6 (0.23-1.42)	2 (1-5)	ns	Unrelated	CML (8) AML (6) ALL (4) MDS (1)	697 days (81-1294)
Zhou 2010[50]	Chronic	Adults	4	41 (38-43)	3/1	CsA (1) + MMF (1)/MTX (1)/Tacro (1)	TDM+PSL+Tacro (2) PSL+CsA (1) TDM+PSL+MMF (1)	BM, intra-BM	1.6 (1-2.2)	6 (4-8)	ns	Unrelated	AML (2) ALL (1) MM (1)	14.1 mos (4.6 - 23)
Jurado 2017[51]	Chronic	Adults	14	48 (24-60)	7/7	n.s	CsA+PSL	AD, IV	1 or 3	1	ns	Unrelated	Lymphomas (9) Acute leukemias (4) Myeloma (1)	56 weeks

M, male; F, female; mos, months; yrs, years; IA, intra-artenial; IV, intravenously, AD, adipose tissue; BM, bone marrow;; UC, umbilical cord; id, identical; sib, sibling ns, not specified; ALG, antilymphocyte globulin; ATG, antithymocyte globulin; BUD, budesonide; CNI, calcineurin inhibitor; CsA, cyclosporin A; Cy, cyclophosphamide; DAC, daclizumab; ECT, extra-corporeal photopheresis; ETP, etanercept; I, imatinib mesylate; IFL, infliximab; mPSL, methylprednisolone; MMF, mycophenolate mofetil; MTX, methotrexate; P, photopheresis; PSL, prednisolone; PUVA, psoralen and UVA light; Rapa, rapamycin; RIT, Rituximab; Tacro, tacrolimus; TDM, thalidomide; ALD, adrenoleukodystrophy; ALL, acute lymphoblastic leukemia; AML, acute myeloid leukemia; CGD, chronic granulomatous disease; CLL, chronic lymphocytic leukemia; CML, chronic myeloid leukemia; CTCL, cutaneous T-Cell Lymphoma; DBA, Diamond-Blackfan anemia; DLBCL, diffuse large B-cell lymphoma; FL, follicular lymphoma; HL, Hodgkin's lymphoma; HLH, hemophagocytic lymphohistiocytosis; JMML, juvenile myelomonocytic leukemia; MDS, myelodysplastic syndrome; MF, myelofibrosis; MM, multiple myeloma; M OP, malignant osteopetrosis; MPD, Myeloproliferative disorder; NHL, non- Hodgkin's lymphoma; PRV, polycythemia rubra vera; RAEBT, refractory anaemia with excess of blasts in transformation; RMS, rhadomyosarcoma; SAA, severe aplastic anemia; SCD, sickle cell disease; SCID, severe combined immune deficiency; XLP, X-linked lymphoproliferative disease; WAS, Wiscott Aldrich syndrome. / or; # Days after GvHD diagnosis. * and ** Results for acute and chronic GvHD.

Table S4. Characteristics of the control group for included studies using MSC for the treatment of GvHD.

Study	GvHD	Patient category	Patient #	Age (years)	Sex (M/F)	GvHD prophylaxis (n)	GvHD treatment (n)	Primary disease (n)	Follow-up time
Remberger 2012[52]	Acute	Adults	13	48 (27-60)	9/4	CsA+MTX (9) Others (4)	DAC (3) IFL (3) sirolimus (3) MMF (4) ATG (2) PUVA (3) ECP (4)	Acute Leukemia (4) Other hematological malignancy (6) Solid tumor (3)	2 yrs
Zhao 2015[53]	Acute	Children and adults	19	29 (14-50)	7/12	CSA+MTX CSA+MTX+ATG/MMF	mPSL+CNI Additional: MTX (7) MMF(9) ATG (13) Cy (11) CD25 mAb (12)	AML (7) ALL (10) CML (2)	265 days (49-1312)
Dalowski 2016[33]	Acute	Adults	35	50 (21-67)	27/8	CNI (31) mTOR inhibitor (2) Others (2)	Ns	AML (12) CLL (3) ALL (7) MDS (4) NHL (3) MM (2) Others (4)	76.6 mos (58-102.1)
Jurado 2017[54]	Chronic	Adults	14	53 (29-65)	5/9	ns	PSL+ CsA (11)/tacro (3)	Lymphoma (5) Acute leukemia (5) Other (4)	56 weeks
Stoma 2018[40]	Acute	Adults	34	36 (29-45)	16/20	CSA, MTX, tacro	mPSL+CNI+ATG	AML (11) HL (10) CML (9) MDS (2) ALL (2)	ns
Kebriaeie 2019[41]	Acute	Adults	68	40 (0.5-70)	48/33	CsA (45)+ Cellcept (19)/MTX (14) Tacro (38) Cellcept (31) CsA+tacro+Cellcept (4) Other-MTX (24) Other-nonMTX (7)	mPSL or equivalent Additional: ATG (17) MMF (18) IFL (13) ETP (11) DAC (13) Pentostatin (9) Denileukin diftitox (2) ECT (2) None (3)	ns	180 days
		Children	13						

M, male; F, female; mos, months; yrs, years; ns, not specified; ATG, antithymocyte globulin; CNI, calcineurin inhibitor; CsA, cyclosporin A; Cy, cyclophosphamide; DAC, daclizumab; ECP, extracorporeal photopheresis; IFL, infliximab; mPSL, methylprednisolone; MMF, mycophenolate mofetil; MTX, methotrexate; PSL, prednisolone; PUVA, psoralen and UV A light; Tacro, tacrolimus; ALL, acute lymphoblastic leukemia; AML, acute myeloid leukemia; CLL, chronic lymphocytic leukemia; CML, chronic myeloid leukemia; HL, Hodgkin’s lymphoma; MDS, myelodysplastic syndrome; MM, multiple myeloma; NHL, non-Hodgkin’s lymphoma.

Table S5. Number of patients with aGvHD infused with MSC divided by grade and organs involved.

Study	Patient category	Patient #	Grade				Organs involved			Skin	Gut	Liver	Skin+ Gut	Skin+Liver	Gut+Liver	Skin+Gut+Liver
			I	II	III	IV	1	2	3							
Ringden 2006[17]	Children and adults	8		1	4	1	3	5		4	8	1	4		1	
Fang 2007[18]	Adults	6			2	4	1	4	1	5	3	4	2	2		1
Le Blanc 2008[19]	Adults	30		5	25	25	10	26	19	41	47	31	15	4	7	19
	Children	25														
Müller 2008[55]	Children	2		1	1			1	1	2	2	1	1			1
Kebriaei 2009[20]	Adults	31		21	7	3				13	11		7			
Von Bonin 2009[21]	Adults	13			2	11	2	8	3	6	11	10	1	2	5	3
Arima 2010[56]	Adults	3				3	1	1	1	2	3	2	1			
Lucchini 2010[43]	Children	8					6	2		6	4		2			
Pérez-Simón 2011[57]	Adults	10		3	3	4	6	2	2	4	10	2	2			
Prasad 2011[23]	Children	12			5	7	5	5	2	5	12	4	3		2	2
Herrmann 2012[45]	Adults	12		1	6	5	1	9	2	11	8	1	6			
Remberger 2012[52]	Adults	13			13		1	6	6	12	11	8	5	1		6
Ball 2013[25]	Children	37			37		5	11	21	30	32					
Muroi 2013[26]	Adults	13		9	4		6	7		9	9	2	6		1	
	Children	1			1			1			1	1			1	
Resnick 2013[58]	Children and adults	50		5	6	39	9	18	21	33	28					
Introna 2014[46]	Adults	23		2	17		9	16								
	Children	14		9	3		7	8								
Kurtzberg 2014[59]	Children	75					28	36	11	41	65	27	23		13	11
Sánchez-Guijo 2014[29]	Adults	25		7	14	4	10	11	4	17	20	7	8	1	2	4
Yin 2014[60]	Adults	9	2		7											
Boome 2015[31]	Children and adults	48		12	33	3				25	42	17				
Zhao 2015[53]	Children and adullts	28		4	8	16				21	24	16				
Dalowski 2016[33]	Adults	58	1	3	8	46	21	30	7							
Erbey 2016[34]	Children	33			5	28	5	14	14	32	29	14	14	0	0	14
Kuçi 2016[61]	Children and adults	26		1	12	13	9	9	8	16	24	9	6	0	3	8
Muroi 2016[62]	Children and adults	25			22	3	13	11	1	12	20	6	7	2	2	1
Cetin 2017[63]	Adults	19		7	6	6				16	8	14	3	4	2	1
Dotoli 2017[37]	Adults	30			10	36	13	21	12	44	41	16	17		4	12
	Children	16														

Salmenniemi 2017[38]	Adults	18		1	9	8	5	9	4	12	18	5				
Salmenniemi 2017[38]	Children	8		1	5	2	2	2	4	5	8	5				
Stoma 2018[40]	Adults	34		9	18	7				8	12	14				
Kebriaei 2019[41]	Children and adults	163		37	82	44	89	57	16	92	117	42	36	5	16	16
		**TOTAL (%)**		17.01	82.99		35.27	43.59	21.13	37.14	44.51	18.36	47.51	6.16	17.30	29.03

Table S6. Subgroup analysis for the overall survival of patients with aGvHD infused with MSC at last follow-up.

		Children			Adults			*P**	Studies (n)	Patients (n)	Total	*P***
		OS	Studies (n)	Patients (n)	OS	Studies (n)	Patients (n)					
Grade	II	0.00 (-0.98-0.98)	1	1	0.62 (0.40-0.84)	4	19	0.23	7	22	0.52 (0.29-0.75)	0.73
	III	0.70 (0.40-1.01)	3	7	0.45 (0.27-0.63)	5	29	0.16	10	73	0.66 (0.57-0.76)	
	IV	0.50 (0.10-0.90)	1	6	0.53 (0.24-0.82)	4	16	0.90	8	42	0.57 (0.41-0.72)	
Number of organs involved	1	0.67 (0.33-1.02)	3	16	0.51 (0.24-0.78)	6	25	0.45	11	60	0.56 (0.41-0.71)	0.50
	2	0.57 (0.37-0.78)	4	22	0.42 (0.32-0.52)	6	31	0.19	12	64	0.46 (0.38-0.54)	
	3	0.38 (0.10-0.67)	3	17	0.50 (0.15-0.85)	5	10	0.61	9	22	0.46 (0.27-0.65)	
Organ involved	Skin	0.63 (0.42-0.84)	5	86	0.52 (0.31-0.73)	6	53	0.44	13	138	0.64 (0.52-0.77)	0.37
	Gut	0.47 (0.20-0.75)	6	113	0.42 (0.12-0.71)	6	52	0.78	14	188	0.51 (0.35-0.66)	
	Liver	0.57 (0.27-0.87)	5	47	0.52 (0.22-0.83)	7	31	0.82	14	79	0.57 (0.41-0.72)	
Multiorgan	Skin+gut	0.56 (0.34-0.78)	4	19	0.41 (0.20-0.62)	5	24	n.a.	11	48	0.53 (0.39-0.67)	0.33
	Skin+liver	n.d.	n.d.	n.d.	0.50 (-0.48-1.48)	5	3	0.48	3	5	0.33 (-0.32-0.99)	
	Gut+liver	0.33 (-0.23-0.90)	2	3	0.40 (-0.04-0.84)	4	5	0.86	9	14	0.31 (0.04-0.58)	
	Skin+gut+ liver	0.38 (0.10-0.67)	3	17	0.43 (0.06-0.80)	4	8	0.85	8	18	0.44 (0.25-0.63)	

* P value for the differences between patient category. ** P value for the differences between subgroups. n.a., not applicable; n.d., not defined.

Table S7. Subgroup analysis for the overall response of patients with aGvHD infused with MSC.

		Children			Adults			*P**	Studies (n)	Patients (n)	Total	*P***
		OR	Studies (n)	Patients (n)	OR	Studies (n)	Patients (n)					
Grade	II	0.54 (-0.17-1.25)	2	10	0.75 (0.47-1.02)	5	21	0.59	8	33	0.72 (0.54-0.90)	0.17
	III	0.82 (0.46-1.18)	3	6	0.60 (0.46-0.75)	6	30	0.28	11	74	0.64 (0.55-0.73)	
	IV	0.71 (0.38-1.04)	1	7	0.70 (0.50-0.91)	5	24	0.98	9	49	0.78 (0.66-0.89)	
	III-IV	0.69 (0.46-0.91)	4	16	0.79 (0.70-0.89)	7	71	0.39	12	143	0.76 (0.69-0.83)	
Number of organs involved	1	0.73 (0.46-1.00)	3	15	0.72 (0.53-0.92)	8	27	0.98	14	67	0.67 (0.56-0.78)	0.14
	2	0.77 (0.56-0.98)	4	22	0.74 (0.55-0.93)	7	37	0.81	14	82	0.80 (0.70-0.90)	
	3	0.59 (0.35-0.82)	3	17	0.68 (0.37-0.98)	6	11	0.97	11	37	0.65 (0.47-0.83)	
Organ involved	Skin	0.81 (0.69-0.94)	5	96	0.85 (0.72-0.98)	6	61	0.71	11	171	0.85 (0.77-0.93)	0.004
	Gut	0.60 (0.49-0.71)	4	85	0.75 (0.63-0.87)	5	55	0.07	10	163	0.72 (0.62-0.81)	
	Liver	0.56 (0.39-0.74)	4	60	0.71 (0.56-0.86)	6	39	0.21	10	108	0.63 (0.53-0.74)	
Multiorgan	Skin+gut	0.74 (0.43-1.05)	4	19	0.67 (0.42-0.92)	6	25	0.73	13	62	0.78 (0.67-0.90)	0.61
	Skin+liver	n.d.	n.d.	n.d.	1.00 (0.31-1.69)	2	3	n.d.	3	5	0.75 (0.26-1.24)	
	Gut+liver	0.67 (0.10-1.23)	2	3	0.60 (0.12-1.08)	5	10	0.86	9	18	0.73 (0.43-1.02)	
	Skin+gut+ liver	0.59 (0.35-0.82)	3	17	0.62 (0.26-0.99)	5	9	0.87	10	33	0.63 (0.45-0.82)	

* P value for the differences between patient category. ** P value for the differences between subgroups. n.d., not defined.

Table S8. Subgroup analysis for the complete response of patients with aGvHD infused with MSC.

		Children			Adults			*P**	Studies (n)	Patients (n)	Total	*P***
		CR	Studies (n)	Patients (n)	CR	Studies (n)	Patients (n)					
Grade	II	0.44 (0.12-0.76)	1	9	0.51 (0.38-0.63)	6	43	0.70	8	54	0.49 (0.37-0.60)	0.39
	III	0.00 (-0.69-0.69)	2	5	0.47 (0.29-0.65)	6	30	0.20	10	74	0.41 (0.29-0.53)	
	IV	0.29 (-0.05-0.63)	1	7	0.49 (0.45-0.53)	5	24	0.25	9	48	0.37 (0.28-0.46)	
	III-IV	0.18 (-0.05-0.41)	1	11	0.41 (0.22-0.60)	8	92	0.13	12	159	0.39 (0.25-0.52)	
Number of organs involved	1	0.63 (0.43-0.83)	4	20	0.36 (0.15-0.58)	3	27	0.08	14	72	0.43 (0.30-0.56)	0.34
	2	0.53 (0.24-0.82)	4	32	0.45 (0.24-0.66)	7	37	0.68	14	92	0.47 (0.33-0.60)	
	3	0.40 (0.20-0.60)	4	37	0.54 (0.18-0.90)	3	11	0.49	11	57	0.42 (0.26-0.59)	
Organ involved	Skin	0.51 (0.41-0.61)	5	96	0.72 (0.54-0.90)	7	77	0.05	12	187	0.64 (0.53-0.75)	0.001
	Gut	0.45 (0.23-0.68)	5	117	0.52 (0.22-0.83)	7	75	0.72	12	215	0.47 (0.33-0.61)	
	Liver	0.38 (0.26-0.49)	4	60	0.36 (0.22-0.51)	7	45	0.89	11	114	0.38 (0.29-0.47)	
Multiorgan	Skin+gut	0.64 (0.39-0.89)	3	18	0.45 (0.28-0.62)	6	25	0.22	12	61	0.47 (0.35-0.48)	0.25
	Skin+liver	n.d.	n.d.	n.d.	0.50 (-0.48-1.48)	2	3	n.d.	3	5	0.50 (0.01-0.99)	
	Gut+liver	0.00 (-0.69-0.69)	2	3	0.20 (-0.07-0.47)	5	10	0.60	9	18	0.23 (0.01-0.45)	
	Skin+gut+ liver	0.27 (0.04-0.51)	2	2	0.46 (0.06-0.87)	5	9	0.43	9	32	0.34 (0.14-0.54)	

* P value for the differences between patient category. ** P value for the differences between subgroups. n.d., not defined.

References.

1. Lazarus HM, Koc ON, Devine SM, et al. Cotransplantation of HLA-identical sibling culture-expanded mesenchymal stem cells and hematopoietic stem cells in hematologic malignancy patients. Biol Blood Marrow Transplant 2005;11(5):398–98.

2. Ball LM, Bernardo ME, Roelofs H, et al. Cotransplantation of ex vivo-expanded mesenchymal stem cells accelerates lymphocyte recovery and may reduce the risk of graft failure in haploidentical hematopoietic stem-cell transplantation. Blood 2007;110(7):2764–7.

3. Ning H, Yang F, Jiang M, et al. The correlation between cotransplantation of mesenchymal stem cells and higher recurrence rate in hematologic malignancy patients: Outcome of a pilot clinical study. Leukemia 2008;22(3):593–9.

4. MacMillan ML, Blazar BR, DeFor TE, Wagner JE. Transplantation of ex-vivo culture-expanded parental haploidentical mesenchymal stem cells to promote engraftment in pediatric recipients of unrelated donor umbilical cord blood: Results of a phase I-II clinical trial. Bone Marrow Transplant 2009;43(6):447–54.

5. Baron F, Lechanteur C, Willems E, et al. Cotransplantation of Mesenchymal Stem Cells Might Prevent Death from Graft-versus-Host Disease (GVHD) without Abrogating Graft-versus-Tumor Effects after HLA-Mismatched Allogeneic Transplantation following Nonmyeloablative Conditioning. Biol Blood Marrow Transplant 2010;16(6):838–47.

6. Zhang X, Li JY, Cao K, et al. Cotransplantation of HLA-identical mesenchymal stem cells and hematopoietic stem cells in Chinese patients with hematologic diseases. Int J Lab Hematol 2010;

7. Bernardo ME, Ball LM, Cometa AM, et al. Co-infusion of ex vivo-expanded, parental MSCs prevents life-threatening acute GVHD, but does not reduce the risk of graft failure

in pediatric patients undergoing allogeneic umbilical cord blood transplantation. Bone Marrow Transplant 2011;46(2):200–7.

8. Liu K, Chen Y, Zeng Y, et al. Coinfusion of mesenchymal stromal cells facilitates platelet recovery without increasing leukemia recurrence in haploidentical hematopoietic stem cell transplantation: A randomized, controlled clinical study. Stem Cells Dev 2011;

9. Lee SH, Lee MW, Yoo KH, et al. Co-transplantation of third-party umbilical cord blood-derived MSCs promotes engraftment in children undergoing unrelated umbilical cord blood transplantation. Bone Marrow Transplant 2013;

10. Wu KH, Sheu JN, Wu HP, et al. Cotransplantation of umbilical cord-derived mesenchymal stem cells promote hematopoietic engraftment in cord blood transplantation: A pilot study. Transplantation 2013;

11. Wu KH, Tsai C, Wu HP, Sieber M, Peng CT, Chao YH. Human application of ex vivo expanded umbilical cord-derived mesenchymal stem cells: Enhance hematopoiesis after cord blood transplantation. Cell Transplant 2013;

12. Kharbanda S, Smith AR, Hutchinson SK, et al. Unrelated donor allogeneic hematopoietic stem cell transplantation for patients with hemoglobinopathies using a reduced-intensity conditioning regimen and third-party mesenchymal stromal cells. Biol Blood Marrow Transplant 2014;20(4):581–6.

13. Shipounova IN, Petinati NA, Bigildeev AE, et al. Analysis of results of acute graft-versus-host disease prophylaxis with donor multipotent mesenchymal stromal cells in patients with hemoblastoses after allogeneic bone marrow transplantation. Biochem 2014;79(12):1363–70.

14. Gao L, Zhang Y, Hu B, et al. Phase II multicenter, randomized, double-blind controlled study of efficacy and safety of umbilical cord-derived mesenchymal stromal cells in the prophylaxis of chronic graft-versus-host disease after HLA-haploidentical stem-cell

transplantation. J Clin Oncol 2016;34(24):2843–50.

15. Liu Z, Zhang Y, Xiao H, et al. Cotransplantation of bone marrow-derived mesenchymal stem cells in haploidentical hematopoietic stem cell transplantation in patients with severe aplastic anemia: an interim summary for a multicenter phase II trial results (Bone marrow transplant. Bone Marrow Transplant 2017;52(7):1080.

16. Wang ZK, Yu HJ, Cao FL, et al. Donor-derived marrow mesenchymal stromal cell co-transplantation following a haploidentical hematopoietic stem cell transplantation trail to treat severe aplastic anemia in children. Ann Hematol 2019;98(2):473–9.

17. Ringdén O, Uzunel M, Rasmusson I, et al. Mesenchymal stem cells for treatment of therapy-resistant graft-versus-host disease. Transplantation 2006;81(10):1390–7.

18. Fang B, Song Y, Liao L, Zhang Y, Zhao RC. Favorable Response to Human Adipose Tissue-Derived Mesenchymal Stem Cells in Steroid-Refractory Acute Graft-Versus-Host Disease. Transplant Proc 2007;39(10):3358–62.

19. Le Blanc K, Frassoni F, Ball L, et al. Mesenchymal stem cells for treatment of steroid-resistant, severe, acute graft-versus-host disease: a phase II study. Lancet 2008;371(9624):1579–86.

20. Kebriaei P, Isola L, Bahceci E, et al. Adult Human Mesenchymal Stem Cells Added to Corticosteroid Therapy for the Treatment of Acute Graft-versus-Host Disease. Biol Blood Marrow Transplant 2009;15(7):804–11.

21. von Bonin M, Stölzel F, Goedecke A, et al. Treatment of refractory acute GVHD with third-party MSC expanded in platelet lysate-containing medium. Bone Marrow Transplant 2009;43(3):245–51.

22. Arima N, Nakamura F, Fukunaga A, et al. Single intra-arterial injection of mesenchymal stromal cells for treatment of steroid-refractory acute graft-versus-host disease: A pilot study. Cytotherapy. 2010;

23. Prasad VK, Lucas KG, Kleiner GI, et al. Efficacy and Safety of Ex Vivo Cultured Adult Human Mesenchymal Stem Cells (Prochymal^{TM}) in Pediatric Patients with Severe Refractory Acute Graft-Versus-Host Disease in a Compassionate Use Study. Biol Blood Marrow Transplant 2011;17(4):534–41.

24. Remberger M, Ringdén O. Treatment of severe acute graft-versus-host disease with mesenchymal stromal cells: A comparison with non-MSC treated patients. Int. J. Hematol. 2012;

25. Ball LM, Bernardo ME, Roelofs H, et al. Multiple infusions of mesenchymal stromal cells induce sustained remission in children with steroid-refractory, grade III-IV acute graft-versus-host disease. Br J Haematol 2013;163(4):501–9.

26. Muroi K, Miyamura K, Ohashi K, et al. Unrelated allogeneic bone marrow-derived mesenchymal stem cells for steroid-refractory acute graft-versus-host disease: A phase I/II study. Int J Hematol 2013;98(2):206–13.

27. Resnick IB, Barkats C, Shapira MY, et al. Treatment of severe steroid resistant acute GVHD with mesenchymal stromal cells (MSC). Am J Blood Res 2013;

28. Kurtzberg J, Prockop S, Teira P, et al. Allogeneic human mesenchymal stem cell therapy (Remestemcel-L, Prochymal) as a rescue agent for severe refractory acute graft-versus-host disease in pediatric patients. Biol Blood Marrow Transplant 2014;

29. Sánchez-Guijo F, Caballero-Velázquez T, López-Villar O, et al. Sequential Third-Party Mesenchymal Stromal Cell Therapy for Refractory Acute Graft-versus-Host Disease. Biol Blood Marrow Transplant 2014;20(10):1580–5.

30. Yin F, Battiwalla M, Ito S, et al. Bone marrow mesenchymal stromal cells to treat tissue damage in allogeneic stem cell transplant recipients: Correlation of biological markers with clinical responses. Stem Cells 2014;

31. Te Boome LCJ, Mansilla C, Van Der Wagen LE, et al. Biomarker profiling of steroid-

resistant acute GVHD in patients after infusion of mesenchymal stromal cells. Leukemia 2015;29(9):1839–46.

32. Zhao K, Lou R, Huang F, et al. Immunomodulation effects of mesenchymal stromal cells on acute graft-versus-host disease after hematopoietic stem cell transplantation. Biol Blood Marrow Transplant 2015;

33. Von Dalowski F, Kramer M, Wermke M, et al. Mesenchymal Stromal Cells for Treatment of Acute Steroid-Refractory Graft Versus Host Disease: Clinical Responses and Long-Term Outcome. Stem Cells 2016;34(2):357–66.

34. Erbey F, Atay D, Akcay A, Ovali E, Ozturk G. Mesenchymal stem cell treatment for steroid refractory graft-versus-host disease in children: A pilot and first study from Turkey. Stem Cells Int 2016;2016:1641402.

35. Kuçi Z, Bönig H, Kreyenberg H, et al. Mesenchymal stromal cells from pooled mononuclear cells of multiple bone marrow donors as rescue therapy in pediatric severe steroid-refractory graft-versus-host disease: A multicenter survey. Haematologica 2016;

36. Muroi K, Miyamura K, Okada M, et al. Bone marrow-derived mesenchymal stem cells (JR-031) for steroid-refractory grade III or IV acute graft-versus-host disease: a phase II/III study. Int J Hematol 2016;

37. Dotoli GM, De Santis GC, Orellana MD, et al. Mesenchymal stromal cell infusion to treat steroid-refractory acute GvHD III/IV after hematopoietic stem cell transplantation. Bone Marrow Transplant 2017;52(6):859–62.

38. Salmenniemi U, Itälä-Remes M, Nystedt J, et al. Good responses but high TRM in adult patients after MSC therapy for GvHD. Bone Marrow Transplant 2017;52(4):606–8.

39. Keto J, Kaartinen T, Salmenniemi U, et al. Immunomonitoring of MSC-Treated GvHD Patients Reveals Only Moderate Potential for Response Prediction but Indicates Treatment Safety. Mol Ther Methods Clin Dev 2018;9:109–18.

40. Stoma I, Karpov I, Krivenko S, et al. Mesenchymal stem cells transplantation in hematological patients with acute graft-versus-host disease: characteristics and risk factors for infectious complications. Ann Hematol 2018;97(5):885–91.

41. Kebriaei P, Hayes J, Daly A, et al. A Phase 3 Randomized Study of Remestemcel-L versus Placebo Added to Second-Line Therapy in Patients with Steroid-Refractory Acute Graft-versus-Host Disease. Biol Blood Marrow Transplant 2019;

42. Müller I, Kordowich S, Holzwarth C, et al. Application of multipotent mesenchymal stromal cells in pediatric patients following allogeneic stem cell transplantation. Blood Cells, Mol Dis 2008;

43. Lucchini G, Introna M, Dander E, et al. Platelet-lysate-expanded mesenchymal stromal cells as a salvage therapy for severe resistant graft-versus-host disease in a pediatric population. Biol Blood Marrow Transplant 2010;16(9):1293–301.

44. Pérez-Simon JA, López-Villar O, Andreu EJ, et al. Mesenchymal stem cells expanded in vitro with human serum for the treatment of acute and chronic graft-versus-host disease: Results of a phase I/II clinical trial. Haematologica 2011;

45. Herrmann R, Sturm M, Shaw K, et al. Mesenchymal stromal cell therapy for steroid-refractory acute and chronic graft versus host disease: A phase 1 study. Int J Hematol 2012;95(2):182–8.

46. Introna M, Lucchini G, Dander E, et al. Treatment of graft versus host disease with mesenchymal stromal cells: A phase I study on 40 adult and pediatric patients. Biol Blood Marrow Transplant 2014;20(3):375–81.

47. Yi HG, Yahng SA, Kim I, et al. Allogeneic clonal mesenchymal stem cell therapy for refractory graft-versus-host disease to standard treatment: A phase I study. Korean J Physiol Pharmacol 2016;

48. Cetin M, Akyol G, Gonen ZB, et al. Additional infusions of mesenchymal stem cells

improve response rate in multidrug-resistant GvHD patients. Bone Marrow Transplant. 2017;

49. Weng JY, Du X, Geng SX, et al. Mesenchymal stem cell as salvage treatment for refractory chronic GVHD. Bone Marrow Transplant 2010;

50. Zhou H, Guo M, Bian C, et al. Efficacy of Bone Marrow-Derived Mesenchymal Stem Cells in the Treatment of Sclerodermatous Chronic Graft-versus-Host Disease: Clinical Report. Biol Blood Marrow Transplant 2010;

51. Jurado M, De La Mata C, Ruiz-García A, et al. Adipose tissue-derived mesenchymal stromal cells as part of therapy for chronic graft-versus-host disease: A phase I/II study. Cytotherapy 2017;

52. Remberger M, Ringdén O. Treatment of severe acute graft-versus-host disease with mesenchymal stromal cells: A comparison with non-MSC treated patients. Int J Hematol 2012;96(6):822–4.

53. Zhao K, Lou R, Huang F, et al. Immunomodulation effects of mesenchymal stromal cells on acute graft-versus-host disease after hematopoietic stem cell transplantation. Biol Blood Marrow Transplant 2015;21(1):97–104.

54. Jurado M, De La Mata C, Ruiz-García A, et al. Adipose tissue-derived mesenchymal stromal cells as part of therapy for chronic graft-versus-host disease: A phase I/II study. Cytotherapy 2017;19(8):927–36.

55. Müller I, Kordowich S, Holzwarth C, et al. Application of multipotent mesenchymal stromal cells in pediatric patients following allogeneic stem cell transplantation. Blood Cells, Mol Dis 2008;40(1):25–32.

56. Arima N, Nakamura F, Fukunaga A, et al. Single intra-arterial injection of mesenchymal stromal cells for treatment of steroid-refractory acute graft-versus-host disease: A pilot study. Cytotherapy 2010;12(2):265–8.

57. Pérez-Simon JA, López-Villar O, Andreu EJ, et al. Mesenchymal stem cells expanded in vitro with human serum for the treatment of acute and chronic graft-versus-host disease: Results of a phase I/II clinical trial. Haematologica 2011;96(7):1072–6.

58. Resnick IB, Barkats C, Shapira MY, et al. Treatment of severe steroid resistant acute GVHD with mesenchymal stromal cells (MSC). Am J Blood Res 2013;3(3):225–38.

59. Kurtzberg J, Prockop S, Teira P, et al. Allogeneic human mesenchymal stem cell therapy (Remestemcel-L, Prochymal) as a rescue agent for severe refractory acute graft-versus-host disease in pediatric patients. Biol Blood Marrow Transplant 2014;20(2):229–35.

60. Yin F, Battiwalla M, Ito S, et al. Bone marrow mesenchymal stromal cells to treat tissue damage in allogeneic stem cell transplant recipients: Correlation of biological markers with clinical responses. Stem Cells 2014;32(5):1278–88.

61. Kuçi Z, Bönig H, Kreyenberg H, et al. Mesenchymal stromal cells from pooled mononuclear cells of multiple bone marrow donors as rescue therapy in pediatric severe steroid-refractory graft-versus-host disease: A multicenter survey. Haematologica 2016;101(8):985–94.

62. Muroi K, Miyamura K, Okada M, et al. Bone marrow-derived mesenchymal stem cells (JR-031) for steroid-refractory grade III or IV acute graft-versus-host disease: a phase II/III study. Int J Hematol 2016;103(2):243–50.

63. Cetin M, Akyol G, Gonen ZB, et al. Additional infusions of mesenchymal stem cells improve response rate in multidrug-resistant GvHD patients. Bone Marrow Transplant 2017;52(5):783–5.
